# Metabarcoding of marine nematodes – evaluation of reference datasets used in tree-based taxonomy assignment approach

**DOI:** 10.3897/BDJ.4.e10021

**Published:** 2016-09-21

**Authors:** Oleksandr Holovachov

**Affiliations:** ‡ Swedish Museum of Natural History, Stockholm, Sweden

**Keywords:** Nematoda, metabarcoding, alignment, phylogeny inference, taxonomy assignment, 18S rRNA, OTU, tree-based approach.

## Abstract

**Background:**

Metabarcoding is becoming a common tool used to assess and compare diversity of organisms in environmental samples. Identification of OTUs is one of the critical steps in the process and several taxonomy assignment methods were proposed to accomplish this task. This publication evaluates the quality of reference datasets, alongside with several alignment and phylogeny inference methods used in one of the taxonomy assignment methods, called *tree-based* approach. This approach assigns anonymous OTUs to taxonomic categories based on relative placements of OTUs and reference sequences on the cladogram and support that these placements receive.

**New information:**

In tree-based taxonomy assignment approach, reliable identification of anonymous OTUs is based on their placement in monophyletic and highly supported clades together with identified reference taxa. Therefore, it requires high quality reference dataset to be used. Resolution of phylogenetic trees is strongly affected by the presence of erroneous sequences as well as alignment and phylogeny inference methods used in the process. Two preparation steps are essential for the successful application of tree-based taxonomy assignment approach.

1. Curated collections of genetic information do include erroneous sequences. These sequences have detrimental effect on the resolution of cladograms used in tree-based approach. They must be identified and excluded from the reference dataset beforehand.
2. Various combinations of multiple sequence alignment and phylogeny inference methods provide cladograms with different topology and bootstrap support. These combinations of methods need to be tested in order to determine the one that gives highest resolution for the particular reference dataset.

Completing the above mentioned preparation steps is expected to decrease the number of unassigned OTUs and thus improve the results of the tree-based taxonomy assignment approach.

## Introduction

Metabarcoding of living organisms is on the rise as the cost of Next Generation Sequencing goes down and processing pipelines improve (Bittleston et al. 2015, Cowart et al. 2015, Fonseca et al. 2010, Leray et al. 2015 to name a few). Identification of anonymous metabarcodes clustered in Operational Taxonomic Units (OTUs) is one of the critical steps in the analysis, and several different taxonomy-assignment methods were proposed to accomplish this task (Berger et al. 2011, Edgar 2010, Lanzén et al. 2012, Matsen et al. 2010, Munch et al. 2008, Stark et al. 2010, Wang et al. 2007). They can be grouped into four different categories: alignment-based, probabilistic, tree-based and phylogeny-based (Holovachov et al. unpublished). And while the performance of alignment-based, probabilistic and phylogeny-based methods have been thoroughly evaluated in their original publications, tree-based methods are often applied with great confidence and without critical evaluation (exception Austerlitz et al. 2009), relying on previous extensive and thorough evaluation of same algorithms done in the past (see for example Hall (2004) and others).

Tree-based taxonomy assignment approach (called *phylogenetic approach* in Huson et al. (2009)) evaluates similarity between anonymous OTUs and identified reference sequences by analyzing the position of each individual OTU relative to reference sequences on the cladogram, and the bootstrap support that this position receives. Multiple sequence alignment of short query reads (OTUs) with reference sequences is done *de-novo*, and the dataset is usually trimmed to the barcode size. The cladogram is built using one of the available phylogeny inference algorithms (Huson et al. 2009). Position of each OTU on the cladogram is than evaluated individually, taking into consideration its sister and neighboring taxa, and the support that OTUs placement receives. Only OTUs placed within monophyletic and highly supported clades can be assigned to taxonomic categories with confidence. Taxonomic identities of OTUs placed in paraphyletic and polyphyletic taxa are often impossible to evaluate correctly – such OTUs should be treated as unidentified.

There are several issues that needs to be considered when applying tree-based taxonomy assignment approach. First is the size and properties of the barcoding region. Most of the barcoding regions used in the past range in size between 250 and 700 bases and are expected to include fewer phylogenetically informative sites comparing to loci normally used for phylogenetic analysis (for example 1600-1800 bases long 18S rRNA). Barcoding regions are purposely chosen to include hypervariable sites (Floyd et al. 2002), which are difficult to align using progressive alignment algorithms. Ambiguous alignments will effect the resulting phylogenetic tree, usually in a negative way (Holovachov et al. 2015). Secondly, the criteria used to assign OTUs to clades and equivalent to them taxa, such as bootstrap values or tree topology, are not always clearly defined in the publications (but see Austerlitz et al. 2009). The third and last issue, the quality of reference datasets, is actually relevant for all taxonomy assignment methods. It may refer to the presence of erroneous (poor quality or incorrectly identified) and misplaced (correctly identified but placed in the wrong taxonomic category) sequences (Schnell et al. 2015) or sequences that have less than 100% overlap with query OTU sequences.

As will be discussed in detail elsewhere (Holovachov et al. unpublished), if OTUs of marine nematodes can not be identified to species or even genus level due to incompleteness of reference databases, the largest taxon that they can be placed into, and that can still provide sufficient information for ecological studies is the family. However, before using tree-based approach to assign OTUs of marine nematodes to the families, (Holovachov et al. unpublished), its possible drawbacks must be thoroughly evaluated. Such as the impact of the reference dataset, or the alignment or phylogeny inference algorithms on the quality of the results.

The goal of this paper is to estimate how well the cladogram based solely on the barcoding region (in this case it is the 5' end of 18S rRNA molecule) resolves and supports families of marine nematodes. It will be accomplished by evaluating the results obtained by analyzing several reference datasets and by using different combinations of alignment and phylogeny inference algorithms. The first dataset will include all relevant sequences that fulfill specific criteria described below; the second dataset will exclude all sequences that are found to be questionable; the third dataset will also exclude all sequences that do not have sufficient coverage with the barcoding region used in Haenel et al. (unpublished) and Holovachov et al. (unpublished).

## Materials and Methods

### 1. Sequence data

SILVA database (Quast et al. 2012) is regularly used in metabarcoding studies to create reference database (Cowart et al. 2015, Haenel et al. unpublished). The entire Nematoda and Priapulida (to be used as an outgroup) section of it was downloaded on December of 2015. At the first step, all sequences were manually checked in order to remove animal parasitic and exclusively terrestrial nematode species, sequences already known to be incorrectly identified, unidentified sequences (environmental sequences), and non-nematode sequences placed within Nematoda. Examples of non-nematode taxa placed among nematodes include sequences from the phylum Tardigrada, Tubulichidae (phylum Annelida), Ricinulei, Limulidae and Poduridae (all three from the phylum Arthropoda), *Spironucleus torosa* (flagellate), uncultured fungus and even *Drosophila americana* and *Drosophila auraria*. Animal-parasitic taxa were not included in the current analysis with the exception of the family Mermithidae, some species of which are known from marine habitats (Tchesunov and Hope 1997). For the two terrestrial families that as exception include few marine species (Rhabditidae and Anguinidae), only 3-4 species were included. Sequences were further sorted according to the following criteria:

1. For the same species, longer sequences were chosen over shorter sequences.
2. Taxa identified to species level were chosen over taxa identified only to the genus level, considering that they both belong to the same genus.
3. All fully identified species for each genus were included.
4. For the same species (if available) no more than two sequences were included.
5. All available genera for every family of marine nematodes were included.
6. All families with at least two representative species were included.
7. All sequences that were missing 40 bases and more on the 5' end (equal to about 10% of of the length of the barcoding region) were excluded.

Suppl. material 1 lists GenBank accession numbers and classification (family, genus and species) of all sequences used in this study. Three dataset were be analyzed:

1. Complete dataset included all selected sequences.
2. "Filtered" dataset excludes species that are likely incorrectly identified and therefore consistently had negative impact on tree topology and clade support in the first analysis of the complete dataset.
3. "Long" dataset included only those sequences that had the same length as barcoding region (see section 2 of Materials and Methods, below), or were missing no more than 10 bases on the 5' end.

As a result, complete dataset includes 284 terminal taxa (280 nematode sequences and four outgroup taxa) belonging to 50 families or superfamilies (superfamilies Dorylaimoidea and Mononchoidea will be treated as whole, without subdivision into separate families in subsequent analyses). "Filtered" dataset was created by removing all erroneous sequences from the complete dataset. It includes 276 taxa (272 nematode sequences and four outgroup taxa) belonging to 50 nematode families. "Long" dataset was created based on the "filtered" dataset by removing sequences that had insufficient coverage. It includes 212 taxa (208 nematode sequences and four outgroup taxa) belonging to 48 nematode families or superfamilies. Families Anticomidae and Phanodermatidae are not presented in the "long" dataset because only one species of Anticomidae and none of Phanodermatidae satisfied the requirement of sufficient sequence length.

### 2. Barcoding region

**Figure 1.**
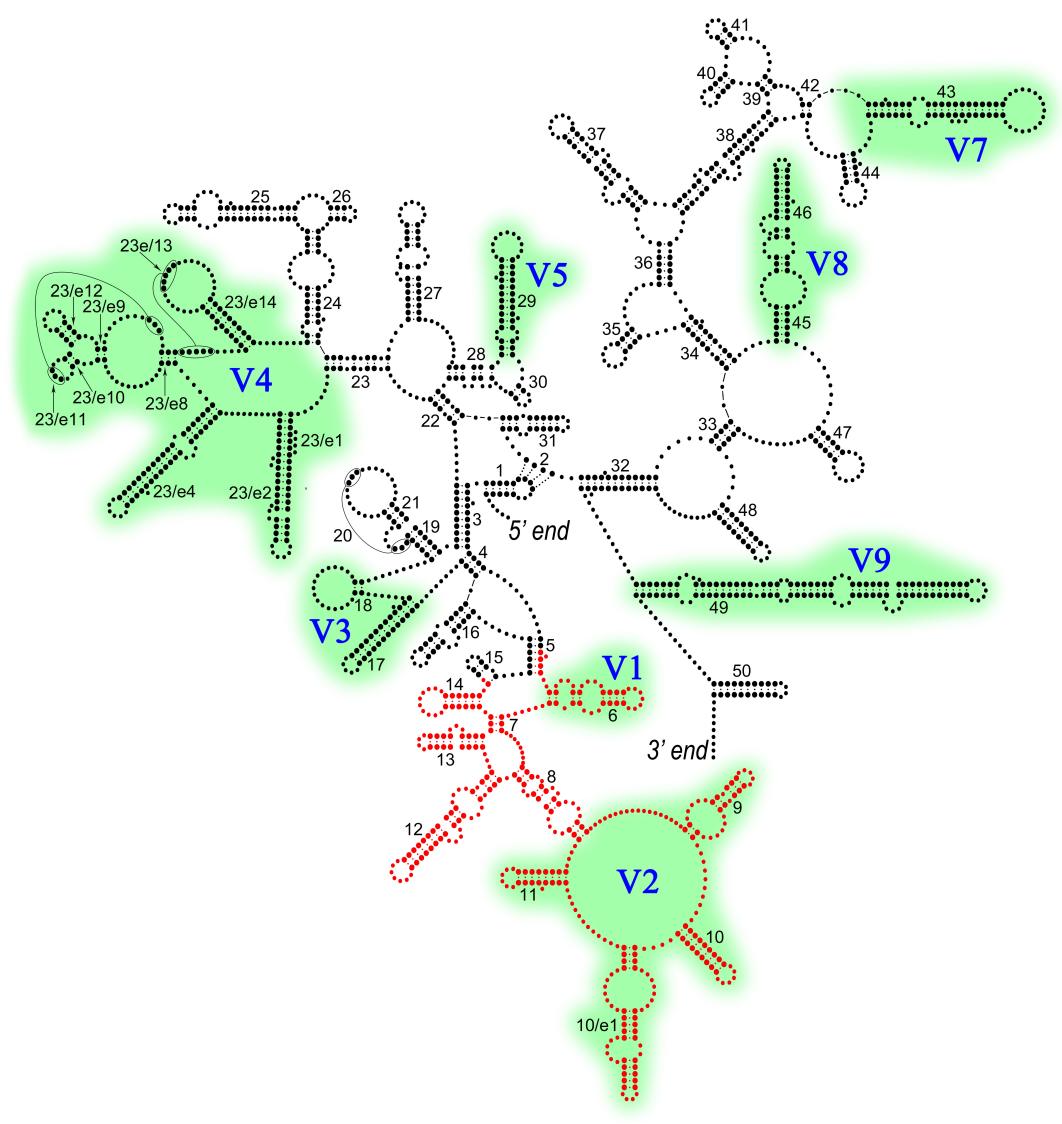
Barcoding region marked in red on the generalized secondary structure model of the nematode 18S rRNA (modified from Holovachov et al. 2015 with the permission from the publisher). Helices (1-23, 23/e1-23/e14, 24-50) are numbered according to Wuyts et al. (2002). Variable regions V1-V5 and V7-V9 (shaded in green) are numbered according to Neefs et al. (1990).

This publication evaluates the barcoding region of the 18S rRNA gene that includes V1 and V2 variable regions (Fig. 1) and is used in barcoding and metabarcoding studies of nematodes in particular (Floyd et al. 2002) and of marine meiofauna in general (Fonseca et al. 2010, Sinniger et al. 2016, Haenel et al. unpublished, Holovachov et al., unpublished).

### 3. Alignment

When applied to nematodes, following tools were used to align anonymous OTUs with reference datasets: Clustal-W/X (Bhadury and Austen 2010, Bhadury et al. 2006, Morise et al. 2012), MAFFT (De Ley et al. 2005, Kanzaki et al. 2012) and MUSCLE (Derycke et al. 2010, Sapkota and Nicolaisen 2015). Use of secondary-structure based alignment procedure has not been considered in the published record, due to it being extremely time consuming.

Six different alignment algorithms were tested: Clustal-O (Sievers et al. 2014), Clustal-W (Larkin et al. 2007), MAFFT (Katoh and Standley 2013), MUSCLE (Edgar 2004), PRANK (Löytynoja and Goldman 2010) and alignment downloaded directly from SILVA database (Quast et al. 2012). Clustal-W alignment was created using MEGA ver. 6 or 7 (Tamura et al. 2013). Clustal-O (http://www.ebi.ac.uk/Tools/msa/clustalo/), MAFFT (http://www.ebi.ac.uk/Tools/msa/mafft/), MUSCLE (http://www.ebi.ac.uk/Tools/msa/muscle/) and PRANK (http://www.ebi.ac.uk/goldman-srv/webPRANK/) alignments were created using respective online services at EMBL-EBI server (Li et al. 2015). Default settings for all alignments were used following the common practice. ARB-generated alignment was directly derived from the dataset downloaded from SILVA database (Quast et al. 2012); no changes were introduced to ARB-generated alignment except that gap-only sites were removed.

### 4. Phylogeny inference

Previously published studies on nematode barcoding or metabarcoding used Neighbor joining (Bhadury and Austen 2010, Bhadury et al. 2006, Derycke et al. 2010, Morise et al. 2012, Sapkota and Nicolaisen 2015), Maximum parsimony (De Ley et al. 2005) and Bayesian inference (Kanzaki et al. 2012) algorithms under default parameters. Following the general trend, and in order to replicate the methodology used by the predecessors, default settings were used for both phylogeny inference methods included in present analysis.

Neighbor joining trees were inferred using MEGA ver. 6 or 7 (Tamura et al. 2013) under Kimura 2 parameter model, transitions and transversions, uniform rates, pairwise deletion for missing data, bootstrap with 1000 replicates. Maximum likelihood trees were inferred using RAxML ver. HPC2 (Stamatakis 2014) of CIPRES Science Gateway portal (Miller et al. 2010) under GTRCAT model for bootstrapping with 1000 replicates. Maximum parsimony was not used for the following reasons: performing maximum parsimony analyses with sufficient number of bootstrap replicates turned out to be extremely time consuming using MEGA (Tamura et al. 2013) or MESQUITE (Maddison and Maddison 2015) and is unlikely to be used in such way in metabarcoding studies.

*Halicryptus spinulosus* sequence (AF342790) was used to root all phylogenetic trees. Monophyletic clades with bootstrap support of 70% and higher were considered well supported and fully resolved. Trees were visualized using FigTree (Rambaut 2015) and iTOL (Letunic and Bork 2016).

### 5. Evaluation criteria

As discussed in the Introduction, only anonymous OTUs placed within monophyletic and highly supported clades can be identified with confidence. Namely, OTUs that cluster within monophyletic clades with high bootstrap support are assigned certain taxonomic status (identification), e.g. barcodes clustered within the clade that is equivalent to a family "A" or a genus "B" in the classification may be identified as belonging to that family "A" or genus "B". On the other hand, anonymous OTUs clustered outside well supported monophyletic clades should be treated as unassigned. Therefore, following criteria were used to evaluate the quality of the results of each individual analysis (cladogram) produced in this study:

1. Number of nematode families resolved as monophyletic, paraphyletic or polyphyletic in each analysis. The therm "family" will be used to describe clades that are equivalent to family-level categories in nematode classification.
2. Bootstrap support that each monophyletic clade receives. Fully resolved clades, or families, are those that receive ≥70% bootstrap support.

It is expected that monophyletic clades with high bootstrap support are likely to remain such after combining the reference dataset with anonymous OTUs in possible future studies. To confirm this, and for the final comparison, two scenarios were chosen, the "worst case" (combination of dataset, alignment and phylogeny inference algorithms that produced the lowest number of highly supported monophyletic clades equivalent to families) and the "best case" (same but highest number of highly supported monophyletic clades equivalent to families). 25 pre-selected sequences (see Results, sections 4 and 5) were added to both alignments to create new datasets, both were re-aligned and re-analyzed following same "worst case" and "best case" settings. These pre-selected sequences represent species, which were either not included in the original complete dataset because of the criterium #2 (*taxa identified to species level were chosen over taxa identified only to the genus level, considering that they both belong to the same genus*); #4 (*for the same species no more than two sequences were included*); or because these sequences are available from GenBank but not yet included in the SILVA database. They were chosen to represent both well and poorly resolved families.

## Results

### 1.1. Complete dataset, Neighbor joining analysis

Cladograms inferred using Neighbor joining algorithm and six different types of alignment (Suppl. materials 2, 3, 4, 5, 6, 7) produced similar results, fully resolving at most 24 families out of 50 with Clustal-W, MAFFT and PRANK-based alignments, while Clustal-O-based alignment resolving the fewest 22 (Table 1). They have following features in common (Suppl. material 8):

1. Out of 50 nematode families and superfamilies included in this dataset, only 21 families are fully resolved as monophyletic and receive high bootstrap support (≥70%) in all six analyses.
2. Three families (Aphanolaimidae, Ceramonematidae and Chromadoridae) are also resolved as monophyletic, but their bootstrap support varies greatly between analyses, from the highest 94-96% to the lowest 43-60%.
3. The family Draconematidae is always resolved as monophyletic but with very low bootstrap support (35-66%).
4. Three families (Xyalidae, Tobrilidae and Phanodermatidae) may either have very low bootstrap support, or can be resolved as paraphyletic or polyphyletic.
5. Five families are consistently resolved as paraphyletic: the family Monhysteridae includes families Xyalidae and Sphaerolaimidae as ingroup clades; the family Desmodoridae is paraphyletic in relation to the family Draconematidae; the family Mermithidae is paraphyletic in relation to the superfamily Mononchoidea; the family Enoplidae consistently includes *Anticoma* sp. (AY692344) from the family Anticomidae; the clade that includes all members of the family Thoracostomopsidae also includes three unrelated taxa, namely *Parodontophora* sp. (AM234630) from the family Axonolaimidae, *Oncholaimus* sp. (KF591739) from the family Oncholaimidae and *Gammanema* sp. (KF591723) from the family Selachinematidae.
6. Seventeen families are always resolved as polyphyletic. Of these, only five families are consistently divided into two or three monophyletic and highly supported clades: the genus *Terschellingia* is always placed separately from the rest of Linchomoeidae; the genus *Prodesmodora* is consistently separated from the rest of Microlaimidae; the family Trefusiidae is always divided into terrestrial (*Trischistoma* and *Tripylina*) and marine (*Trefusia* and *Rhabdocoma*) clades; the family Oxystominidae is always split into three individual clades equivalent to the genera *Halalaimus*, *Oxystomina* and *Thalassoalaimus+Litinium*; the genus *Syringolaimus* is always placed separately from the rest of Ironidae; the family Anoplostomatidae is always split into clades represented by the genera *Anoplostoma* and *Chaetonema*. Two members of the family Diplopeltidae never form a monophyletic clade. Members of the families Oncholaimidae and Enchelidiidae are "mixed" in random manner. Paraphyly of other families is usually caused by separate placement of one or more of the sequences in the cladogram (see Results, section 1.3).

### 1.2. Complete dataset, Maximum likelihood analysis

The results were more variable between different alignments comparing to Neighbor joining analyses of the same set of data, with PRANK-based analysis resolving the maximum of 26 families, while Clustal-O, MUSCLE and SILVA-based analyses resolving only 21 each (Table 1). Cladograms inferred using Maximum likelihood algorithm and six different types of alignment (Suppl. materials 9, 10, 11, 12, 13, 14) have following features in common (Suppl. material 15):

1. Out of 50 nematode families and superfamilies included in this dataset, only 18 families are fully resolved as monophyletic and receive high bootstrap support (≥70%) in all six analyses.
2. Five families (Xyalidae, Mononchoidea, Mermithidae, Enoplidae and Leptosomatidae) are also resolved as monophyletic, but their bootstrap support varies greatly between analyses, from the highest 72-90% to the lowest 44-65%.
3. Nine families (Plectidae, Aphanolaimidae, Camacolaimidae, Ceramonematidae, Draconematidae, Chromadoridae, Tobrilidae, Enchelidiidae, Phanodermatidae) may either have very low to very high bootstrap support, or can be resolved as paraphyletic or polyphyletic.
4. Four familes are consistently resolved as paraphyletic: the family Monhysteridae includes families Xyalidae and Sphaerolaimidae as ingroup clades; the family Desmodoridae is paraphyletic in relation to the family Draconematidae; the clade that includes all members of the family Thoracostomopsidae also includes three unrelated taxa, namely *Parodontophora* sp. (AM234630) from the family Axonolaimidae, *Oncholaimus* sp. (KF591739) from the family Oncholaimidae and *Gammanema* sp. (KF591723) from the family Selachinematidae; the family Oxystominidae is a paraphyletic "grade" that includes as one of its monophyletic clades a range of other taxa.
5. Fourteen families are always resolved as polyphyletic. Of these, five families are consistently divided into two monophyletic and highly supported clades in exactly the same way as in previous (Neighbor joining) analysis (see Results, section 1.1). Two members of the family Diplopeltidae never form a monophyletic clade. Paraphyly of other families is usually caused by separate placement of one or more of the sequences on the cladogram (see Results, section 1.3).

### 1.3. Complete dataset, summary

Several sequences were consistently clustered outside their family clades and are thus considered problematic: *Anticoma* sp. (AY692344), *Parodontophora* sp. (AM234630), *Oncholaimus* sp. (KF591739), *Gammanema* sp. (KF591723), *Cyatholaimus* sp. (JN968214), *Longicyatholaimus* sp. (LK054720), *Pomponema* sp. (KF591743) and *Monoposthia costata* (AY854221). Visual examination of the alignment with congeneric taxa confirmed that the identity of these sequences is likely to be incorrect. Therefore, these sequences were excluded from the "filtered" dataset.

### 2.1. "Filtered" dataset, Neighbor joining analysis

Similar to 1.1, all six alignments produced comparable results (Suppl. materials 16, 17, 18, 19, 20, 21), resolving (≥70% bootstrap support) at most 30 families out of 50 with MUSCLE-based alignment, while Clustal-O-based alignment resolving the fewest 27 (Table 1) under same requirements. They have following features in common (Suppl. material 22):

1. Out of 50 nematode families included in this dataset, 27 families are fully resolved as monophyletic and receive high bootstrap support (≥70%) in all six analyses.
2. Five families (Aphanolaimidae, Ceramonematidae, Draconematidae, Selachinematidae and Tobrilidae) are also resolved as monophyletic, but their bootstrap support varies greatly between analyses, from the highest 44-94% to the lowest 19-68%.
3. Three families (Axonolaimidae, Xyalidae and Phanodermatidae) may either have very low to high bootstrap support, or can be resolved as paraphyletic or polyphyletic.
4. Two families (Camacolaimidae and Oxystominidae) are inconsistently resolved as either paraphyletic or polyphyletic.
5. Three familes are consistently resolved as paraphyletic: the family Monhysteridae includes families Xyalidae and Sphaerolaimidae as ingroup clades; the family Desmodoridae is paraphyletic in relation to the family Draconematidae; the family Mermithidae is paraphyletic in relation to superfamily Mononchoidea.
6. Two families (Oncholaimidae and Enchelidiidae) are combined in a highly supported clade, paraphyletic in relation to each other.
7. Eight families are always resolved as polyphyletic. Of these, five families are consistently divided into two monophyletic and highly supported clades in exactly the same way as in previous (complete dataset) analyses (see Results, sections 1.1 and 1.2). The family Leptolaimidae is also consistently split in two clades, but only one of these clades is monophyletic with high bootstrap support. The family Chronogastridae is split in either two or three weakly supported clades. Two members of the family Diplopeltidae never form monophyletic clade.

Removing erroneous sequences increased bootstrap support in 12-16 clades and resolution (clades became monophyletic) in 6-7 clades (Table 2); in two to five families bootstrap support decreased. Changes in bootstrap support of the families that were monophyletic in the Neighbor joining analysis of complete dataset (section 1.1) varied between -18 (decrease) and +64 (increase). Depending on the alignment, two to five families showed decrease in bootstrap support and six to nine showed increase, of which one or two families crossed the upper threshold (≥70% bootstrap support) and were fully resolved. Only in one case (Clustal-O-based alignment) small decrease of bootstrap support in the family Ceramonematidae (from 74% to 69%) placed it insignificantly below the 70% threshold.

Between six and seven families that were non-monophyletic (paraphyletic or polyphyletic) in the Neighbor joining analysis of complete dataset (section 1.1) were resolved as monophyletic after removing erroneous sequences. Bootstrap support for such families varied between 19% and 100%. As a result, 4-5 families crossed the upper threshold (≥70% bootstrap support) and were fully resolved. Thus, depending on the alignment, between five and seven new families were fully resolved (monophyletic with ≥70% bootstrap support) in the Neighbor joining analysis of the "filtered" dataset.

### 2.2. "Filtered" dataset, Maximum likelihood analysis

Similar to 1.2, the results were more variable between different alignments comparing to Neighbor joining analyses of the same set of data (Suppl. materials 23, 24, 25, 26, 27, 28), with PRANK-based analysis resolving the maximum of 31 families, while SILVA-based analysis resolving only 26 (Table 1). They have following features in common (Suppl. material 29):

1. Out of 50 nematode families included in this dataset, 24 families are resolved as monophyletic and receive high bootstrap support (>70%) in all six analyses.
2. Five families (Aphanolaimidae, Camacolaimidae, Xyalidae, Mononchoidea and Leptosomatidae) are also resolved as monophyletic, but their bootstrap support varies greatly between analyses, from the highest 74-89% to the lowest 37-69%.
3. Eight families (Plectidae, Axonolaimidae, Ceramonematidae, Draconematidae, Selachinematidae, Tobrilidae, Enchelidiidae and Phanodermatidae) may either have very low to high bootstrap support, or can be resolved as paraphyletic or polyphyletic.
4. Four familes are consistently resolved as paraphyletic: the family Monhysteridae includes families Xyalidae and Sphaerolaimidae as ingroup clades; the family Desmodoridae is paraphyletic in relation to the family Draconematidae; the family Oncholaimidae is paraphyletic in relation to the family Enchelidiidae; the family Oxystominidae is a paraphyletic "grade" that includes as one of its monophyletic clades a range of other taxa.
5. Eight families are always resolved as polyphyletic. Of these, five families are consistently divided into two monophyletic and highly supported clades in exactly the same way as in previous analyses (see Results, sections 1.1, 1.2 and 2.1). The family Leptolaimidae is also consistently split in two clades, but only one of these clades is monophyletic with high bootstrap support. Separation of the family Chronogastridae into two clades is inconsistent among different analyses. Two members of the family Diplopeltidae never form monophyletic clade.

Removing erroneous sequences increased bootstrap support in 14-26 clades and resolution in 5-8 clades (Table 3); 3-11 families received less bootstrap support, and in two cases one family was not resolved as monophyletic. Changes in bootstrap support of the families that were monophyletic in the Maximum likelihood analysis of complete dataset (section 1.2) and remain monophyletic here varied between -14 (decrease) and +58 (increase). Depending on the alignment, 3-11 families showed decrease in bootstrap support and 6-18 showed increase, of which one or two families crossed the upper threshold (≥70% bootstrap support) and were fully resolved. Only in cases of MAFFT and SILVA-based analysis decrease of bootstrap support in three families by 2-14% placed them both below the 70% threshold.

Between five and eight families that were non-monophyletic (paraphyletic or polyphyletic) in the Maximum likelihood analysis of complete dataset (section 1.2) were resolved as monophyletic after removing erroneous sequences. Bootstrap support for such families varied between 24% and 100%. As a result, 4-7 families crossed the upper threshold (≥70% bootstrap support) and were fully resolved. Thus, depending on the alignment, between five and eight new families were fully resolved (monophyletic with ≥70% bootstrap support) in the Maximum likelihood analysis of the "filtered" dataset.

### 2.3. "Filtered" dataset, summary

Exclusion of problematic sequences from the alignment (defined in section 1.3 above) resulted in substantial increase in resolution and support for many clades equivalent to family-level categories, because incorrect placement of each of them in previous analyses (complete dataset) affected resolution of two families, the one that they are identified with taxonomically, and the one that they are placed within in the phylogenetic analysis.

### 3.1. "Long" dataset, Neighbor joining analysis

Unlike in previous Neighbor joining analyses (sections 1.1 and 2.1), the results were more variable between different alignments (Suppl. materials 30, 31, 32, 33, 34, 35), with PRANK-based analysis resolving the maximum of 35 families, while SILVA-based analysis resolving only 32 (Table 1). They have following features in common (Suppl. material 36):

1. Out of 48 nematode families included in this dataset, 30 families are resolved as monophyletic and receive high bootstrap support (>70%) in all six analyses. In the case of the family Trefusiidae, which was resolved as polyphyletic (consisting of two distinct clades) during the analysis of "complete" dataset, the entire clade of marine taxa (*Trefusia* and *Rhabdocoma*) was exluded, leaving the second clade of terrestrial taxa (*Trischistoma* and *Tripylina*) in the dataset.
2. Eight families (Chronogastridae, Camacolaimidae, Axonolaimidae, Xyalidae, Ceramonematidae, Draconematidae, Selachinematidae and Tobrilidae) are also resolved as monophyletic, but their bootstrap support values vary greatly between analyses, from the highest 57-98% to the lowest 23-65%.
3. The family Microlaimidae is resolved as monophyletic with very low bootstrap support (19%) in one case only, polyphyletic in all other instances.
4. Four familes are consistently resolved as paraphyletic: the family Monhysteridae includes families Xyalidae and Sphaerolaimidae as ingroup clades; the family Desmodoridae is paraphyletic in relation to the family Draconematidae; the family Mermithidae is paraphyletic in relation to the superfamily Mononchoidea; the family Oncholaimidae is paraphyletic in relation to the family Enchelidiidae.
5. Five families are always resolved as polyphyletic. Of these, only two families are consistently divided into two monophyletic and highly supported clades: the genus *Terschellingia* is always placed separately from the rest of Linchomoeidae; the genus *Syringolaimus* is always placed separately from the rest of Ironidae. The family Leptolaimidae is also consistently split in two clades, but only one of these clades is monophyletic with high bootstrap support. Two members of the family Diplopeltidae never form a monophyletic clade. Similarly, three members of the family Oxystominidae never form a monophyletic clade.

Removing erroneous sequences improved bootstrap support in 15-16 clades and resolution in 5-7 clades (Table 4). Changes in bootstrap support of the families that were monophyletic in the Neighbor joining analysis of "filtered" dataset (section 2.1) varied between -16 (decrease) and +56 (increase). Depending on the alignment, 3-7 families showed decrease in bootstrap support and 9-10 showed increase, of which 0-3 families crossed the upper threshold (≥70% bootstrap support) and were fully resolved.

Between five and six families that were non-monophyletic (paraphyletic or polyphyletic) in the Neighbor joining analysis of "filtered" dataset (section 2.1) were resolved as monophyletic after removing erroneous sequences. Bootstrap support for such families varied between 19% and 99%. As a result, 4-5 families crossed the upper threshold (≥70% bootstrap support) and were fully resolved. Thus, depending on the alignment, between five and seven new families were fully resolved (monophyletic with ≥70% bootstrap support) in the Neighbor joining analysis of the "long" dataset.

### 3.2. "Long" dataset, Maximum likelihood analysis

In this case PRANK-based analysis again resolves the highest number of families (36 out of 50), and Clustal-O-based analysis resolves only 29 (Table 1). In general, cladograms produced using Maximum likelihood algorithm and six different types of alignment (Suppl. materials 37, 38, 39, 40, 41, 42) have following features in common (Suppl. material 43):

1. Out of 48 nematode families included in this dataset, 23 families are resolved as monophyletic and receive high bootstrap support (>70%) in all six analyses.
2. Eight families (Camacolaimidae, Xyalidae, Ceramonematidae, Mononchidae, Mermithidae, Tobrilidae, Leptosomatidae and Alaimidae) are also resolved as monophyletic, but their bootstrap support varies greatly between analyses, from the highest 69-95% to the lowest 43-69%.
3. Nine families (Plectidae, Chronogastridae, Aphanolaimidae, Axonolaimidae, Draconematidae, Microlaimidae, Selachinematidae, Trefusiidae and Anoplostomatidae) may either have variable (low-to-high) bootstrap support, or can be resolved as paraphyletic or polyphyletic.
4. Three familes are consistently resolved as paraphyletic: the family Monhysteridae includes families Xyalidae and Sphaerolaimidae as ingroup clades; the family Desmodoridae is paraphyletic in relation to the family Draconematidae; the family Oncholaimidae is paraphyletic in relation to the family Enchelidiidae.
5. Five families are always resolved as polyphyletic in exactly the same way as in previous (Neighbor joining) analysis of "long" dataset (see Results, section 3.1).

Removing short sequences improved bootstrap support in 13-15 clades and resolution in 2-6 clades (Table 5). Changes in bootstrap support of the families that were monophyletic in the Maximum likelihood analysis of "filtered" dataset (section 2.2) varied between -22 (decrease) and +45 (increase). Depending on the alignment, 7-14 families showed decrease in bootstrap support and 10-17 showed increase, of which 1-5 families crossed the upper threshold (≥70% bootstrap support) and were fully resolved. In three cases decrease of bootstrap support (between -1% and -22%) placed one family in each case below the 70% threshold.

Between one and four families that were non-monophyletic (paraphyletic or polyphyletic) in the Maximum likelihood analysis of "filtered" dataset (section 2.2) were resolved as monophyletic after removing short sequences. Bootstrap support for such families varied between 41% and 100%. As a result, 0-2 families crossed the upper threshold (≥70% bootstrap support) and were fully resolved. Thus, depending on the alignment, between two and six new families were fully resolved (monophyletic with ≥70% bootstrap support) in the Maximum likelihood analysis of the "long" dataset.

### 3.3. "Long" dataset, summary

Exclusion of incomplete sequences from the alignment resulted in increase in resolution and support for several clades equivalent to family-level categories, although in case of Maximum likelihood analysis, a number of clades were resolved as paraphyletic or polyphyletic, or lost bootstrap support below the 70% threshold.

### 4. "Worst case" scenario

**Figure 2.**
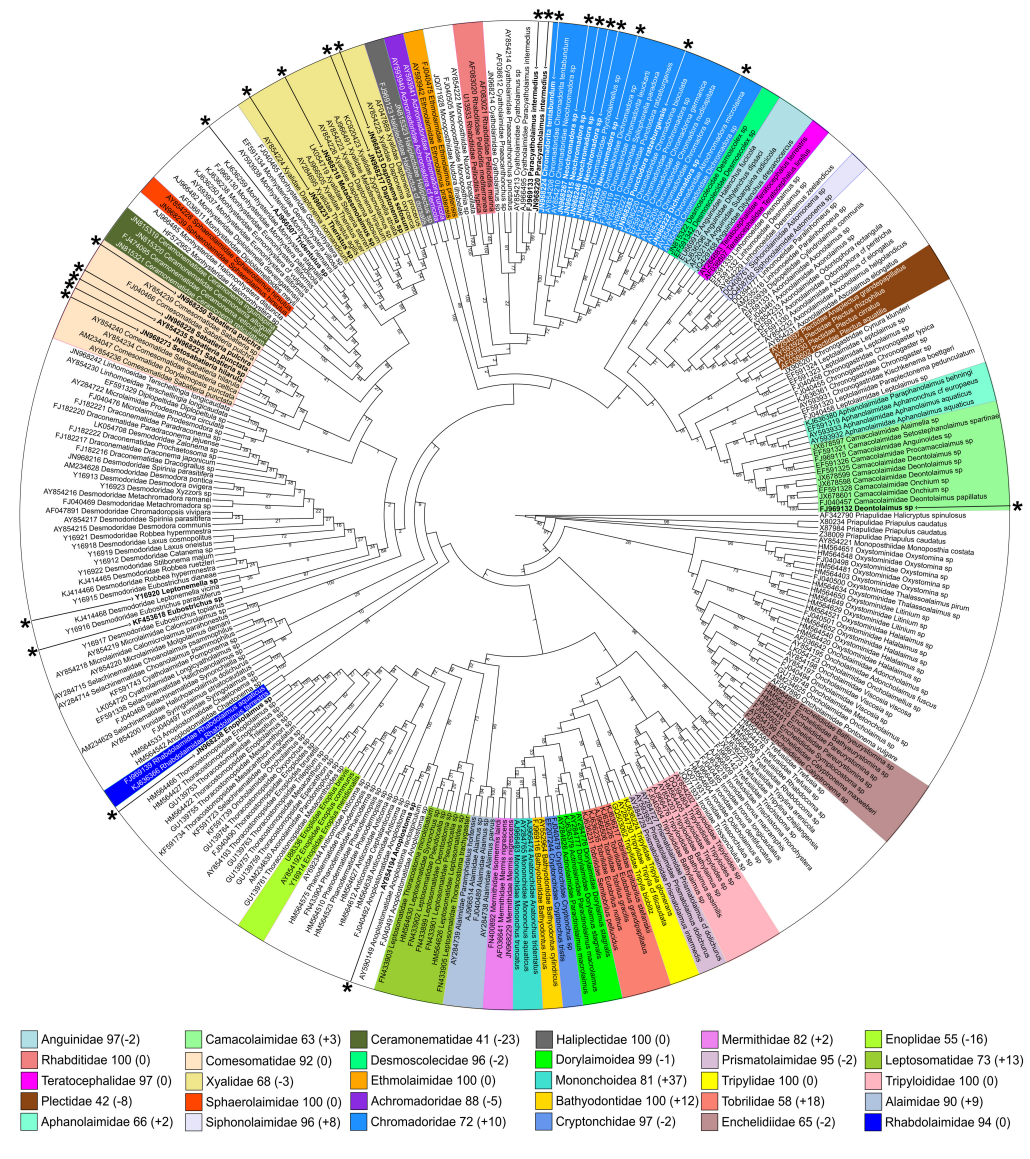
"Worst case" scenario – Maximum likelihood tree inferred using Clustal-O-based alignment of the complete dataset and 25 additional sequences (marked by asterisks). Numbers after family names in the legend indicate current bootstrap support for each clade and difference (in parenthesis) comparing to the original analysis (Clustal-O-based alignment, Maximum likelihood phylogeny inference, complete dataset) from the section 1.2 of the Results.

Preselected 25 sequences were added to original, complete dataset and re-analyzed using Clustal-O for alignment (phylogenies using Clustal-O-based alignment scored one of the worst in all analyses) and Maximum Likelihood for phylogeny inference. As expected, addition of new high quality sequences did not affect the resolution of the cladogram, but affected bootstrap support for monophyletic clades (Fig. 2). Changes in bootstrap support varied between -23% (decrease) and +37% (increase) thus affecting the 70% threshold for several clades: it decreased below threshold in two clades (Xyalidae from 71% to 68% and Enoplidae from 71% to 55%) and increased in three clades (Chromadoridae from 62% to 72%, Mononchoidea from 44% to 81% and Leptosomatidae from 60% to 73%).

Out of 25 added sequences, only 18 could be assigned to family-level categories based on their clustering withing monophyletic clades Table 6. Only nine of them are placed in clades that receive high (≥70%) bootstrap support, namely clades equivalent to the families Comesomatidae and Xyalidae. The remaining nine are placed within clades equivalent to the families Camacolaimidae (bootstrap support of 52%) and Chromadoridae (bootstrap support of 62%).

### 5. "Best case" scenario

**Figure 3.**
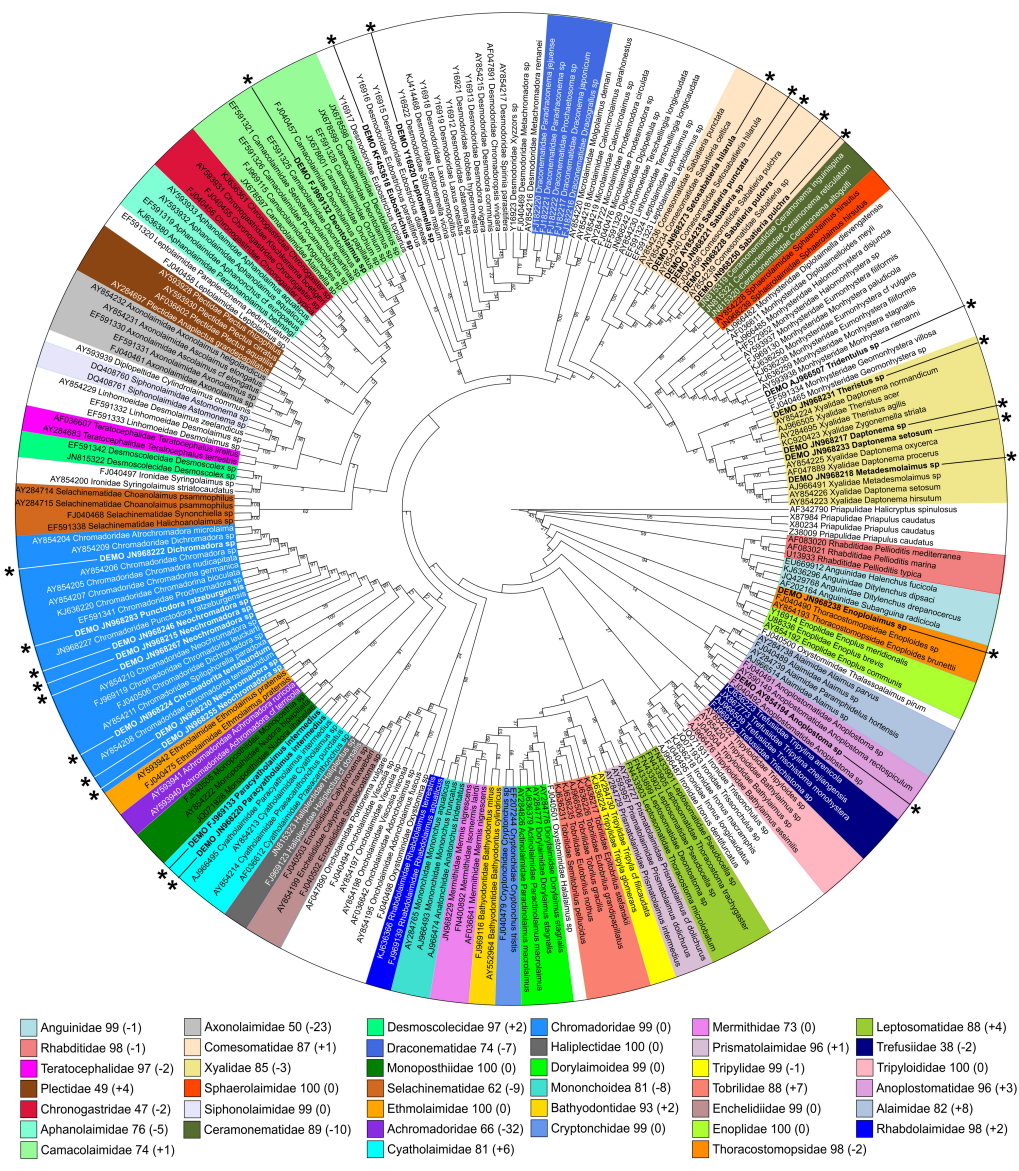
"Best case" scenario – Maximum likelihood tree inferred using PRANK-based alignment of the "long" dataset and 25 additional sequences (marked by asterisks). Numbers after family names in the legend indicate current bootstrap support for each clade and difference (in parenthesis) comparing to the original analysis (PRANK-based alignment, Maximum likelihood phylogeny inference, "long" dataset) from the section 3.2 of the Results.

Similar to "worst case" scenario described in the previous section, same preselected 25 sequences were added to "long" dataset and re-analyzed using PRANK for alignment and Maximum Likelihood for phylogeny inference. Just like in the previous example, addition of new high quality sequences did not affect the resolution of the cladogram, but affected bootstrap support for monophyletic clades (Fig. 3). Changes in bootstrap support varied between -32% (decrease) and +8% (increase) thus affecting the 70% threshold for several clades: it decreased below threshold in three clades: in Axonolaimidae from 73% to 50%, in Selachinematidae from 71% to 62% and in Achromadoridae from 98% to 66%.

Out of 25 added sequences, 22 could be assigned to family-level categories based on their clustering withing monophyletic clades Table 6. Moreover, all 22 of them are placed in clades that receive high (≥70%) bootstrap support (Camacolaimidae, Comesomatidae, Xyalidae, Cyatholaimidae, Chromadoridae, Anoplostomatidae and Thoracostomopsidae).

## Discussion

Results of a phylogenetic analysis are strongly determined not only by the alignment and phylogeny inference algorithms, but also by the quality of the input data. However, influence of poor quality sequences on different parts of the phylogenetic tree is not equal. Resolution and bootstrap support for some nematode families remained consistent throughout all analyses and was not affected by the presence of erroneous or short sequences. Large number of such families are unfortunately represented in current analysis by only few taxa (2-4 species), either due to limited availability of high quality sequences in the reference databases (Teratocephalidae, Siphonolaimidae, Sphaerolaimidae, Desmoscolecidae, Ethmolaimidae, Achromadoridae, Haliplectidae, Rhabdolaimidae, Bathyodontidae, Cryptonchidae), or because such families are mainly freshwater/terrestrial (Anguinidae, Rhabditidae, Mononchoidea, Dorylaimoidea, Prismatolaimidae, Tripylidae, Alaimidae). The latter are used here mainly to increase taxon coverage and sequence variability. The former are always represented by co-specific or co-generic taxa which monophyly is not questioned here. Both categories will not be further considered in the discussion.

The other families (marine and well represented with multiple sequences) that were always resolved as monophyletic in all analyses, independently from the alignment and phylogeny inference algorithms, are only Comesomatidae and Tripyloididae. There are three families that are resolved as polyphyletic in all analyses: Diplopeltidae, Linhomoeidae and Ironidae. These are similarly resolved in the analyses using nearly full-length 18S rRNA (van Megen et al. 2009) and are likely to be artificial assemblages. Resolution and support of other clades/families varied between different analyses and depended on the input datasets, alignment and phylogeny inference algorithms.

Higher taxa (clades equivalent to orders and classes in the nematode classification) were not fully resolved in any of performed analyses, with few exceptions. Order Monhysterida was fully resolved (monophyletic with high support) in all analyses using Maximum likelihood inference, and in some analyses using Neighbor joining inference (MAFFT-based alignment of the "filtered" dataset, Clustal-O, Clustal-W, MAFFT and PRANK-based alignments of the "long" dataset). Three terrestrial orders Dorylaimida, Rhabditida and Tylenchida, all of which were represented by very few sequences, were fully resolved in all analyses. Other orders were either poly- or paraphyletic, while bootstrap support for many basal dichotomies was lower than the required threshold.

### Alignments

Various multiple-sequence alignment software naturally produced alignments of varying quality, which affected the final outcome of all analyses in this comparison. Visual examination of alignment files showed that all of them, including alignments downloaded from SILVA database, were not able to cope with hypervariable regions of rRNA molecule, evidenced by the fact that identical (very similar) segments of sequences of closely related taxa (same genera) can be aligned differently. In this test, SILVA-based alignments produced some of the worst results, alongside Clustal-O and MAFFT. On the other hand, PRANK, Clustal-W and MUSCLE-based alignments produced cladograms with higher resolution and support, but the improvements are not always significant, and may not be observed for other barcoding regions or other groups of organisms.

### Phylogeny inference algorithms

Neighbor joining algorithm was shown to be effective in matching anonymous sequences to sequences that were preliminary identified (Bhadury et al. 2006). It is the most commonly used algorithm when it comes to identification of nematode barcodes and metabarcodes (Bhadury and Austen 2010, Bhadury et al. 2006, Derycke et al. 2010, Morise et al. 2012, Sapkota and Nicolaisen 2015) comparing to other methods. However, no thorough comparison has been done between different alignment algorithms and clustering approaches when applied to 5' end barcoding region of nematodes in general and of marine nematodes in particular. The results of this study show that alignment methods have higher impact on the results of phylogenetic analysis of the short barcoding region of marine nematodes than phylogeny inference algorithms – differences between Neighbor joining and Maximum likelihood analyses of were minor (Table 1), inconsistent and statistically insignificant.

### Problematic sequences

Improvement in the resolution and support achieved in the "filtered" dataset should be attributed to the exclusion of problematic (erroneous) sequences, namely: *Anticoma* sp. (AY692344), *Parodontophora* sp. (AM234630), *Oncholaimus* sp. (KF591739), *Gammanema* sp. (KF591723), *Cyatholaimus* sp. (JN968214), *Longicyatholaimus* sp. (LK054720), *Pomponema* sp. (KF591743) and *Monoposthia costata* (AY854221). Removing these sequences affected the resolution and support of both clades (families) that they are identified with taxonomically, and clades (families) that they were placed within during phylogeny inference. Moreover, if anonymous OTU is placed in the clade (monophyletic and highly supported) that includes problematic sequences, it might not be always possible to evaluate with confidence if it genuinely related to taxa representing majority of the clade, or if its placement is caused by similarity to a problematic sequence.

### Short sequences

Removing of short sequences increased support and resolution much less significantly, and at a cost of loss of reference data. In case of two families (Anoplostomatidae and Trefusiidae) one of the clades that defined these taxa as polyphyletic in the analyses of complete and "filtered" datasets, was completely absent in the "long" dataset, thus artificially defining Anoplostomatidae and Trefusiidae as monophyletic. In this case, it is important to find a balance between the number of incomplete sequences and completeness of the reference dataset.

### "Worst case" *versus* "Best case" scenarios

This comparison shows the differences in how the same set of "blind" taxa are assigned using two different, "worst case" Fig. 2 and "best case" Fig. 3 reference toolkit (dataset and algorithms). It is important to remember that adding blind taxa has double effect on the outcome of the phylogenetic analysis. It will change tree topology and support not only by adding new terminal taxa and characters, but will also modify the alignment itself – most used in this comparison multiple alignment tools are unable to align new sequences to reference alignment without modifying it, so reference sequences are likely to be re-aligned relative to each other too. Therefore, it is impossible to compare "original" and "new" results directly, since it is not known how much change is introduced by new data (new taxa, new characters) and how much by re-arranging old data (re-alignment of reference sequences). Despite all possible effects that adding new sequences can have on the results of phylogenetic analysis, it is obvious that "best case" scenario performed better in assigning new sequences to family-level taxonomic categories (Table 6).

### Paraphyletic clades

Several important and diverse families of marine nematodes are always resolved as paraphyletic in present analysis. Examples include family Monhysteridae (including Xyalidae and Sphaerolaimidae as ingroup clades), Desmodoridae (including Draconematidae as ingroup clade) and Oncholaimidae (including Enchelidiidae as ingroup clade). At least one of them (Desmodoridae) is similarly resolved in large scale phylogenetic studies that use nearly full-length 18S and partial 28S rRNA sequences (Leduc and Zhao 2016, van Megen et al. 2009). OTUs placed within such paraphyletic clades by the tree-based approach can still be assigned taxonomic identification if they fulfil certain criteria. It can be demonstrated by using *Tridentulus* sp. (AJ966507) as an example (Fig. 3). If the OTU is placed within the paraphyletic but highly supported clade (100% bootstrap support for a clade that includes Monhysteridae, Sphaerolaimidae and Xyalidae), outside of the monophyletic ingroup clades (in this case Sphaerolaimidae and Xyalidae) and in the monophyletic and highly supported subclade with identified taxa (other Monhysteridae, genus *Monhystera* in this case), it can be assigned the taxonomic identity of the paraphyletic clade (family Monhysteridae) with confidence.

### Polyphyletic clades

Often polyphyletic clades are caused by insufficient phylogenetic signal of the relatively short (barcode-size) marker. Several examples discussed in sections 1.1 and 1.2 of the Results confirm that erroneous sequences are another important culprit, affecting both resolution and support of clades. In both cases, affected clades are unlikely to be useful for the identification of anonymous barcodes that are placed within them. Polyphyly of families can also reflect genuine divergent history of the phylogenetic marker (barcoding region) that is not followed in current classification or not supported by alternative phylogenies (based on full-length gene or multiple genes). In such cases, anonymous barcodes could still be assigned to one of the subclades and classified within the family, as long as their placement in such subclades is well supported, subclades are well represented with reference taxa and have sufficient bootstrap support.

## Conclusions

1. A number of reference sequences were found in this analysis to be "misplaced" in the phylogenetic trees, suggesting that they are likely incorrectly identified or have sequencing errors. Public sequence databases do include erroneous sequences that will affect the results. Therefore, it is necessary to raise awareness about the importance of quality control of reference datasets for erroneous and incomplete sequences, as both will have negative impact on the results of taxonomy-assignment procedures.
2. The choice of alignment and phylogeny inference algorithms will affect the results. Moreover, alignment may have bigger impact on the topology of the final tree than either one of the phylogeny inference algorithms used in this study (Neighbor joining *versus* Maximum likelihood). It is thus recommended to use more then one combination of both alignment and phylogeny inference algorithms in order to be able to reliably identify anonymous sequences.
3. It is important to understand that trees built using short barcode-size sequences of 18S rRNA will never correspond to the trees based on the full length of the gene and complex alignment and phylogeny inference models. Therefore, some taxa (families, orders) that are monophyletic in the "full-length 18S rRNA" tree may not be monophyletic in the barcode-based tree. Nonetheless, it is still possible to assign taxonomic placement to anonymous OTUs that fall within paraphyletic and polyphyletic clades in the barcode-based tree, depending on their topology and bootstrap support.
4. There were a number of families in our analysis that were represented only by two closely related species and were usually resolved as monophyletic. In such cases, it is difficult to forsee if they will cluster with unidentified OTUs in real-life analyses. Sequencing of more reference taxa from such families should be of higher priority than sequencing of taxa from families that are well represented in reference databases.

## Supplementary Material

Supplementary material 1 Table S1. GenBank accession numbers and classification of sequences used in present analysis Data type: list
File: oo_96341.pdf Holovachov

Supplementary material 2 Neighbor joining tree inferred using Clustal-O alignment of the complete dataset Data type: phylogenetic
File: oo_85395.jpg Holovachov

Supplementary material 3 Neighbor joining tree inferred using Clustal-W alignment of the complete dataset Data type: phylogenetic
File: oo_85396.jpg Holovachov

Supplementary material 4 Neighbor joining tree inferred using MAFFT alignment of the complete dataset Data type: phylogenetic
File: oo_85397.jpg Holovachov

Supplementary material 5 Neighbor joining tree inferred using MUSCLE alignment of the complete dataset Data type: phylogenetic
File: oo_85398.jpg Holovachov

Supplementary material 6 Neighbor joining tree inferred using PRANK alignment of the complete dataset Data type: phylogenetic
File: oo_85399.jpg Holovachov

Supplementary material 7 Neighbor joining tree inferred using SILVA-based alignment of the complete dataset Data type: phylogenetic
File: oo_85400.jpg Holovachov

Supplementary material 8 Table S2. Resolution and bootstrap support (for monophyletic clades) of nematode families based on Neighbor joining analyses of different multiple sequence alignments of complete dataset (POL - polyphyletic, PAR - paraphyletic) Data type: list
File: oo_95694.pdf Holovachov

Supplementary material 9 Maximum likelihood tree inferred using Clustal-O alignment of the complete dataset Data type: phylogenetic
File: oo_85402.jpg Holovachov

Supplementary material 10 Maximum likelihood tree inferred using Clustal-W alignment of the complete dataset Data type: phylogenetic
File: oo_85403.jpg Holovachov

Supplementary material 11 Maximum likelihood tree inferred using MAFFT alignment of the complete dataset Data type: phylogenetic
File: oo_85404.jpg Holovachov

Supplementary material 12 Maximum likelihood tree inferred using MUSCLE alignment of the complete dataset Data type: phylogenetic
File: oo_85405.jpg Holovachov

Supplementary material 13 Maximum likelihood tree inferred using PRANK alignment of the complete dataset Data type: phylogenetic
File: oo_85406.jpg Holovachov

Supplementary material 14 Maximum likelihood tree inferred using SILVA-based alignment of the complete dataset Data type: phylogenetic
File: oo_85407.jpg Holovachov

Supplementary material 15 Table S3. Resolution and bootstrap support (for monophyletic clades) of nematode families based on Maximum likelihood analyses of different multiple sequence alignments of complete dataset (POL - polyphyletic, PAR - paraphyletic) Data type: list
File: oo_95695.pdf Holovachov

Supplementary material 16 Neighbor joining tree inferred using Clustal-O alignment of the "filtered" dataset Data type: phylogenetic
File: oo_85409.jpg Holovachov

Supplementary material 17 Neighbor joining tree inferred using Clustal-W alignment of the "filtered" dataset Data type: phylogenetic
File: oo_85410.jpg Holovachov

Supplementary material 18 Neighbor joining tree inferred using MAFFT alignment of the "filtered" dataset Data type: phylogenetic
File: oo_85411.jpg Holovachov

Supplementary material 19 Neighbor joining tree inferred using MUSCLE alignment of the "filtered" dataset Data type: phylogenetic
File: oo_85412.jpg Holovachov

Supplementary material 20 Neighbor joining tree inferred using PRANK alignment of the "filtered" dataset Data type: phylogenetic
File: oo_85413.jpg Holovachov

Supplementary material 21 Neighbor joining tree inferred using SILVA-based alignment of the "filtered" dataset Data type: phylogenetic
File: oo_85414.jpg Holovachov

Supplementary material 22 Table S4. Resolution and bootstrap support (for monophyletic clades) of nematode families based on Neighbor joining analyses of different multiple sequence alignments of "filtered" dataset (POL - polyphyletic, PAR - paraphyletic) Data type: list
File: oo_96343.pdf Holovachov

Supplementary material 23 Maximum likelihood tree inferred using Clustal-O alignment of the "filtered" dataset Data type: phylogenetic
File: oo_85420.jpg Holovachov

Supplementary material 24 Maximum likelihood tree inferred using Clustal-W alignment of the "filtered" dataset Data type: phylogenetic
File: oo_85421.jpg Holovachov

Supplementary material 25 Maximum likelihood tree inferred using MAFFT alignment of the "filtered" dataset Data type: phylogenetic
File: oo_85422.jpg Holovachov

Supplementary material 26 Maximum likelihood tree inferred using MUSCLE alignment of the "filtered" dataset Data type: phylogenetic
File: oo_85423.jpg Holovachov

Supplementary material 27 Maximum likelihood tree inferred using PRANK alignment of the "filtered" dataset Data type: phylogenetic
File: oo_85424.jpg Holovachov

Supplementary material 28 Maximum likelihood tree inferred using SILVA-based alignment of the "filtered" dataset Data type: phylogenetic
File: oo_85425.jpg Holovachov

Supplementary material 29 Table S5. Resolution and bootstrap support (for monophyletic clades) of nematode families based on Maximum likelihood analyses of different multiple sequence alignments of "filtered" dataset (POL - polyphyletic, PAR - paraphyletic) Data type: list
File: oo_96345.pdf Holovachov

Supplementary material 30 Neighbor joining tree inferred using Clustal-O alignment of the "long" dataset Data type: phylogenetic
File: oo_86262.jpg Holovachov

Supplementary material 31 Neighbor joining tree inferred using Clustal-W alignment of the "long" dataset Data type: phylogenetic
File: oo_85428.jpg Holovachov

Supplementary material 32 Neighbor joining tree inferred using MAFFT alignment of the "long" dataset Data type: phylogenetic
File: oo_85429.jpg Holovachov

Supplementary material 33 Neighbor joining tree inferred using MUSCLE alignment of the "long" dataset Data type: phylogenetic
File: oo_85430.jpg Holovachov

Supplementary material 34 Neighbor joining tree inferred using PRANK alignment of the "long" dataset Data type: phylogenetic
File: oo_85431.jpg Holovachov

Supplementary material 35 Neighbor joining tree inferred using SILVA-based alignment of the "long" dataset Data type: phylogenetic
File: oo_85432.jpg Holovachov

Supplementary material 36 Table S6. Resolution and bootstrap support (for monophyletic clades) of nematode families based on Neighbor joining analyses of different multiple sequence alignments of "long" dataset (POL - polyphyletic, PAR - paraphyletic) Data type: list
File: oo_95718.pdf Holovachov

Supplementary material 37 Maximum likelihood tree inferred using Clustal-O alignment of the "long" dataset Data type: phylogenetic
File: oo_86264.jpg Holovachov

Supplementary material 38 Maximum likelihood tree inferred using Clustal-W alignment of the "long" dataset Data type: phylogenetic
File: oo_85435.jpg Holovachov

Supplementary material 39 Maximum likelihood tree inferred using MAFFT alignment of the "long" dataset Data type: phylogenetic
File: oo_85436.jpg Holovachov

Supplementary material 40 Maximum likelihood tree inferred using MUSCLE alignment of the "long" dataset Data type: phylogenetic
File: oo_85437.jpg Holovachov

Supplementary material 41 Maximum likelihood tree inferred using PRANK alignment of the "long" dataset Data type: phylogenetic
File: oo_85438.jpg Holovachov

Supplementary material 42 Maximum likelihood tree inferred using SILVA-based alignment of the "long" dataset Data type: phylogenetic
File: oo_85439.jpg Holovachov

Supplementary material 43 Table S7. Resolution and bootstrap support (for monophyletic clades) of nematode families based on Maximum likelihood analyses of different multiple sequence alignments of "long" dataset (POL - polyphyletic, PAR - paraphyletic) Data type: list
File: oo_96346.pdf Holovachov

## Figures and Tables

**Table 1.** Number of nematode families resolved as monophyletic and with high (≥70%) bootstrap support for all combinations of sequence dataset, alignment and phylogeny inference algorithms.

**Dataset** **(number of families)**	**Phylogeny inference**	**Alignment**					
		**Clustal-O**	**Clustal-W**	**MAFFT**	**MUSCLE**	**PRANK**	**SILVA**
Complete (50 families)	Neighbor joining	22	24	24	23	24	23
Complete (50 families)	Maximum likelihood	21	24	24	21	26	21
"Filtered" (50 families)	Neighbor joining	27	29	29	30	29	29
"Filtered" (50 families)	Maximum likelihood	28	32	28	28	31	26
"Long" (48 families)	Neighbor joining	32	34	34	33	35	32
"Long" (48 families)	Maximum likelihood	29	33	32	30	36	30

**Table 2.** Comparison of changes in bootstrap support (increase or decrease) and resolution for different nematode families between Neighbor joining analyses of complete and "filtered" datasets. Legend: "M" – clade changed from paraphyletic or polyphyletic to monophyletic; "P" – clade changed from monophyletic to paraphyletic or polyphyletic; "–" – clade remained paraphyletic or polyphyletic; "R" – monophyletic clade became fully resolved (bootstrap increased to ≥70%); "U" – monophyletic clade became unresolved (bootstrap decreased to <70%).

**Taxon (family** or *superfamily)	**Clustal-O**	**Clustal-W**	**MAFFT**	**MUSCLE**	**PRANK**	**SILVA**
Rhabditidae	0	0	+1	0	0	0
Plectidae	-12	+1	-2	-5	+1	-3
Aphanolaimidae	+8	-1	-6	-2	-2	0
Axonolaimidae	–	+57 M	+47 M	+42 M	–	–
Comesomatidae	+2	+1	+3	-7	+5	-1
Xyalidae	+13	–	+3	+56 R	-18	+2
Siphonolaimidae	+4	0	0	+1	0	0
Ceramonematidae	-5 U	-3	+5	+10	+2	+2
Desmoscolecidae	+3	+3	+2	+8	-9	+1
Draconematidae	-5	+3	+3	+4 R	+8	-3
Monoposthiidae	+100 MR	+100 MR	+100 MR	+100 MR	+100 MR	+100 MR
Selachinematidae	+32 M	+36 M	+44 M	+33 M	+19 M	+26 M
Cyatholaimidae	+93 MR	+98 MR	+90 MR	+99 MR	+96 MR	+95 MR
Chromadoridae	+31 R	+1	+3	-8	+2	+5
Mononchoidea*	0	0	-1	0	0	0
Prismatolaimidae	+1	+2	+1	+1	-2	+2
Tobrilidae	+48	+64	-7	-16	+2	+41 R
Enoplidae	+100 MR	+100 MR	+100 MR	+100 MR	+100 MR	+100 MR
Thoracostomopsidae	+93 MR	+95 MR	+97 MR	+94 MR	+97 MR	+96 MR
Phanodermatidae	+28 M	+4	–	–	–	–
Anticomidae	+100 MR	+100 MR	+100 MR	+100 MR	+100 MR	+100 MR
Leptosomatidae	0	0	0	+1	0	-1
Alaimidae	+12	+4	0	+2	+3	0

**Table 3.** Comparison of changes in bootstrap support (increase or decrease) and resolution for different nematode families between Maximum likelihood analyses of complete and "filtered" datasets. Legend: "M" – clade changed from paraphyletic or polyphyletic to monophyletic; "P" – clade changed from monophyletic to paraphyletic or polyphyletic; "–" – clade remained paraphyletic or polyphyletic; "R" – monophyletic clade became fully resolved (bootstrap increased to ≥70%); "U" – monophyletic clade became unresolved (bootstrap decreased to <70%).

**Taxon (family** or *superfamily)	**Clustal-O**	**Clustal-W**	**MAFFT**	**MUSCLE**	**PRANK**	**SILVA**
Anguinidae	-1	+1	0	0	0	0
Rhabditidae	0	+1	-9	-5	-5	0
Teratocephalidae	-1	+1	+1	+1	0	+4
Plectidae	-9	+3	-14 U	-7	–	+2
Aphanolaimidae	+11	+4	-7 U	+78 MR	+3	+1
Camacolaimidae	+22 R	+71 MR	-2	-6	+42 M	-2
Axonolaimidae	+47 M	+64 M	–	+43 M	+30 M	+38 M
Comesomatidae	-14	+15	0	-1	+1	0
Xyalidae	+3	+1	+1	-1	-6	-1
Siphonolaimidae	+6	+1	0	0	+3	0
Ceramonematidae	-2	+4	0	+18 R	+10	–
Desmoscolecidae	0	+1	-1	+1	-3	+2
Draconematidae	–	–	+36 M	-5	+2	0
Monoposthiidae	+100 MR	+100 MR	+100 MR	+100 MR	+100 MR	+100 MR
Selachinematidae	+24 M	+70 M R	+67 M	+47 M	+49 M	–
Achromadoridae	+4	-1	-7	+5	+6	0
Cyatholaimidae	+88 MR	+98 MR	+94 MR	+91 MR	+96 MR	+95 MR
Chromadoridae	+32 R	+82 MR	+95 MR	+26	+6	+29 R
Haliplectidae	0	0	0	0	0	+1
Dorylaimoidea*	0	0	0	0	0	-1
Mononchoidea*	+22	-4	0	0	-9	-2 U
Bathyodontidae	+2	-5	+1	+1	-11	+1
Cryptonchidae	0	+1	+1	-1	0	0
Mermithidae	0	+4	+1	-1	+9 R	+1
Prismatolaimidae	-2	+2	-1	+1	-1	+1
Tripylidae	0	0	-1	0	-1	0
Tobrilidae	+31	0	-52 P	–	0	-1
Enchelidiidae	-2	+2	+55 M	-45 P	-4	+3
Enoplidae	+29	+35 R	+40 R	+32 R	+28	+58 R
Thoracostomopsidae	+93 MR	+97 MR	+83 MR	+90 MR	+97 MR	+91 MR
Phanodermatidae	–	+5	–	+39 M	–	–
Anticomidae	+100 MR	+100 MR	+100 MR	+100 MR	+100 MR	+100 MR
Leptosomatidae	-2	3	-5	+11	+1	+1
Trefusiidae	–	–	–	–	+10 M	–
Alaimidae	+10	+2	-1	-2	-3	+1
Rhabdolaimidae	+2	0	-1	-2	+3	+1

**Table 4.** Comparison of changes in bootstrap support (increase or decrease) and resolution for different nematode families between Neighbor joining analyses of "filtered" and "long" datasets. Legend: "M" – clade changed from paraphyletic or polyphyletic to monophyletic; "P" – clade changed from monophyletic to paraphyletic or polyphyletic; "–" – clade remained paraphyletic or polyphyletic; "R" – monophyletic clade became fully resolved (bootstrap increased to ≥70%); "U" – monophyletic clade became unresolved (bootstrap decreased to <70%).

**Taxon (family** or *superfamily)	**Clustal-O**	**Clustal-W**	**MAFFT**	**MUSCLE**	**PRANK**	**SILVA**
Anguinidae	0	0	0	0	0	+1
Plectidae	+1	+1	-9	0	-3	0
Chronogastridae	+38 M	+23 M	+26 M	+34 M	+41 M	+57 M
Aphanolaimidae	+9 R	+2	+6	+15	+10	+5
Camacolaimidae	+82 MR	+80 MR	+65 MR	+67 MR	+77 MR	+69 MR
Axonolaimidae	+76 MR	+28 R	+32 R	+11	+78 MR	+77 MR
Comesomatidae	-5	+1	-2	+11	+2	+1
Xyalidae	+4	+44 M	+6 R	-12	+48 R	+29
Siphonolaimidae	+2	0	0	-4	0	0
Ceramonematidae	-15	+5	+4	+22 R	+16	0
Desmoscolecidae	+6	-14	-1	-16	+1	-2
Draconematidae	+20	+25 R	+56 R	+1	+23 R	+20
Microlaimidae	–	–	–	+19 M	–	–
Selachinematidae	+27	+29	+20	-7	+33	+1
Cyatholaimidae	-3	0	+5	-2	-6	-5
Chromadoridae	+8	+8	-1	-7	+8	+8
Mononchoidea*	+1	-1	+1	+1	0	+1
Prismatolaimidae	-6	-2	-4	-1	0	-1
Tobrilidae	-7	-10	+21	+22	+4	+9
Enchelidiidae	+99 MR	+98 MR	+97 MR	+98 MR	+99 MR	+96 MR
Thoracostomopsidae	+7	+5	+3	+6	+3	+4
Leptosomatidae	0	0	-1	0	0	0
Trefusiidae	+93 MR	+99 MR	+87 MR	+91 MR	+79 MR	+94 MR
Anoplostomatidae	+79 MR	+99 MR	+98 MR	+97 MR	+99 MR	+98 MR
Alaimidae	0	-3	-5	+1	-2	-1

**Table 5.** Comparison of changes in bootstrap support (increase or decrease) and resolution for different nematode families between Maximum likelihood analyses of "filtered" and "long" datasets. Legend: "M" – clade changed from paraphyletic or polyphyletic to monophyletic; "P" – clade changed from monophyletic to paraphyletic or polyphyletic; "–" – clade remained paraphyletic or polyphyletic; "R" – monophyletic clade became fully resolved (bootstrap increased to ≥70%); "U" – monophyletic clade became unresolved (bootstrap decreased to <70%).

**Taxon (family** or *superfamily)	**Clustal-O**	**Clustal-W**	**MAFFT**	**MUSCLE**	**PRANK**	**SILVA**
Anguinidae	+2	0	+1	0	0	0
Rhabditidae	0	0	+20	-2	+6	0
Teratocephalidae	0	0	-4	-2	+2	+1
Plectidae	+13	+2	-56 P	-45 P	+45 M	+2
Chronogastridae	–	–	–	–	+49 M	+41 M
Aphanolaimidae	+1	-80 P	+13 R	+2	-1	-3
Camacolaimidae	+4	+6	+29	+41 R	+31 R	+12
Axonolaimidae	-47 P	+10 R	+85 MR	+23	+43 R	+33 R
Comesomatidae	+10	-3	-2	-4	-3	-1
Xyalidae	0	+3	-1	+9	+16	+9
Siphonolaimidae	+4	-1	-2	-1	0	0
Ceramonematidae	-19	-1	-13	+15	+17	+50 M
Desmoscolecidae	0	-3	+1	-1	-2	0
Draconematidae	–	+65 M	+37 R	+21	+35 R	+8
Microlaimidae	–	+14	+9	+14	–	–
Selachinematidae	+45	-1 U	-4	-47 P	+22 R	+100 MR
Ethmolaimidae	0	0	0	-1	0	0
Achromadoridae	+1	+2	+4	-5	0	-2
Cyatholaimidae	-5	-20	-14	-8	-21	-18
Chromadoridae	+1	+16	+3	0	+4	+3
Dorylaimoidea*	-8	-5	-4	-2	-1	-3
Mononchoidea*	-3	+2	+3	+15	+9	+4 R
Bathyodontidae	+2	+1	+7	-2	+10	0
Cryptonchidae	0	0	-1	0	0	0
Mermithidae	-2	0	+4	-7	-3	-5
Prismatolaimidae	0	-2	+1	-4	-2	-2
Tripylidae	0	0	+2	0	+1	0
Tobrilidae	-9	+1	+60 M	+53 M	-6	+7 R
Enchelidiidae	+34 R	+37 R	+44 R	+99 MR	+43 R	+35 R
Thoracostomopsidae	+7	+3	+17	+10	+3	+9
Leptosomatidae	+34 R	-9	+16 R	+12	-5	0
Trefusiidae	+52 M	+80 MR	–	–	+30	–
Anoplostomatidae	–	+95 MR	+89 MR	+87 MR	+93 MR	+82 MR
Alaimidae	-8	-10	-22 U	-9	-7	-20 U
Rhabdolaimidae	0	-7	0	-1	0	0

**Table 6.** GenBank accession numbers and classification of sequences used in the final comparison of "worst case" and "best case" scenarios, and their identification outcomes. * denotes taxa placed in monophyletic clade but with low bootstrap support.

**Acc. number**	**Family**	**Genus**	**Species**	"**worst-case**" **scenario**	"**best case**" **scenario**
FJ969132	Camacolaimidae	* Deontolaimus *	sp.	identified*	identified
AY854235	Comesomatidae	* Sabatieria *	*punctata*	identified	identified
JN968250	Comesomatidae	* Sabatieria *	*pulchra*	identified	identified
JN968228	Comesomatidae	* Sabatieria *	*pulchra*	identified	identified
JN968221	Comesomatidae	* Sabatieria *	sp.	identified	identified
JN968273	Comesomatidae	* Setosabatieria *	*hilarula*	identified	identified
JN968231	Xyalidae	* Theristus *	sp.	identified	identified
JN968217	Xyalidae	* Daptonema *	sp.	identified	identified
JN968233	Xyalidae	* Daptonema *	*setosum*	identified	identified
JN968218	Xyalidae	* Metadesmolaimus *	sp.	identified	identified
AJ966507	Monhysteridae	* Tridentulus *	sp.	unidentified	unidentified
Y16920	Desmodoridae	* Leptonemella *	sp.	unidentified	unidentified
KF453618	Desmodoridae	* Eubostrichus *	sp.	unidentified	unidentified
JN968220	Cyatholaimidae	* Paracyatholaimus *	*intermedius*	unidentified	identified
FJ969133	Cyatholaimidae	* Paracyatholaimus *	*intermedius*	unidentified	identified
JN968215	Chromadoridae	* Neochromadora *	sp.	identified*	identified
JN968255	Chromadoridae	* Neochromadora *	sp.	identified*	identified
JN968230	Chromadoridae	* Neochromadora *	sp.	identified*	identified
JN968246	Chromadoridae	* Neochromadora *	sp.	identified*	identified
JN968267	Chromadoridae	* Neochromadora *	sp.	identified*	identified
JN968222	Chromadoridae	* Dichromadora *	sp.	identified*	identified
JN968224	Chromadoridae	* Chromadorita *	*tentabundum*	identified*	identified
JN968283	Chromadoridae	* Punctodora *	*ratzeburgensis*	identified*	identified
AY854194	Anoplostomatidae	* Anoplostoma *	sp.	unidentified	identified
JN968238	Thoracostomopsidae	* Enoplolaimus *	sp.	unidentified	identified
