# Supplementary material for: Metabarcoding of marine nematodes – evaluation of reference datasets used in tree-based taxonomy assignment approach
Source: Biodivers Data J. 2016 Sep 21;(4):e10021. doi: 10.3897/BDJ.4.e10021 (PMC5136706; doi:10.3897/BDJ.4.e10021)
Supplement: Supplementary material 1 — Table S1. GenBank accession numbers and classification of sequences used in present analysis [file biodiversity_data_journal-4-e10021-s001.pdf]

File name: S01-TABS1-TAXA.pdf

Table S1. GenBank accession numbers and classification of sequences used in present analysis.

Acc. number	Family or *superfamily	Genus	Species	complete dataset	"filtered" dataset	"long" dataset
AF202164	Anguinidae	*Subanguina*	*radicicola*	+	+	+
EU669912	Anguinidae	*Halenchus*	*fucicola*	+	+	+
JQ429768	Anguinidae	*Ditylenchus*	*drepanocercus*	+	+	+
KJ636296	Anguinidae	*Ditylenchus*	*dipsaci*	+	+	+
AF083020	Rhabditidae	*Pellioditis*	*mediterranea*	+	+	+
AF083021	Rhabditidae	*Pellioditis*	*marina*	+	+	+
U13933	Rhabditidae	*Pellioditis*	*typica*	+	+	+
AF036607	Teratocephalidae	*Teratocephalus*	*lirellus*	+	+	+
AY284683	Teratocephalidae	*Teratocephalus*	*terrestris*	+	+	+
AF036602	Plectidae	*Plectus*	*aquatilis*	+	+	+
AY593928	Plectidae	*Plectus*	*rhizophilus*	+	+	+
AY593930	Plectidae	*Plectus*	*cirratus*	+	+	+
AY284697	Plectidae	*Anaplectus*	*grandepapillatus*	+	+	+
JX905207	Chronogastridae	*Cynura*	*klunderi*	+	+	–
AY593931	Chronogastridae	*Kischkenema*	*boettgeri*	+	+	+
FJ040455	Chronogastridae	*Chronogaster*	sp.	+	+	+
FJ040456	Chronogastridae	*Chronogaster*	*typica*	+	+	+
KJ636361	Chronogastridae	*Chronogaster*	sp.	+	+	+
AY593933	Aphanolaimidae	*Aphanolaimus*	*aquaticus*	+	+	+
AY593932	Aphanolaimidae	*Aphanolaimus*	*aquaticus*	+	+	+
EF591319	Aphanolaimidae	*Aphanonchus*	cf. *europaeus*	+	+	+
KJ636380	Aphanolaimidae	*Paraphanolaimus*	*behningi*	+	+	+
EF591323	Leptolaimidae	*Leptolaimus*	sp.	+	+	+
FJ040458	Leptolaimidae	*Leptolaimus*	sp.	+	+	+
EF591324	Leptolaimidae	*Leptolaimus*	sp.	+	+	+
EF591320	Leptolaimidae	*Paraplectonema*	*pedunculatum*	+	+	+
JX678597	Camacolaimidae	*Alaimella*	sp.	+	+	+
EF591321	Camacolaimidae	*Setostephanolaimus*	*spartinae*	+	+	+
FJ040457	Camacolaimidae	*Deontolaimus*	*papillatus*	+	+	+
JX678599	Camacolaimidae	*Deontolaimus*	sp.	+	+	+
EF591325	Camacolaimidae	*Deontolaimus*	sp.	+	+	+
JX678598	Camacolaimidae	*Deontolaimus*	sp.	+	+	+
EF591326	Camacolaimidae	*Procamacolaimus*	sp.	+	+	+
FJ969115	Camacolaimidae	*Anguinoides*	sp.	+	+	+
JX678601	Camacolaimidae	*Onchium*	sp.	+	+	+

Acc. number	Family or *superfamily	Genus	Species	complete dataset	"filtered" dataset	"long" dataset
EF591328	Camacolaimidae	*Onchium*	sp.	+	+	+
AY854231	Axonolaimidae	*Ascolaimus*	*elongatus*	+	+	+
EF591330	Axonolaimidae	*Ascolaimus*	cf. *elongatus*	+	+	+
AY854232	Axonolaimidae	*Axonolaimus*	*helgolandicus*	+	+	+
EF591331	Axonolaimidae	*Axonolaimus*	sp.	+	+	+
FJ040461	Axonolaimidae	*Axonolaimus*	sp.	+	+	+
AM234630	Axonolaimidae	*Parodontophora*	sp.	+	–	–
AY854233	Axonolaimidae	*Odontophora*	*rectangula*	+	+	–
KF591737	Axonolaimidae	*Odontophora*	cf. *peritricha*	+	+	–
EF591329	Diplopeltidae	*Diplopeltula*	sp.	+	+	+
AY593939	Diplopeltidae	*Cylindrolaimus*	*communis*	+	+	+
AY854234	Comesomatidae	*Sabatieria*	*celtica*	+	+	+
AY854239	Comesomatidae	*Sabatieria*	sp.	+	+	+
FJ040466	Comesomatidae	*Sabatieria*	*pulchra*	+	+	+
AY854236	Comesomatidae	*Sabatieria*	*punctata*	+	+	+
AY854240	Comesomatidae	*Setosabatieria*	*hilarula*	+	+	+
AM234047	Comesomatidae	*Dorylaimopsis*	*punctata*	+	+	–
FJ969130	Monhysteridae	*Monhystera*	*paludicola*	+	+	+
AY593938	Monhysteridae	*Monhystera*	*riemanni*	+	+	+
KJ636259	Monhysteridae	*Monhystera*	*stagnalis*	+	+	+
KJ636250	Monhysteridae	*Eumonhystera*	cf. *vulgaris*	+	+	+
AY593937	Monhysteridae	*Eumonhystera*	*filiformis*	+	+	+
KJ636238	Monhysteridae	*Eumonhystera*	*filiformis*	+	+	+
EF591334	Monhysteridae	*Geomonhystera*	*villosa*	+	+	+
FJ040465	Monhysteridae	*Geomonhystera*	sp.	+	+	+
AJ966485	Monhysteridae	*Halomonhystera*	*disjuncta*	+	+	+
HF572952	Monhysteridae	*Halomonhystera*	sp.	+	+	+
AF036611	Monhysteridae	*Diplolaimelloides*	*meyli*	+	+	+
AJ966482	Monhysteridae	*Diplolaimella*	*dievengatensis*	+	+	+
AY854225	Xyalidae	*Daptonema*	*oxycerca*	+	+	+
AF047889	Xyalidae	*Daptonema*	*procerus*	+	+	+
AY854223	Xyalidae	*Daptonema*	*hirsutum*	+	+	+
AY854226	Xyalidae	*Daptonema*	*setosum*	+	+	+
AY854224	Xyalidae	*Daptonema*	*normandicum*	+	+	+
AY284695	Xyalidae	*Theristus*	*agilis*	+	+	+
AJ966505	Xyalidae	*Theristus*	*acer*	+	+	+
AJ966491	Xyalidae	*Metadesmolaimus*	sp.	+	+	+
LK054719	Xyalidae	*Steineria*	*sterreri*	+	+	–
KC920423	Xyalidae	*Zygonemella*	*striata*	+	+	+

Acc. number	Family or *superfamily	Genus	Species	complete dataset	"filtered" dataset	"long" dataset
AY854228	Sphaerolaimidae	*Sphaerolaimus*	*hirsutus*	+	+	+
JN968239	Sphaerolaimidae	*Sphaerolaimus*	*hirsutus*	+	+	+
AY854230	Linhomoeidae	*Terschellingia*	*longicaudata*	+	+	+
JN968242	Linhomoeidae	*Terschellingia*	*longicaudata*	+	+	+
EF591333	Linhomoeidae	*Desmolaimus*	sp.	+	+	+
AY854229	Linhomoeidae	*Desmolaimus*	*zeelandicus*	+	+	+
EF591332	Linhomoeidae	*Desmolaimus*	sp.	+	+	+
AM235216	Linhomoeidae	*Paralinhomoeus*	sp.	+	+	–
DQ394777	Linhomoeidae	*Paralinhomeus*	sp.	+	+	–
DQ408760	Siphonolaimidae	*Astomonema*	sp.	+	+	+
DQ408761	Siphonolaimidae	*Astomonema*	sp.	+	+	+
FJ474095	Ceramonematidae	*Ceramonema*	*reticulatum*	+	+	–
JN815321	Ceramonematidae	*Ceramonema*	*reticulatum*	+	+	+
JN815320	Ceramonematidae	*Ceramonema*	*altogolfi*	+	+	+
JN815319	Ceramonematidae	*Ceramonema*	*inguinispina*	+	+	+
JN815322	Desmoscolecidae	*Desmoscolex*	sp.	+	+	+
EF591342	Desmoscolecidae	*Desmoscolex*	sp.	+	+	+
FJ182217	Draconematidae	*Draconema*	*japonicum*	+	+	+
FJ182220	Draconematidae	*Paradraconema*	*jejuense*	+	+	+
FJ182221	Draconematidae	*Paradraconema*	sp.	+	+	+
FJ182222	Draconematidae	*Prochaetosoma*	sp.	+	+	+
FJ182216	Draconematidae	*Dracograllus*	sp.	+	+	+
AF047891	Desmodoridae	*Chromadoropsis*	*vivipara*	+	+	+
AY854216	Desmodoridae	*Metachromadora*	*remanei*	+	+	+
FJ040469	Desmodoridae	*Metachromadora*	sp.	+	+	+
Y16913	Desmodoridae	*Desmodora*	*ovigera*	+	+	+
AM234628	Desmodoridae	*Desmodora*	*pontica*	+	+	–
AY854215	Desmodoridae	*Desmodora*	*communis*	+	+	+
AY854217	Desmodoridae	*Spirinia*	*parasitifera*	+	+	+
JN968216	Desmodoridae	*Spirinia*	*parasitifera*	+	+	–
Y16923	Desmodoridae	*Xyzzors*	sp.	+	+	+
LK054708	Desmodoridae	*Zalonema*	sp.	+	+	–
Y16912	Desmodoridae	*Catanema*	sp.	+	+	+
Y16917	Desmodoridae	*Eubostrichus*	*topiarius*	+	+	+
Y16915	Desmodoridae	*Eubostrichus*	*dianeae*	+	+	+
Y16916	Desmodoridae	*Eubostrichus*	*parasitiferus*	+	+	+
KJ414468	Desmodoridae	*Leptonemella*	*vicina*	+	+	+
Y16918	Desmodoridae	*Laxus*	*cosmopolitus*	+	+	+
Y16919	Desmodoridae	*Laxus*	*oneistus*	+	+	+

Acc. number	Family or *superfamily	Genus	Species	complete dataset	"filtered" dataset	"long" dataset
KJ414465	Desmodoridae	*Robbea*	*ruetzleri*	+	+	–
KJ414466	Desmodoridae	*Robbea*	*hypermnestra*	+	+	–
Y16921	Desmodoridae	*Robbea*	*hypermnestra*	+	+	+
Y16922	Desmodoridae	*Stilbonema*	*majum*	+	+	+
AY854218	Microlaimidae	*Calomicrolaimus*	*parahonestus*	+	+	+
AY854219	Microlaimidae	*Calomicrolaimus*	sp.	+	+	+
AY854220	Microlaimidae	*Molgolaimus*	*demani*	+	+	+
AY284722	Microlaimidae	*Prodesmodora*	*circulata*	+	+	+
FJ040476	Microlaimidae	*Prodesmodora*	sp.	+	+	+
AY854222	Monoposthiidae	*Nudora*	*bipapillata*	+	+	+
JQ071928	Monoposthiidae	*Nudora*	*ilhabelae*	+	+	+
AY854221	Monoposthiidae	*Monoposthia*	*costata*	+	–	–
FJ040505	Monoposthiidae	*Monoposthia*	sp.	+	+	+
AM234629	Selachinematidae	*Halichoanolaimus*	*dolichurus*	+	+	–
EF591338	Selachinematidae	*Halichoanolaimus*	sp.	+	+	+
FJ040468	Selachinematidae	*Synonchiella*	sp.	+	+	+
KF591723	Selachinematidae	*Gammanema*	sp.	+	–	–
AY284714	Selachinematidae	*Choanolaimus*	*psammophilus*	+	+	+
AY284715	Selachinematidae	*Choanolaimus*	*psammophilus*	+	+	+
AY593942	Ethmolaimidae	*Ethmolaimus*	*pratensis*	+	+	+
FJ040475	Ethmolaimidae	*Ethmolaimus*	*pratensis*	+	+	+
AY593941	Achromadoridae	*Achromadora*	*ruricola*	+	+	+
AY593940	Achromadoridae	*Achromadora*	cf. *terricola*	+	+	+
AY854213	Cyatholaimidae	*Cyatholaimus*	sp.	+	+	+
JN968214	Cyatholaimidae	*Cyatholaimus*	sp.	+	–	–
AF036612	Cyatholaimidae	*Praeacanthonchus*	sp.	+	+	+
AY854214	Cyatholaimidae	*Praeacanthonchus*	*punctatus*	+	+	+
AJ966495	Cyatholaimidae	*Paracyatholaimus*	*intermedius*	+	+	+
LK054720	Cyatholaimidae	*Longicyatholaimus*	sp.	+	–	–
KF591743	Cyatholaimidae	*Pomponema*	sp.	+	–	–
AY854204	Chromadoridae	*Atrochromadora*	*microlaima*	+	+	+
AY854209	Chromadoridae	*Dichromadora*	sp.	+	+	+
FJ040506	Chromadoridae	*Dichromadora*	sp.	+	+	+
AY854207	Chromadoridae	*Chromadorina*	*germanica*	+	+	+
AY854205	Chromadoridae	*Chromadora*	*nudicapitata*	+	+	+
KJ636220	Chromadoridae	*Chromadorina*	*bioculata*	+	+	+
FJ969119	Chromadoridae	*Chromadorita*	*leuckarti*	+	+	+
AY854208	Chromadoridae	*Chromadorita*	*tentabundum*	+	+	+
AY854206	Chromadoridae	*Chromadora*	sp.	+	+	+

Acc. number	Family or *superfamily	Genus	Species	complete dataset	"filtered" dataset	"long" dataset
EF591341	Chromadoridae	*Prochromadora*	sp.	+	+	+
AY854210	Chromadoridae	*Neochromadora*	sp.	+	+	+
JN968227	Chromadoridae	*Punctodora*	*ratzeburgensis*	+	+	+
AY854211	Chromadoridae	*Spilophorella*	*paradoxa*	+	+	+
JN968257	Chromadoridae	*Ptycholaimellus*	sp.	+	+	–
FJ969123	Haliplectidae	*Haliplectus*	cf. *dorsalis*	+	+	+
JN815323	Haliplectidae	*Haliplectus*	sp.	+	+	+
AY284776	Dorylaimoidea*	*Dorylaimus*	*stagnalis*	+	+	+
AY284777	Dorylaimoidea*	*Dorylaimus*	*stagnalis*	+	+	+
AY284826	Dorylaimoidea*	*Paractinolaimus*	*macrolaimus*	+	+	+
KJ636379	Dorylaimoidea*	*Paractinolaimus*	*macrolaimus*	+	+	+
AJ966493	Mononchoidea*	*Mononchus*	*truncatus*	+	+	+
AY284765	Mononchoidea*	*Mononchus*	*aquaticus*	+	+	+
AJ966474	Mononchoidea*	*Anatonchus*	*tridentatus*	+	+	+
FJ969116	Bathyodontidae	*Bathyodontus*	*mirus*	+	+	+
AY552964	Bathyodontidae	*Bathyodontus*	*cylindricus*	+	+	+
EF207244	Cryptonchidae	*Cryptonchus*	*tristis*	+	+	+
FJ040479	Cryptonchidae	*Cryptonchus*	sp.	+	+	+
JN968229	Mermithidae	*Mermis*	*nigrescens*	+	+	+
AF036641	Mermithidae	*Mermis*	*nigrescens*	+	+	+
FN400892	Mermithidae	*Isomermis*	*lairdi*	+	+	+
AY284729	Prismatolaimidae	*Prismatolaimus*	*intermedius*	+	+	+
AY593957	Prismatolaimidae	*Prismatolaimus*	*dolichurus*	+	+	+
AY284727	Prismatolaimidae	*Prismatolaimus*	cf. *dolichurus*	+	+	+
AY284730	Tripylidae	*Tripyla*	cf. *filicaudata*	+	+	+
KJ636224	Tripylidae	*Tripyla*	*glomerans*	+	+	+
GQ503062	Tripylidae	*Tripyla*	*bioblitz*	+	+	–
AJ966506	Tobrilidae	*Tobrilus*	*gracilis*	+	+	+
KJ636217	Tobrilidae	*Epitobrilus*	*stefanskii*	+	+	+
KJ636226	Tobrilidae	*Eutobrilus*	*grandipapillatus*	+	+	+
KJ636235	Tobrilidae	*Eutobrilus*	*nothus*	+	+	+
KJ636231	Tobrilidae	*Semitobrilus*	*pellucidus*	+	+	+
AF047890	Oncholaimidae	*Pontonema*	*vulgare*	+	+	+
AY854195	Oncholaimidae	*Adoncholaimus*	*fuscus*	+	+	+
AF036642	Oncholaimidae	*Adoncholaimus*	sp.	+	+	+
KF591739	Oncholaimidae	*Oncholaimus*	sp.	+	–	–
AM234625	Oncholaimidae	*Oncholaimus*	sp.	+	+	–
LK054723	Oncholaimidae	*Oncholaimellus*	sp.	+	+	–
AY854198	Oncholaimidae	*Viscosia*	*viscosa*	+	+	+

Acc. number	Family or *superfamily	Genus	Species	complete dataset	"filtered" dataset	"long" dataset
AY854197	Oncholaimidae	*Viscosia*	sp.	+	+	+
FJ040494	Oncholaimidae	*Viscosia*	sp.	+	+	+
GU139748	Oncholaimidae	*Metoncholaimus*	sp.	+	+	–
HM564435	Enchelidiidae	*Pareurystomina*	sp.	+	+	–
HM564491	Enchelidiidae	*Pareurystomina*	sp.	+	+	–
HM564537	Enchelidiidae	*Bathyeurystomina*	sp.	+	+	–
HM564602	Enchelidiidae	*Bathyeurystomina*	sp.	+	+	–
FJ040503	Enchelidiidae	*Calyptronema*	sp.	+	+	+
AY854199	Enchelidiidae	*Calyptronema*	*maxweberi*	+	+	+
FJ040502	Enchelidiidae	*Symplocostoma*	sp.	+	+	+
AY854192	Enoplidae	*Enoplus*	*communis*	+	+	+
U88336	Enoplidae	*Enoplus*	*brevis*	+	+	+
Y16914	Enoplidae	*Enoplus*	*meridionalis*	+	+	+
AY854193	Thoracostomopsidae	*Enoploides*	*brunettii*	+	+	+
FJ040490	Thoracostomopsidae	*Enoploides*	sp.	+	+	+
GU139759	Thoracostomopsidae	*Epacanthion*	sp.	+	+	–
HM564466	Thoracostomopsidae	*Enoplolaimus*	sp.	+	+	–
HM564427	Thoracostomopsidae	*Enoplolaimus*	sp.	+	+	–
HM564422	Thoracostomopsidae	*Enoplolaimus*	sp.	+	+	–
GU139757	Thoracostomopsidae	*Mesacanthion*	sp.	+	+	–
GU139755	Thoracostomopsidae	*Mesacanthion*	sp.	+	+	–
GU139761	Thoracostomopsidae	*Mesacanthoides*	sp.	+	+	–
KF591734	Thoracostomopsidae	*Mesacanthion*	*ungulatum*	+	+	–
GU139764	Thoracostomopsidae	*Oxyonchus*	sp.	+	+	–
GU139753	Thoracostomopsidae	*Trileptium*	sp.	+	+	–
GU139763	Thoracostomopsidae	*Trileptium*	sp.	+	+	–
FN433904	Phanodermatidae	*Phanoderma*	sp.	+	+	–
HM564510	Phanodermatidae	*Phanodermopsis*	sp.	+	+	–
HM564575	Phanodermatidae	*Phanodermopsis*	sp.	+	+	–
HM564523	Phanodermatidae	*Phanodermopsis*	sp.	+	+	–
AY692344	Anticomidae	*Anticoma*	sp.	+	–	–
HM564638	Anticomidae	*Anticoma*	sp.	+	+	–
HM564627	Anticomidae	*Anticoma*	sp.	+	+	–
HM564612	Anticomidae	*Cephalanticoma*	sp.	+	+	–
FN433899	Leptosomatidae	*Deontostoma*	sp.	+	+	+
FN433903	Leptosomatidae	*Thoracostoma*	*microlobatum*	+	+	+
FN433905	Leptosomatidae	*Thoracostoma*	*trachygaster*	+	+	+
FN433902	Leptosomatidae	*Pseudocella*	sp.	+	+	+
FN433901	Leptosomatidae	*Pseudocella*	sp.	+	+	+

Acc. number	Family or *superfamily	Genus	Species	complete dataset	"filtered" dataset	"long" dataset
HM564626	Leptosomatidae	*Leptosomatides*	sp.	+	+	–
HM564630	Leptosomatidae	*Synonchus*	sp.	+	+	–
HM564585	Trefusiidae	*Trefusia*	sp.	+	+	–
HM564478	Trefusiidae	*Trefusia*	sp.	+	+	–
HM564609	Trefusiidae	*Rhabdocoma*	sp.	+	+	–
HM564606	Trefusiidae	*Rhabdocoma*	sp.	+	+	–
AJ966509	Trefusiidae	*Trischistoma*	*monohystera*	+	+	+
FJ969142	Trefusiidae	*Trischistoma*	sp.	+	+	+
KJ636223	Trefusiidae	*Tripylina*	*arenicola*	+	+	+
JX867773	Trefusiidae	*Tripylina*	*zhejiangensis*	+	+	+
FJ040504	Tripyloididae	*Bathylaimus*	sp.	+	+	+
AY854201	Tripyloididae	*Bathylaimus*	sp.	+	+	+
AJ966476	Tripyloididae	*Bathylaimus*	*assimilis*	+	+	+
AY854202	Tripyloididae	*Tripyloides*	sp.	+	+	+
DQ394804	Tripyloididae	*Tripyloides*	sp.	+	+	–
HM564405	Tripyloididae	*Tripyloides*	sp.	+	+	–
AY590149	Anoplostomatidae	*Anoplostoma*	*rectospiculum*	+	+	+
FJ040491	Anoplostomatidae	*Anoplostoma*	sp.	+	+	+
FJ040492	Anoplostomatidae	*Anoplostoma*	sp.	+	+	+
HM564542	Anoplostomatidae	*Chaetonema*	sp.	+	+	–
HM564533	Anoplostomatidae	*Chaetonema*	sp.	+	+	–
FJ040501	Oxystominidae	*Halalaimus*	sp.	+	+	+
HM564540	Oxystominidae	*Halalaimus*	sp.	+	+	–
HM564652	Oxystominidae	*Halalaimus*	sp.	+	+	–
HM564521	Oxystominidae	*Halalaimus*	sp.	+	+	–
HM564420	Oxystominidae	*Halalaimus*	sp.	+	+	–
HM564481	Oxystominidae	*Oxystomina*	sp.	+	+	–
HM564548	Oxystominidae	*Oxystomina*	sp.	+	+	–
HM564403	Oxystominidae	*Oxystomina*	sp.	+	+	–
HM564651	Oxystominidae	*Oxystomina*	sp.	+	+	–
FJ040498	Oxystominidae	*Oxystomina*	sp.	+	+	+
HM564649	Oxystominidae	*Litinium*	sp.	+	+	–
HM564650	Oxystominidae	*Litinium*	sp.	+	+	–
HM564629	Oxystominidae	*Litinium*	sp.	+	+	–
FJ040500	Oxystominidae	*Thalassoalaimus*	*pirum*	+	+	+
HM564634	Oxystominidae	*Thalassoalaimus*	sp.	+	+	–
AY284738	Alaimidae	*Alaimus*	*parvus*	+	+	+
AJ966514	Alaimidae	*Alaimus*	sp.	+	+	+
FJ040489	Alaimidae	*Alaimus*	sp.	+	+	+

Acc. number	Family or *superfamily	Genus	Species	complete dataset	"filtered" dataset	"long" dataset
AY284739	Alaimidae	*Paramphidelus*	*hortensis*	+	+	+
AJ966487	Ironidae	*Ironus*	*dentifurcatus*	+	+	+
FJ040495	Ironidae	*Ironus*	*longicaudatus*	+	+	+
KJ636218	Ironidae	*Ironus*	*macramphis*	+	+	+
JQ071933	Ironidae	*Trissonchulus*	sp.	+	+	+
JQ071931	Ironidae	*Trissonchulus*	sp.	+	+	+
HM564604	Ironidae	*Dolicholaimus*	sp.	+	+	–
AY854200	Ironidae	*Syringolaimus*	*striatocaudatus*	+	+	+
FJ040497	Ironidae	*Syringolaimus*	sp.	+	+	+
FJ969139	Rhabdolaimidae	*Rhabdolaimus*	*aquaticus*	+	+	+
KJ636366	Rhabdolaimidae	*Rhabdolaimus*	*terrestris*	+	+	+
AF342790	Priapulidae	*Halicryptus*	*spinulosus*	+	+	+
X80234	Priapulidae	*Priapulus*	*caudatus*	+	+	+
X87984	Priapulidae	*Priapulus*	*caudatus*	+	+	+
Z38009	Priapulidae	*Priapulus*	*caudatus*	+	+	+
