# Supplementary figures and images for: Metabarcoding of marine nematodes – evaluation of reference datasets used in tree-based taxonomy assignment approach
Source: Biodivers Data J. 2016 Sep 21;(4):e10021. doi: 10.3897/BDJ.4.e10021 (PMC5136706; doi:10.3897/BDJ.4.e10021)

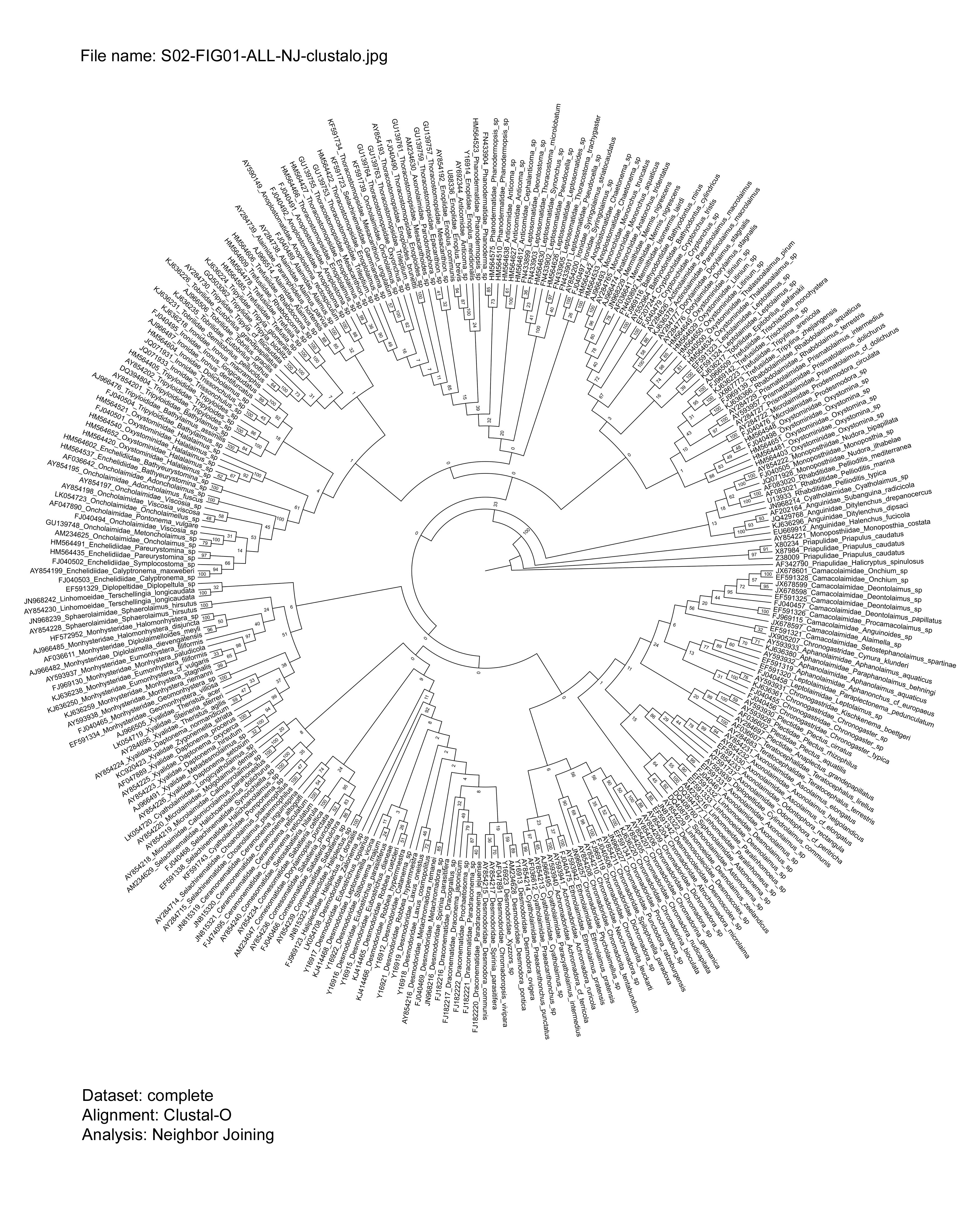

Supplement: Supplementary material 2 — Neighbor joining tree inferred using Clustal-O alignment of the complete dataset [file biodiversity_data_journal-4-e10021-s002.jpg]

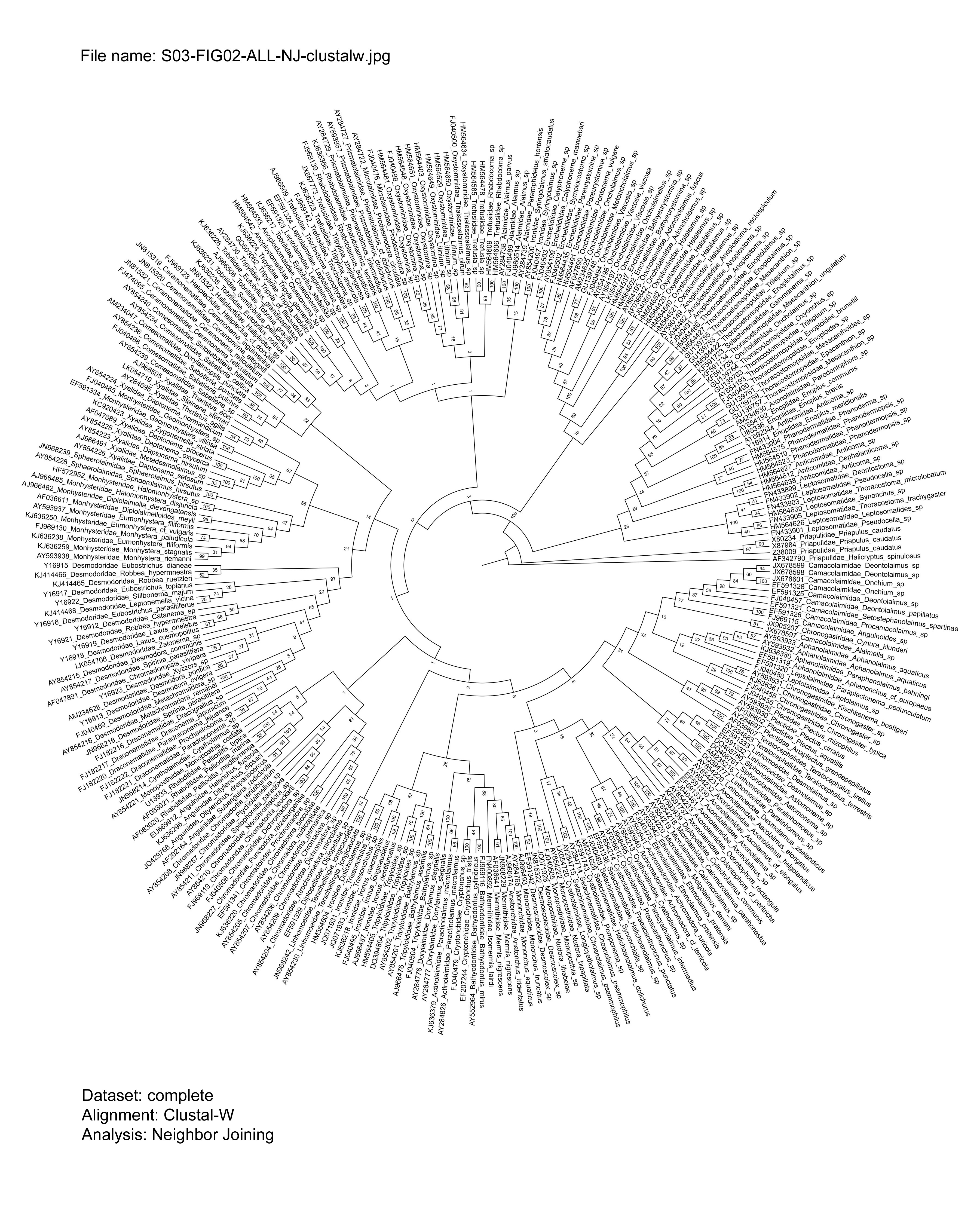

Supplement: Supplementary material 3 — Neighbor joining tree inferred using Clustal-W alignment of the complete dataset [file biodiversity_data_journal-4-e10021-s003.jpg]

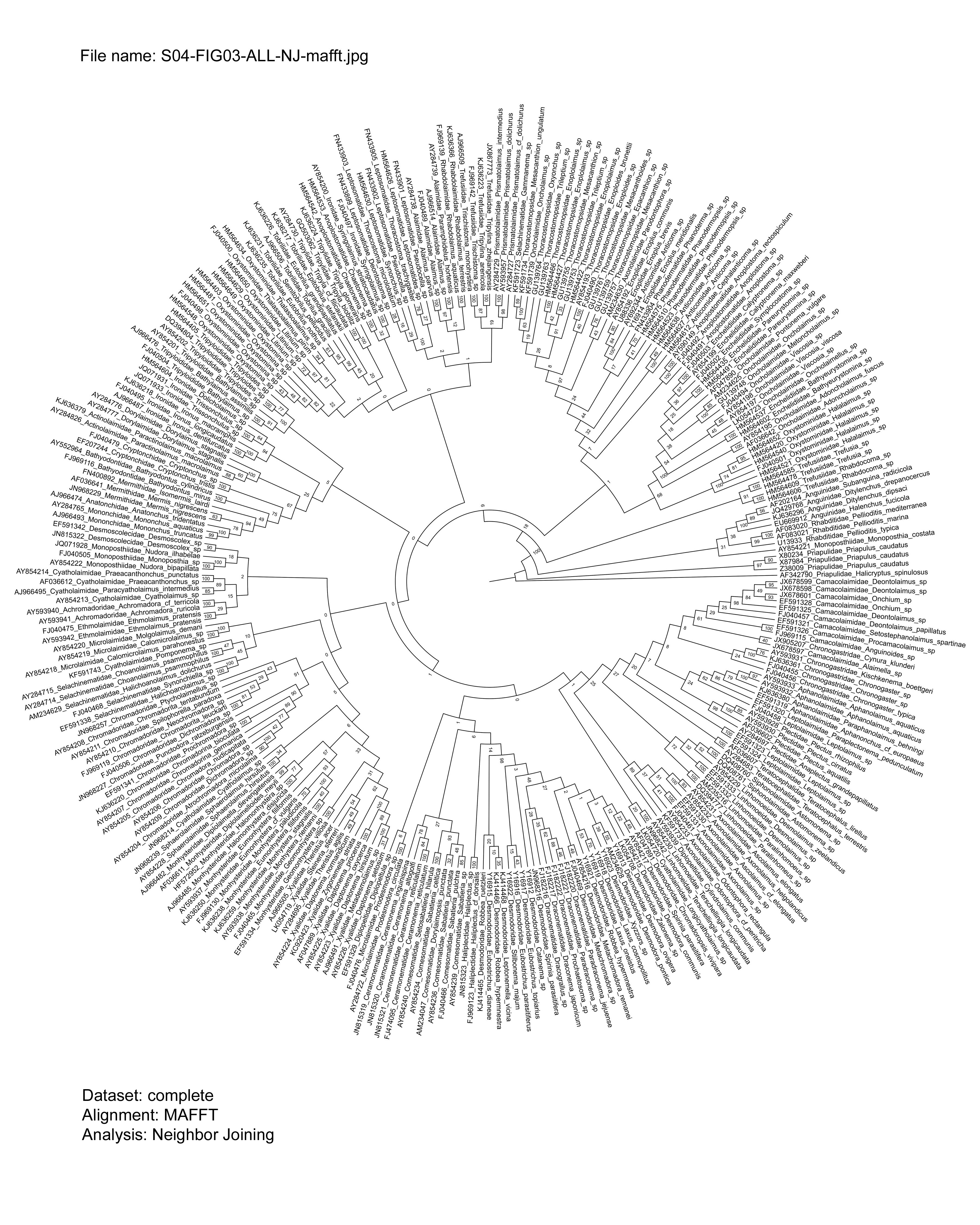

Supplement: Supplementary material 4 — Neighbor joining tree inferred using MAFFT alignment of the complete dataset [file biodiversity_data_journal-4-e10021-s004.jpg]

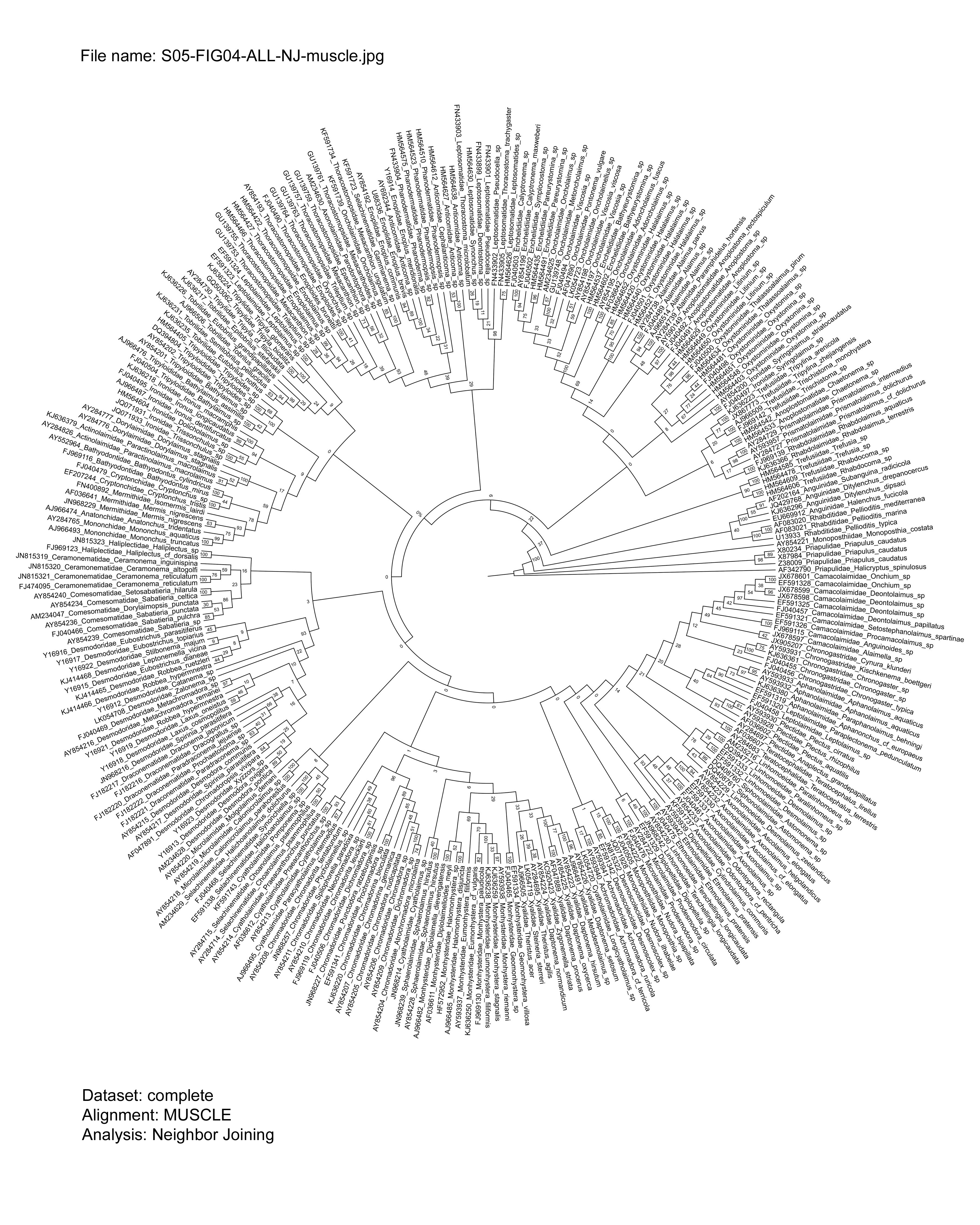

Supplement: Supplementary material 5 — Neighbor joining tree inferred using MUSCLE alignment of the complete dataset [file biodiversity_data_journal-4-e10021-s005.jpg]

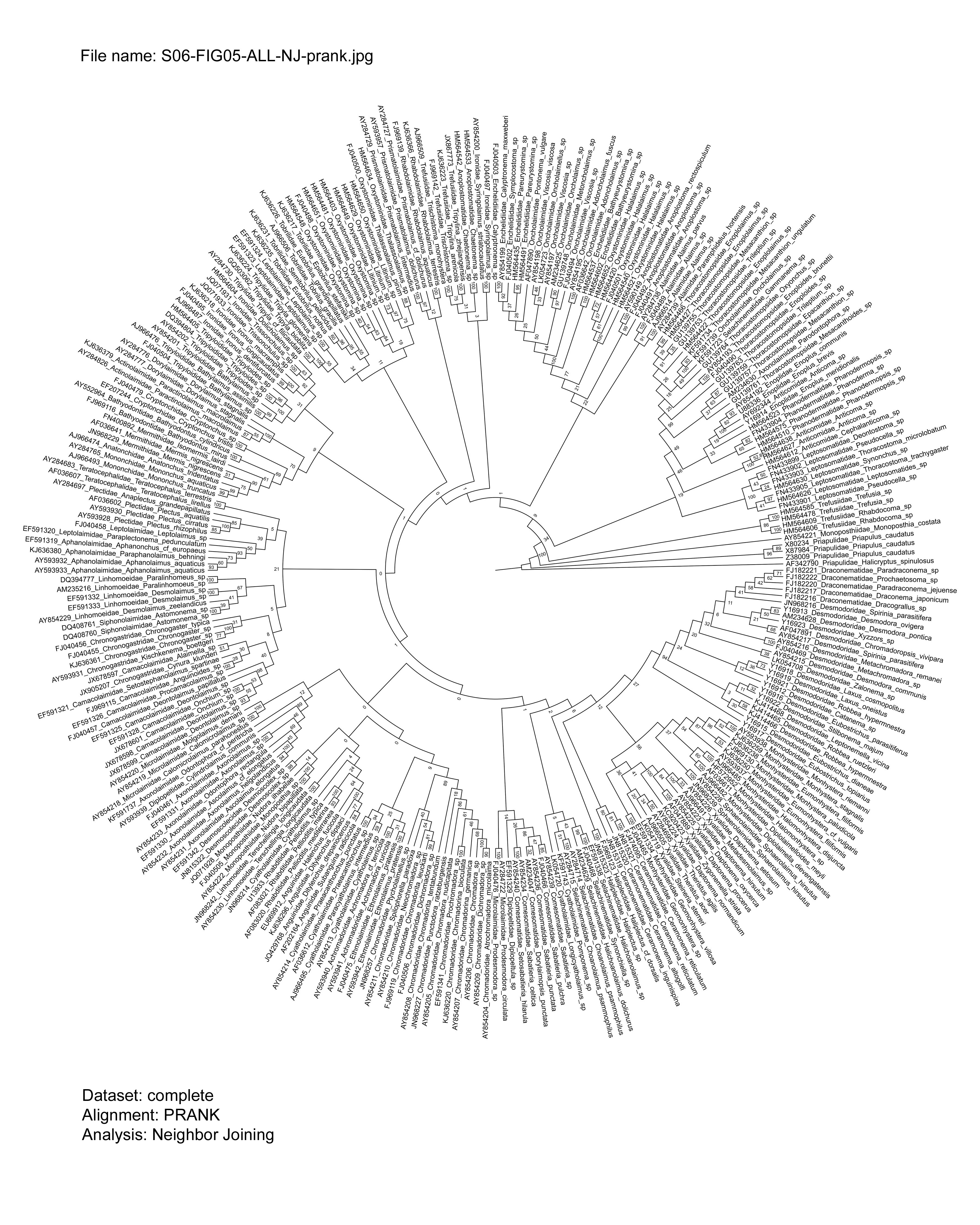

Supplement: Supplementary material 6 — Neighbor joining tree inferred using PRANK alignment of the complete dataset [file biodiversity_data_journal-4-e10021-s006.jpg]

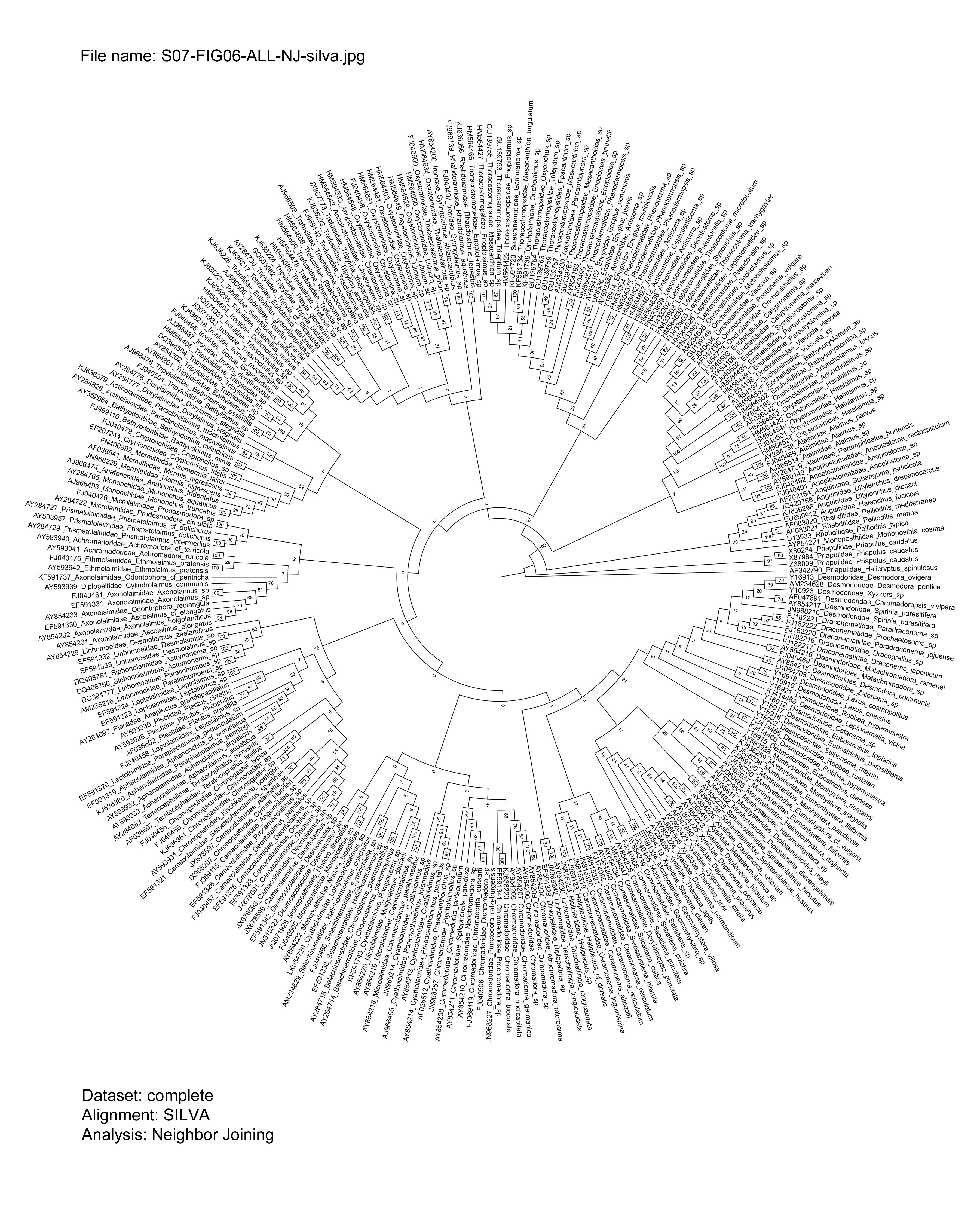

Supplement: Supplementary material 7 — Neighbor joining tree inferred using SILVA-based alignment of the complete dataset [file biodiversity_data_journal-4-e10021-s007.jpg]

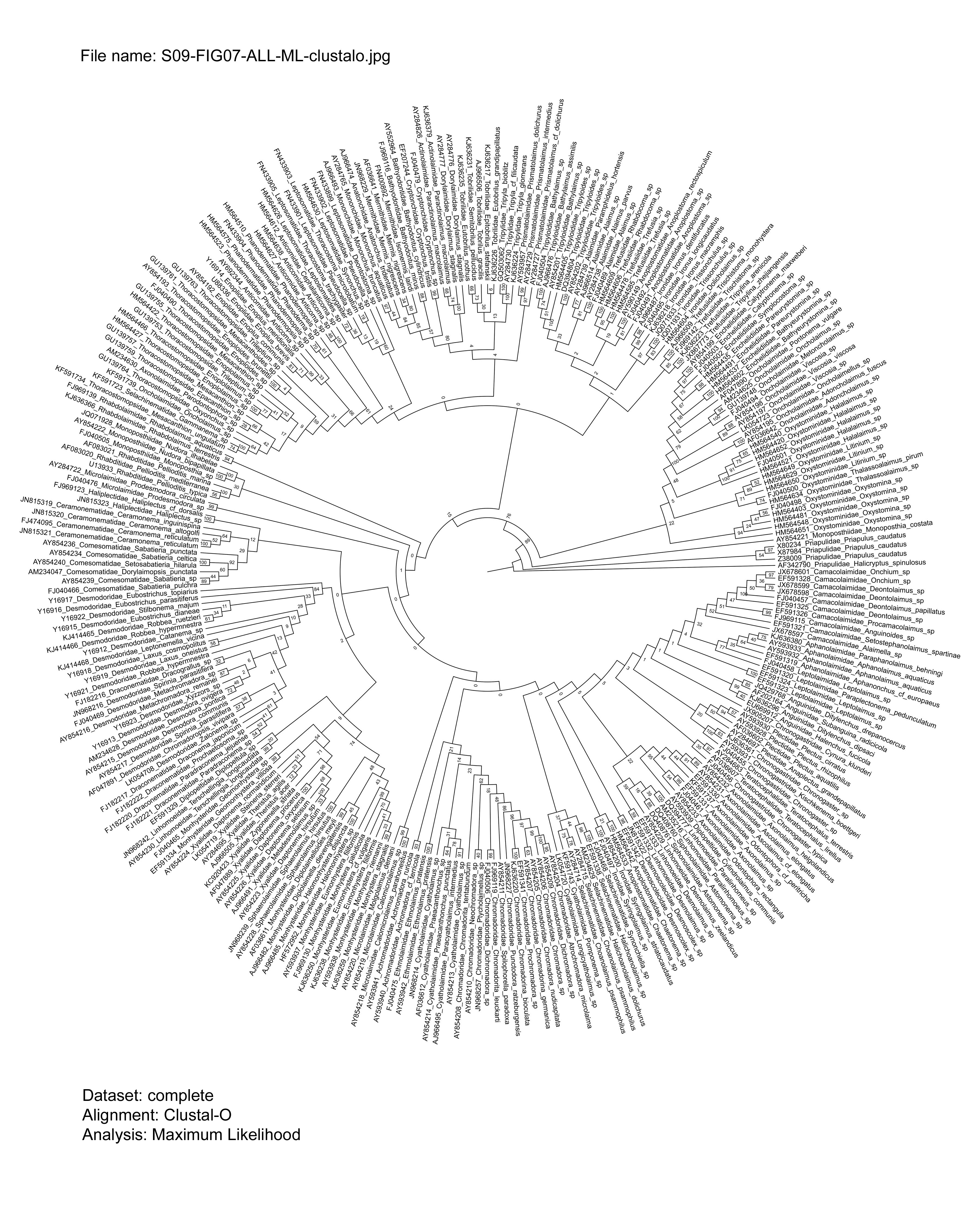

Supplement: Supplementary material 9 — Maximum likelihood tree inferred using Clustal-O alignment of the complete dataset [file biodiversity_data_journal-4-e10021-s009.jpg]

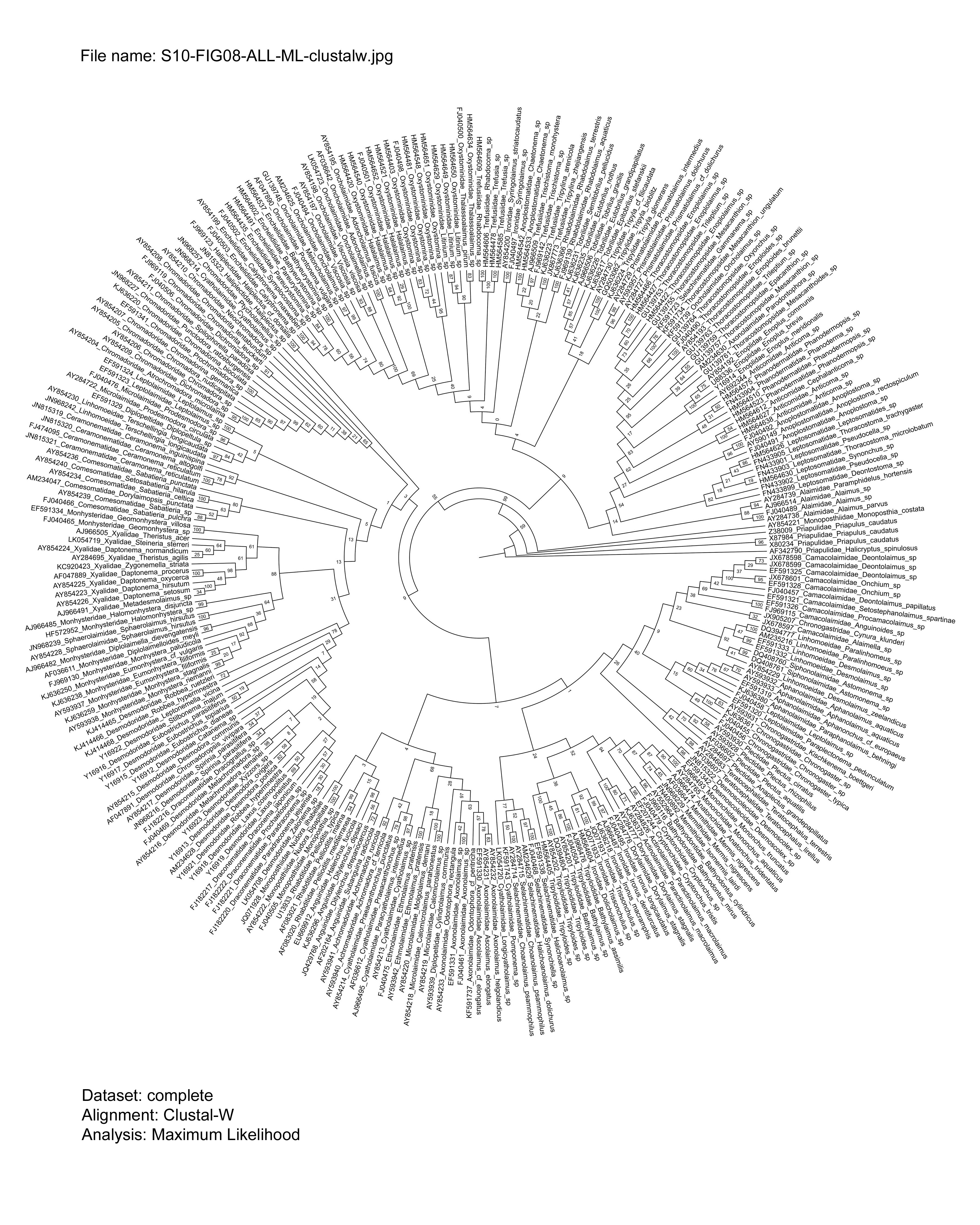

Supplement: Supplementary material 10 — Maximum likelihood tree inferred using Clustal-W alignment of the complete dataset [file biodiversity_data_journal-4-e10021-s010.jpg]

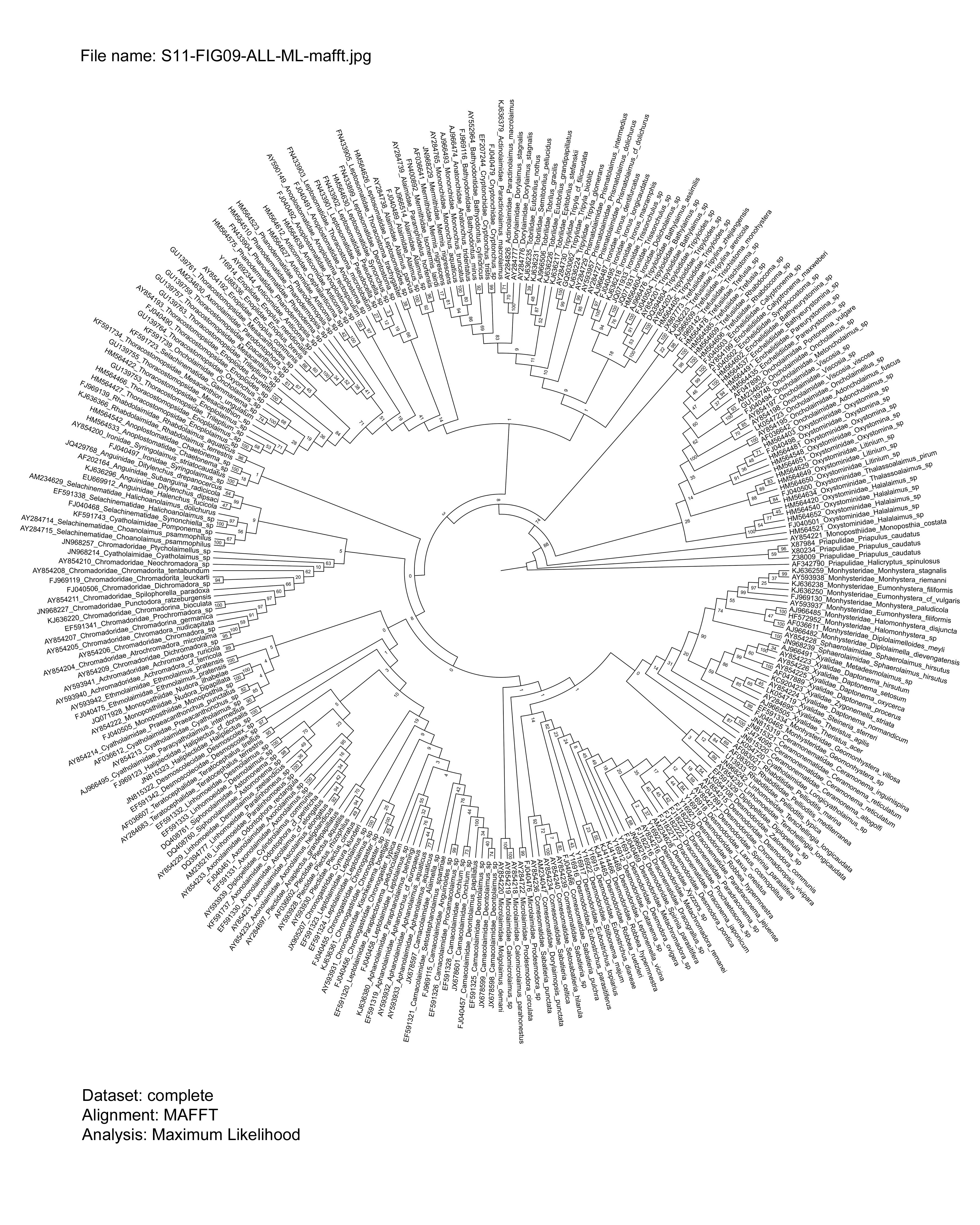

Supplement: Supplementary material 11 — Maximum likelihood tree inferred using MAFFT alignment of the complete dataset [file biodiversity_data_journal-4-e10021-s011.jpg]

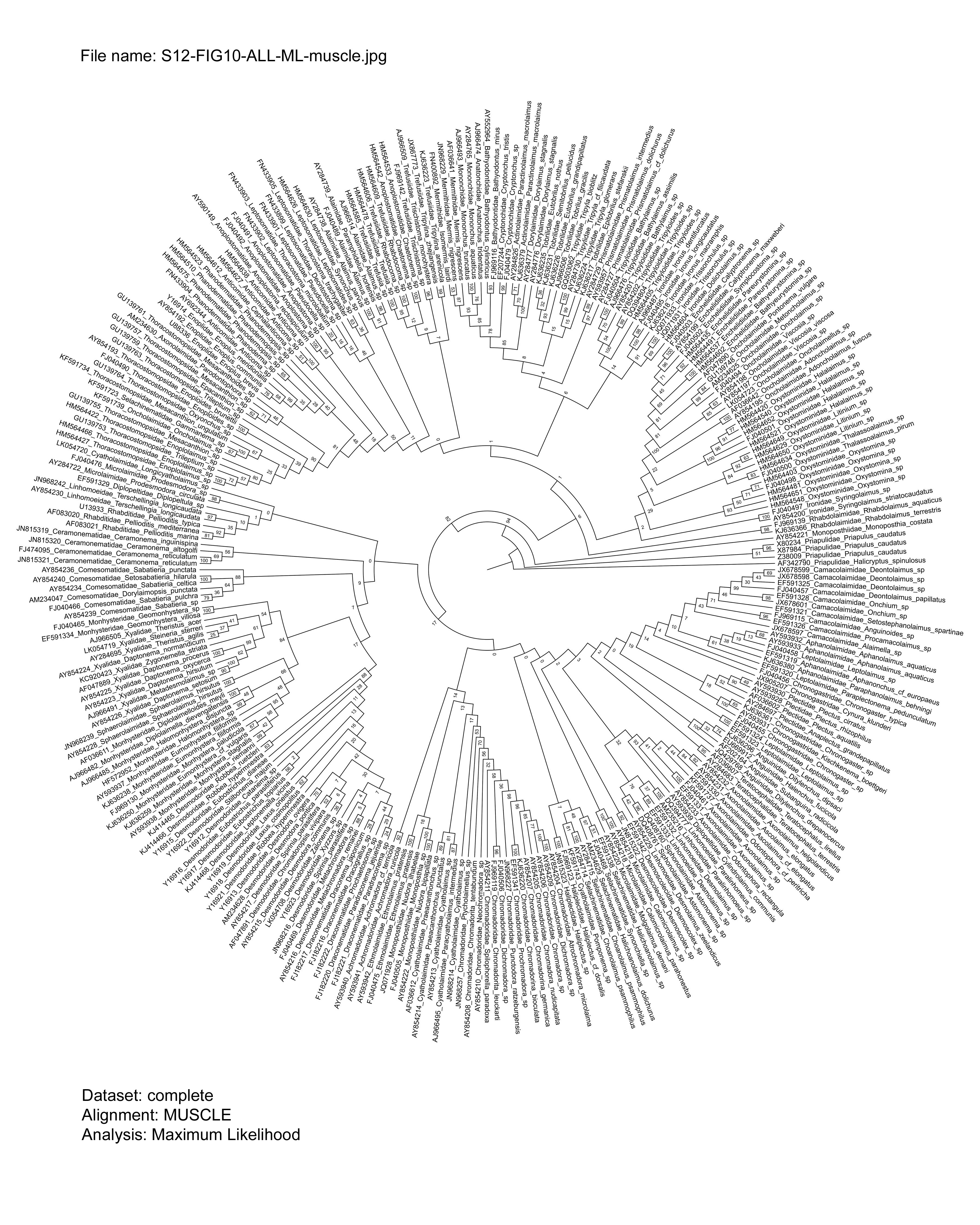

Supplement: Supplementary material 12 — Maximum likelihood tree inferred using MUSCLE alignment of the complete dataset [file biodiversity_data_journal-4-e10021-s012.jpg]

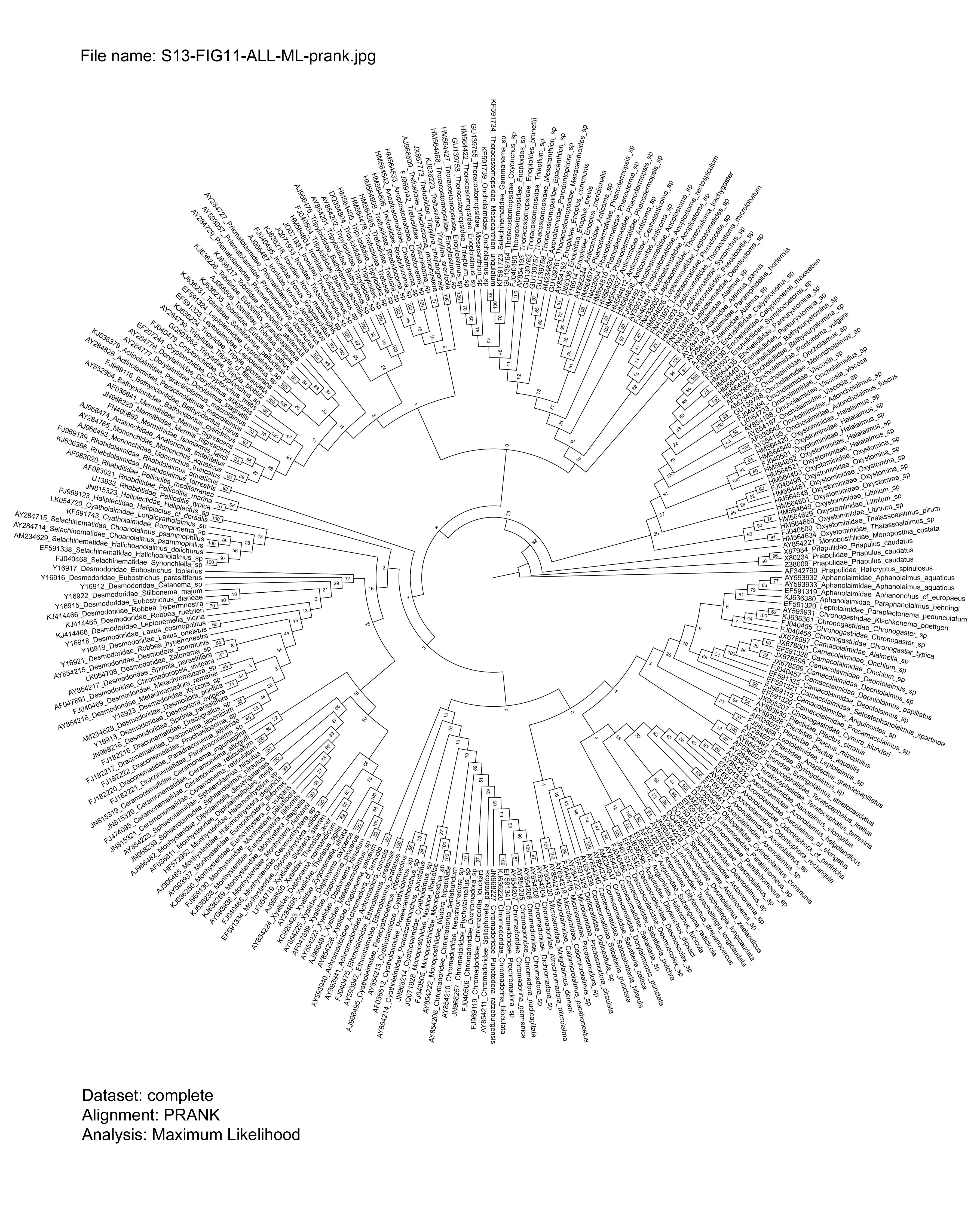

Supplement: Supplementary material 13 — Maximum likelihood tree inferred using PRANK alignment of the complete dataset [file biodiversity_data_journal-4-e10021-s013.jpg]

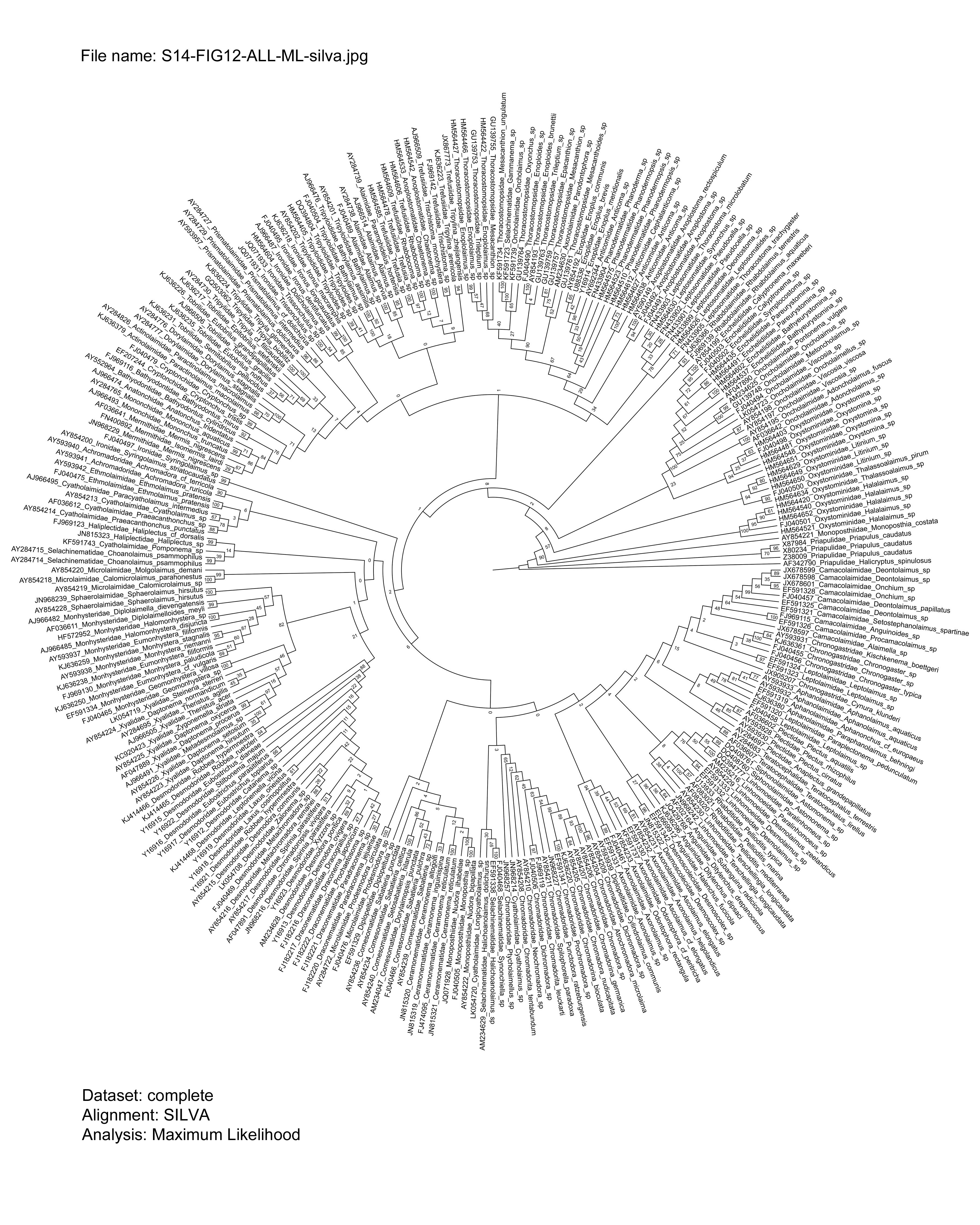

Supplement: Supplementary material 14 — Maximum likelihood tree inferred using SILVA-based alignment of the complete dataset [file biodiversity_data_journal-4-e10021-s014.jpg]

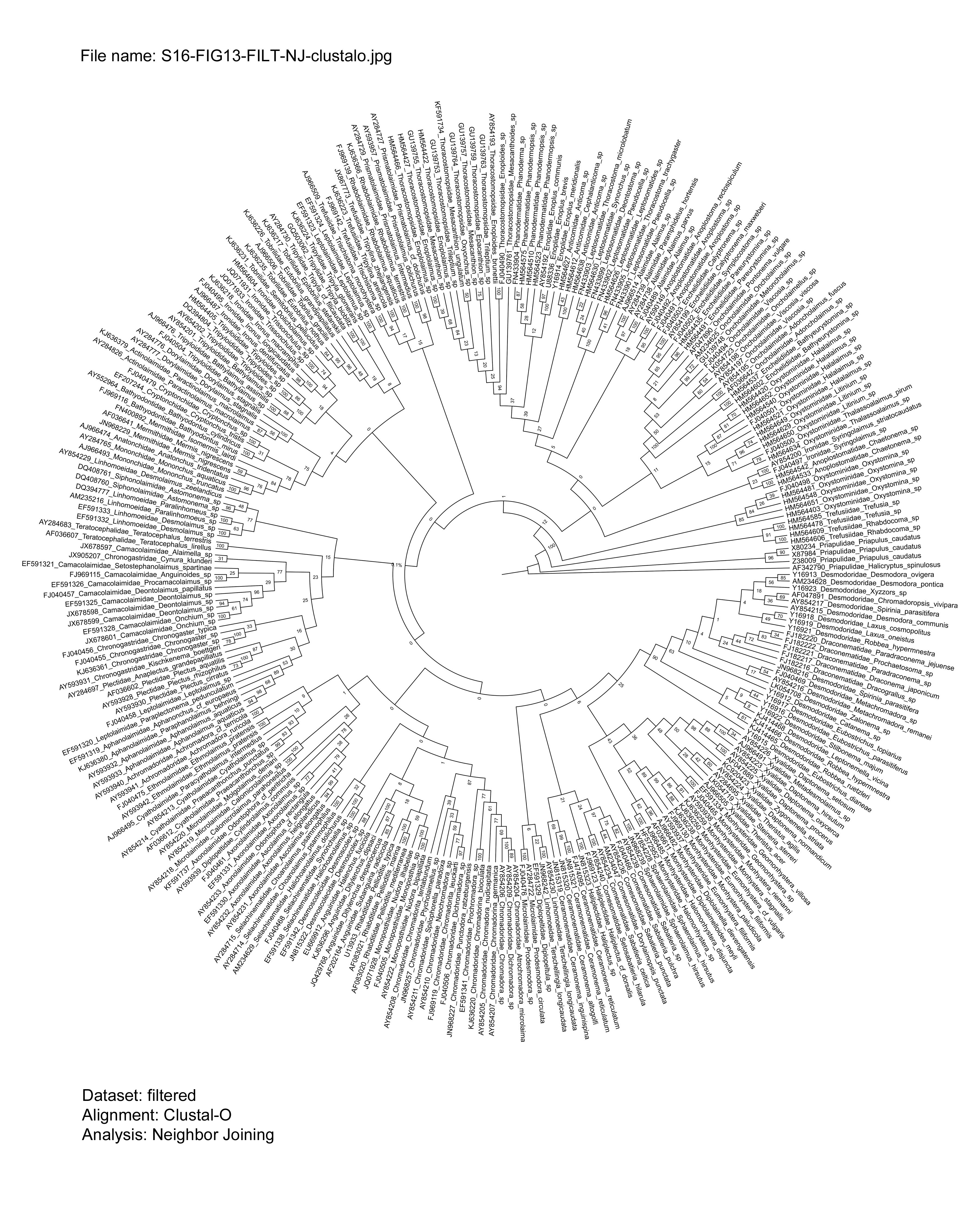

Supplement: Supplementary material 16 — Neighbor joining tree inferred using Clustal-O alignment of the "filtered" dataset [file biodiversity_data_journal-4-e10021-s016.jpg]

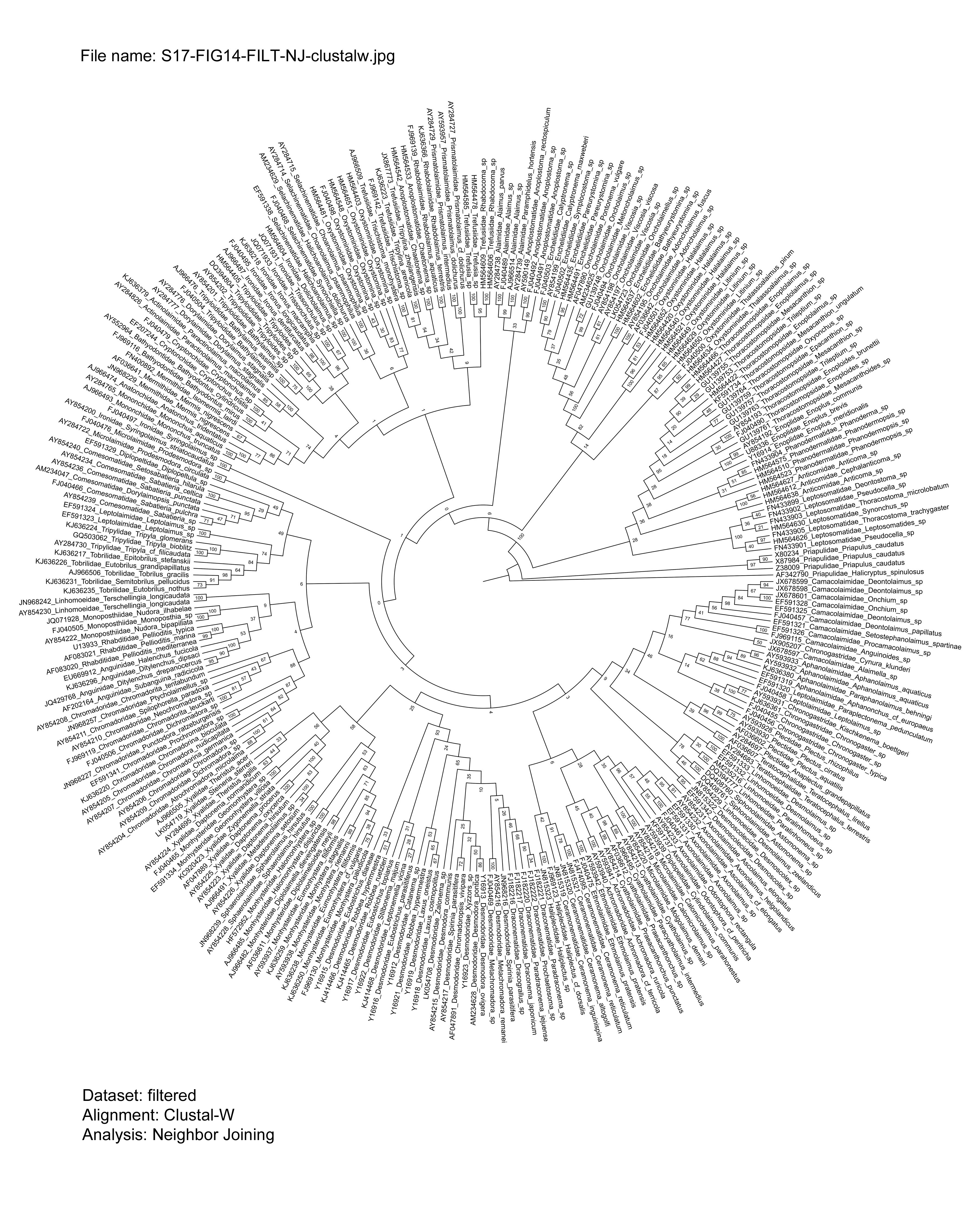

Supplement: Supplementary material 17 — Neighbor joining tree inferred using Clustal-W alignment of the "filtered" dataset [file biodiversity_data_journal-4-e10021-s017.jpg]

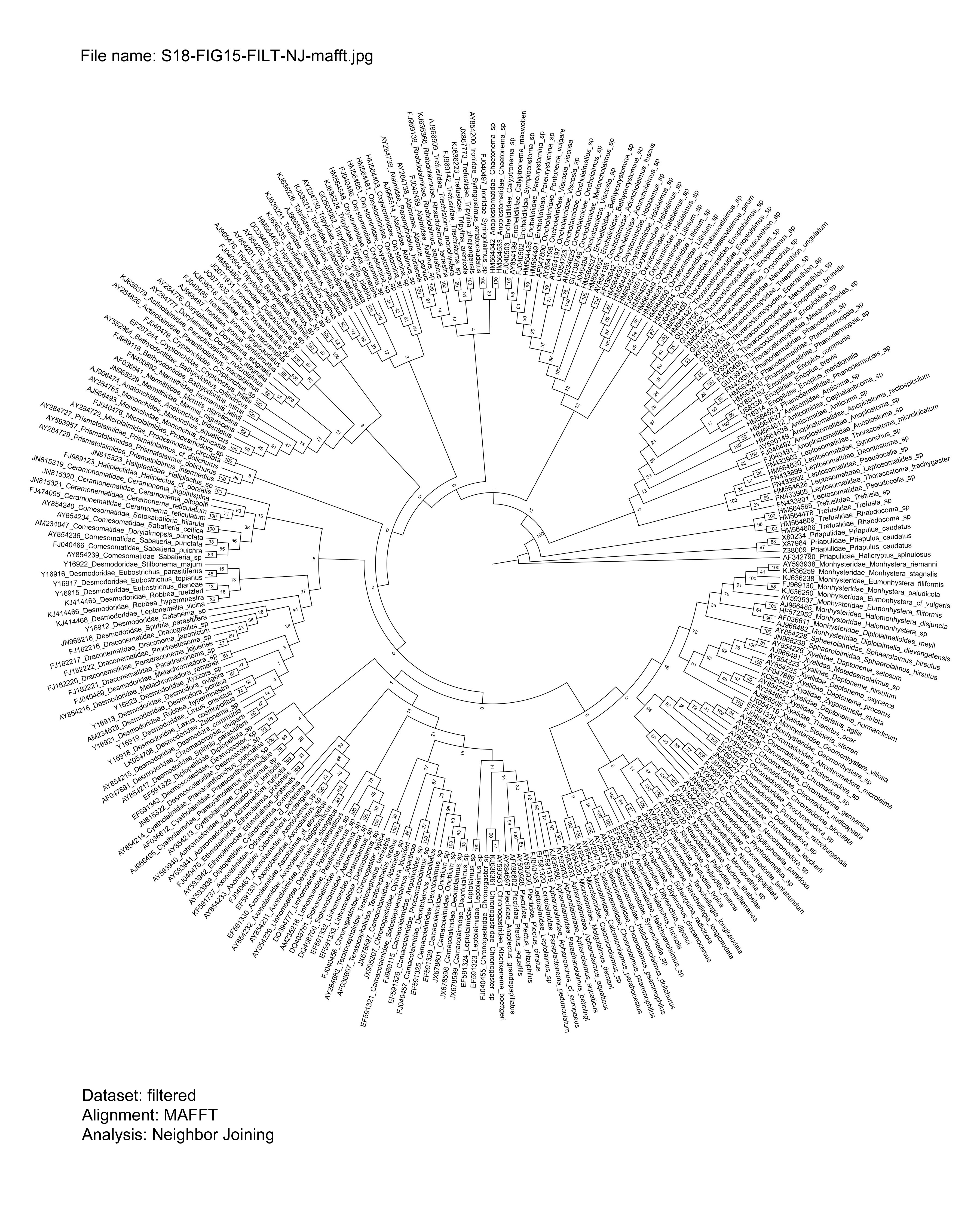

Supplement: Supplementary material 18 — Neighbor joining tree inferred using MAFFT alignment of the "filtered" dataset [file biodiversity_data_journal-4-e10021-s018.jpg]

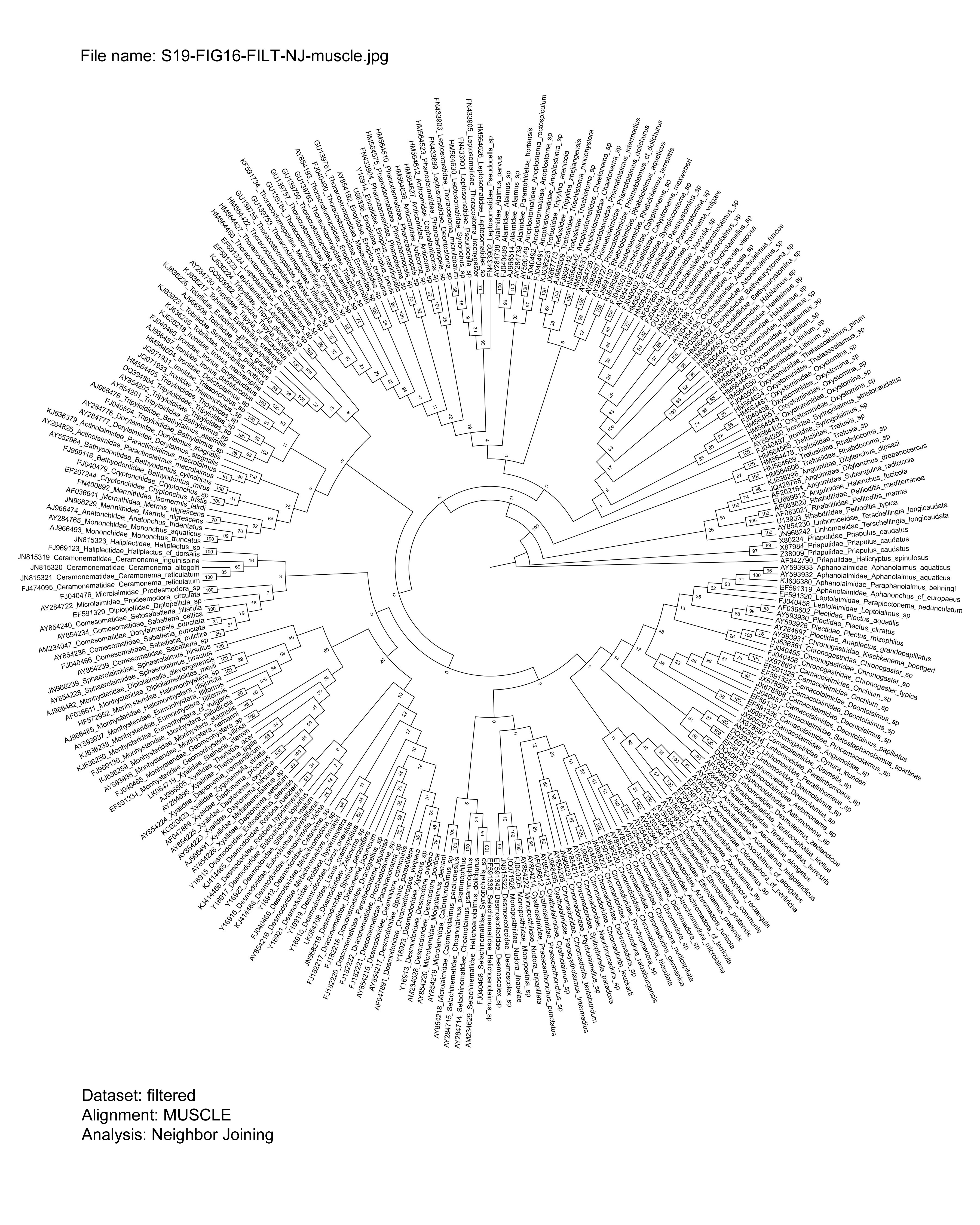

Supplement: Supplementary material 19 — Neighbor joining tree inferred using MUSCLE alignment of the "filtered" dataset [file biodiversity_data_journal-4-e10021-s019.jpg]

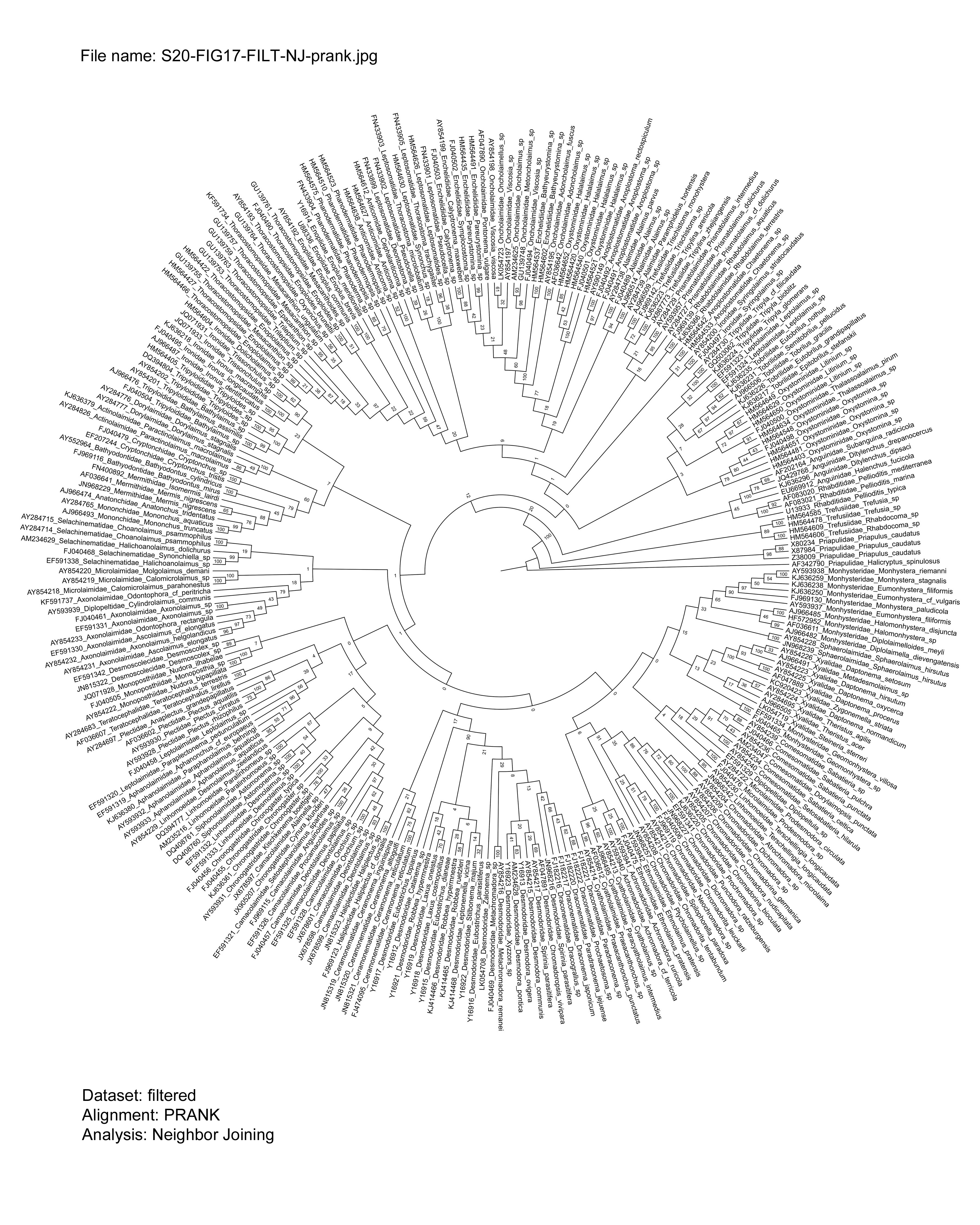

Supplement: Supplementary material 20 — Neighbor joining tree inferred using PRANK alignment of the "filtered" dataset [file biodiversity_data_journal-4-e10021-s020.jpg]

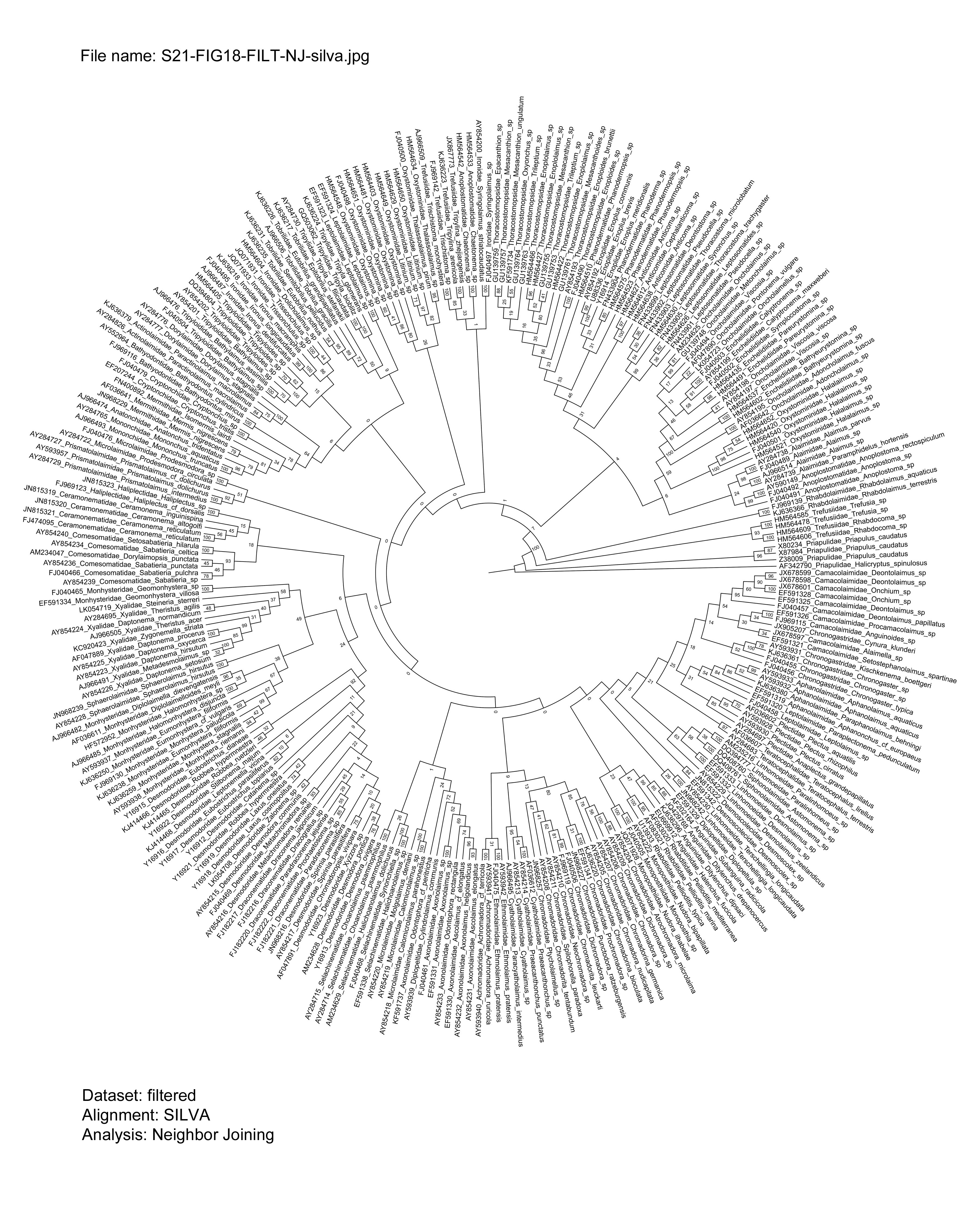

Supplement: Supplementary material 21 — Neighbor joining tree inferred using SILVA-based alignment of the "filtered" dataset [file biodiversity_data_journal-4-e10021-s021.jpg]

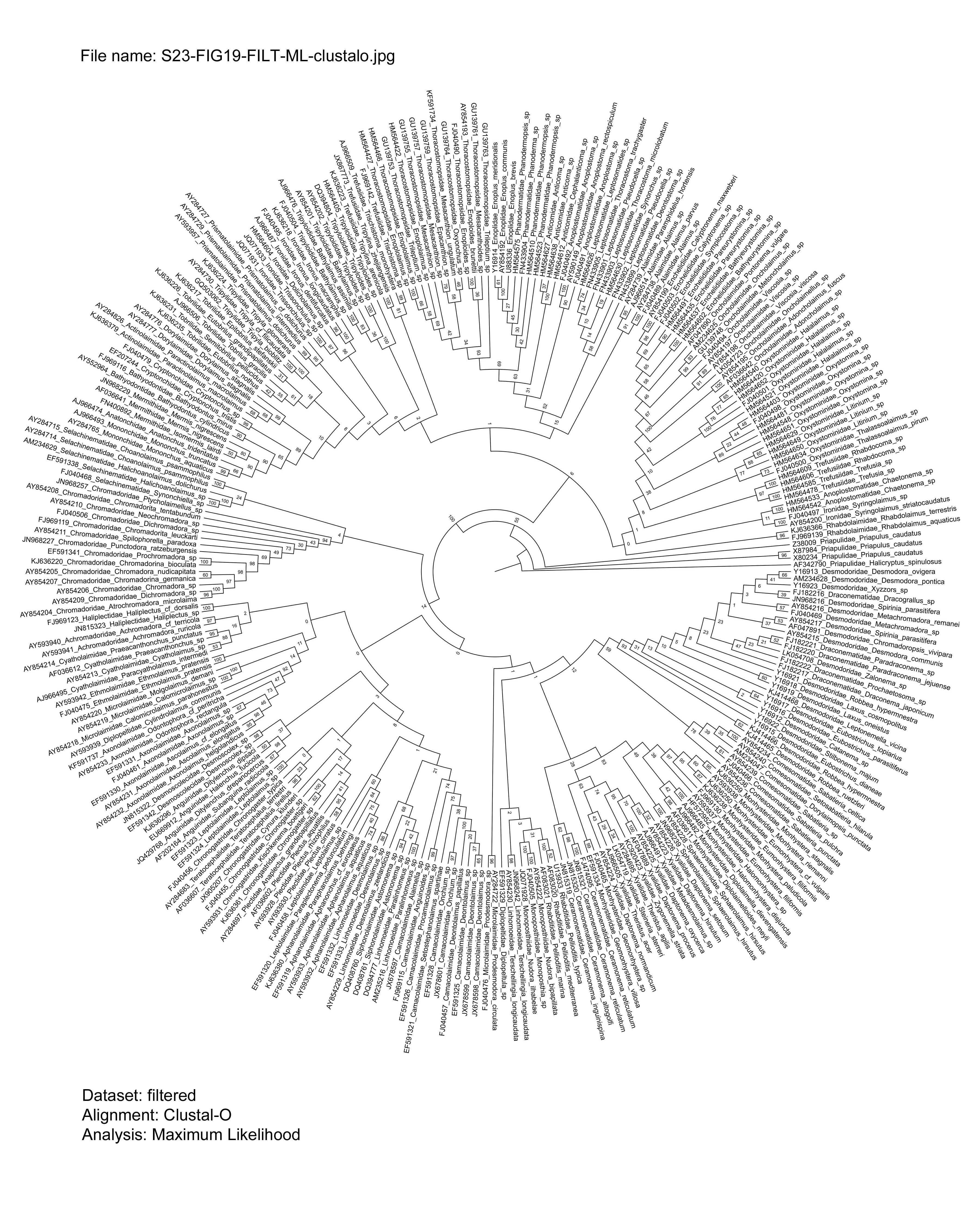

Supplement: Supplementary material 23 — Maximum likelihood tree inferred using Clustal-O alignment of the "filtered" dataset [file biodiversity_data_journal-4-e10021-s023.jpg]

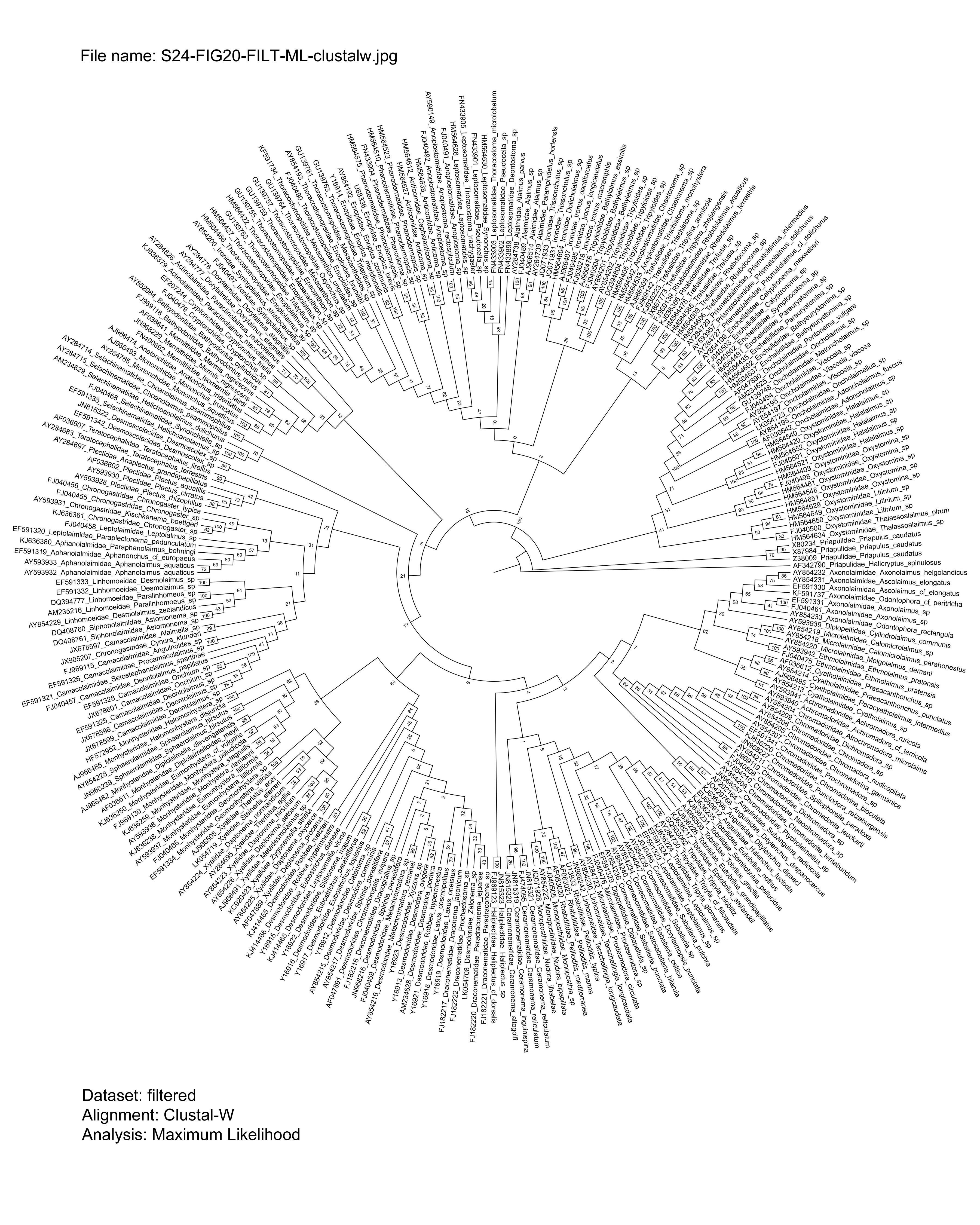

Supplement: Supplementary material 24 — Maximum likelihood tree inferred using Clustal-W alignment of the "filtered" dataset [file biodiversity_data_journal-4-e10021-s024.jpg]

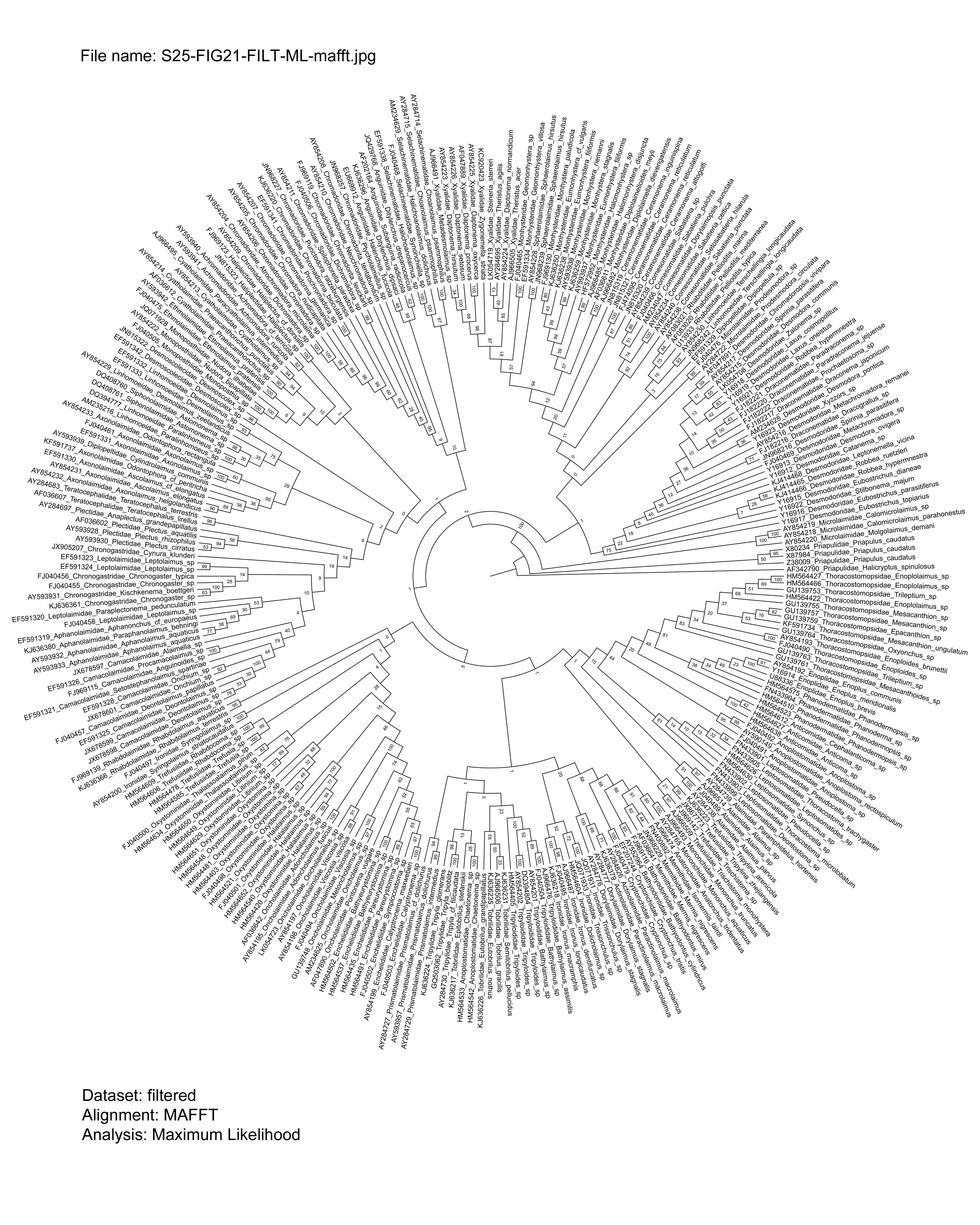

Supplement: Supplementary material 25 — Maximum likelihood tree inferred using MAFFT alignment of the "filtered" dataset [file biodiversity_data_journal-4-e10021-s025.jpg]

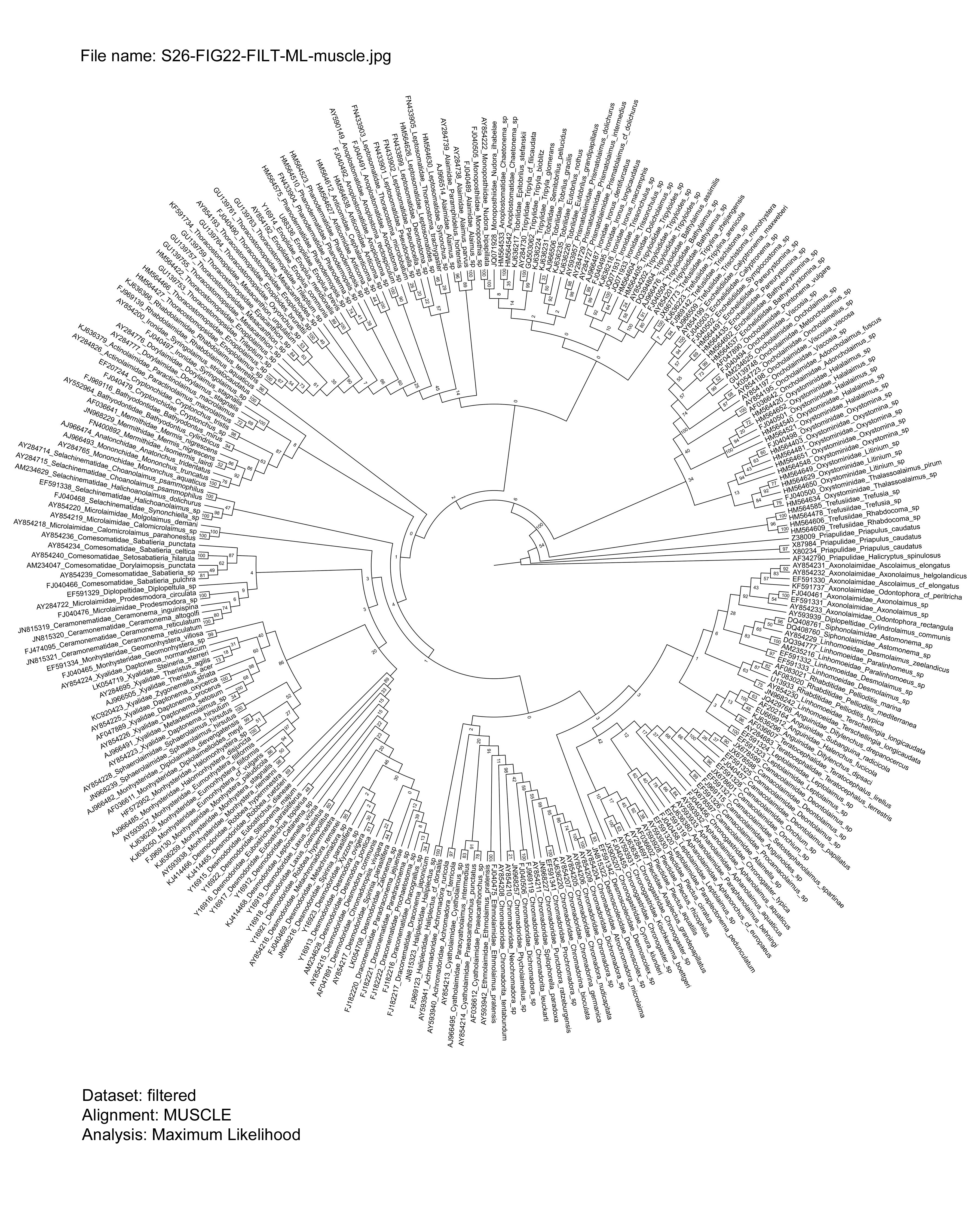

Supplement: Supplementary material 26 — Maximum likelihood tree inferred using MUSCLE alignment of the "filtered" dataset [file biodiversity_data_journal-4-e10021-s026.jpg]

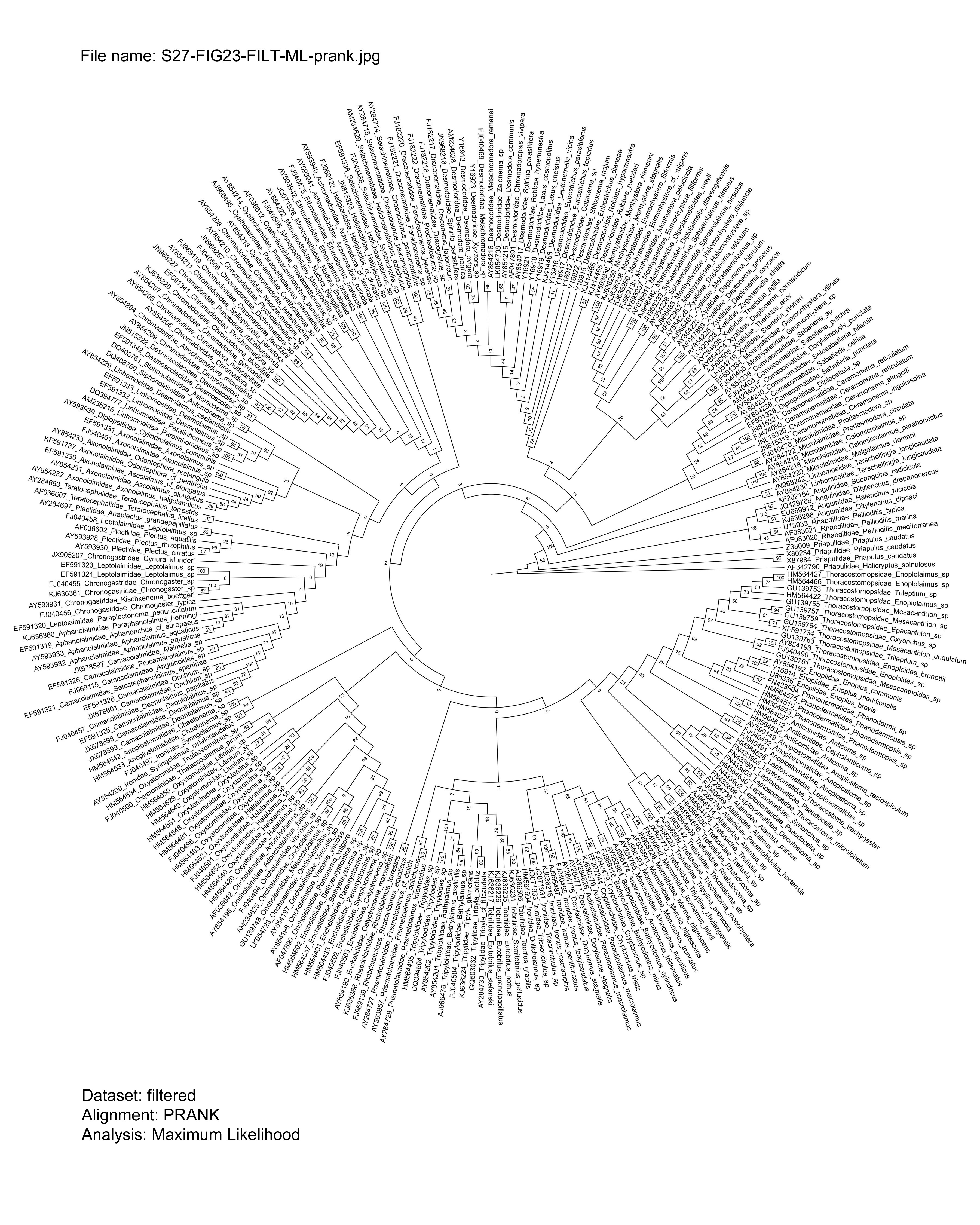

Supplement: Supplementary material 27 — Maximum likelihood tree inferred using PRANK alignment of the "filtered" dataset [file biodiversity_data_journal-4-e10021-s027.jpg]

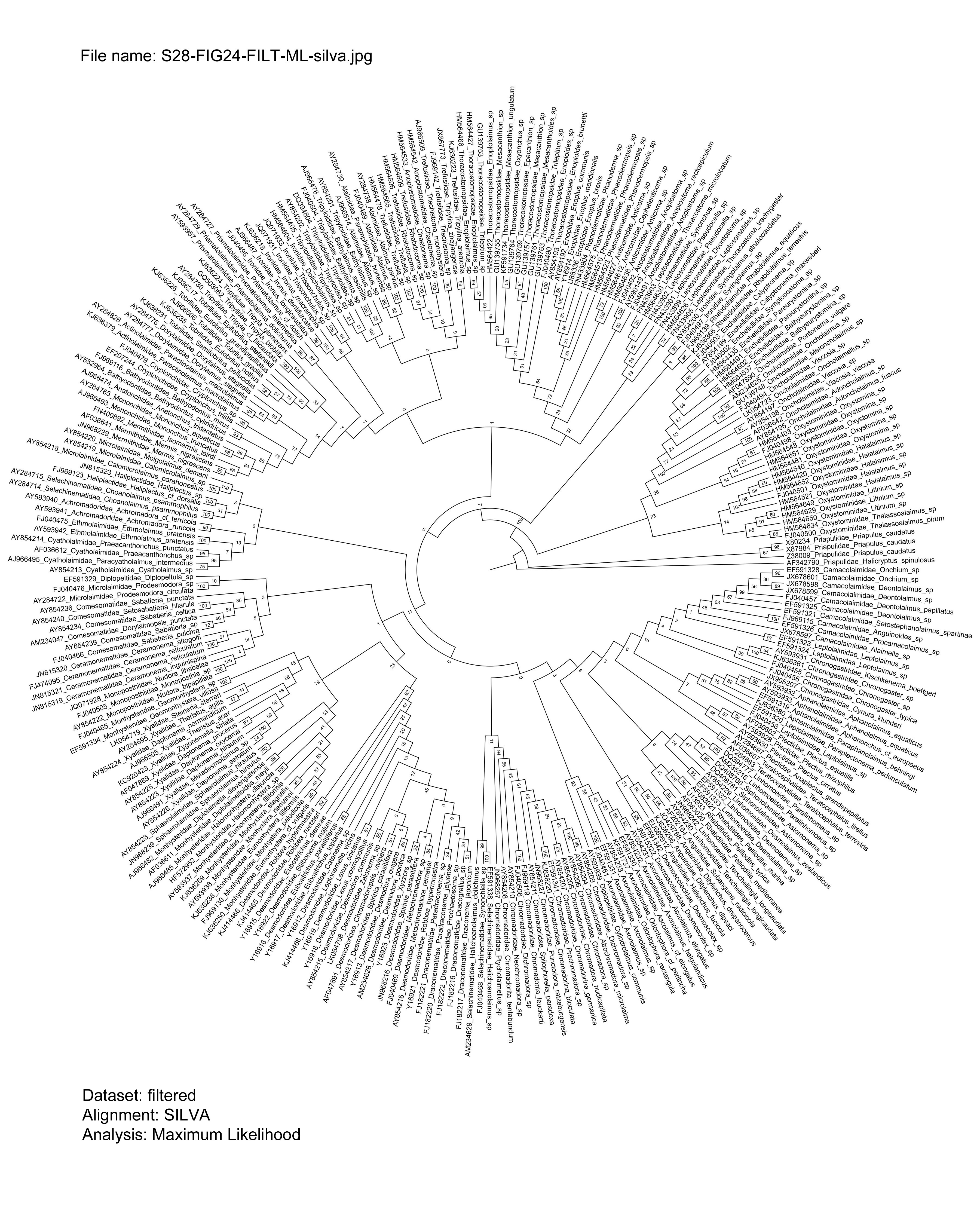

Supplement: Supplementary material 28 — Maximum likelihood tree inferred using SILVA-based alignment of the "filtered" dataset [file biodiversity_data_journal-4-e10021-s028.jpg]

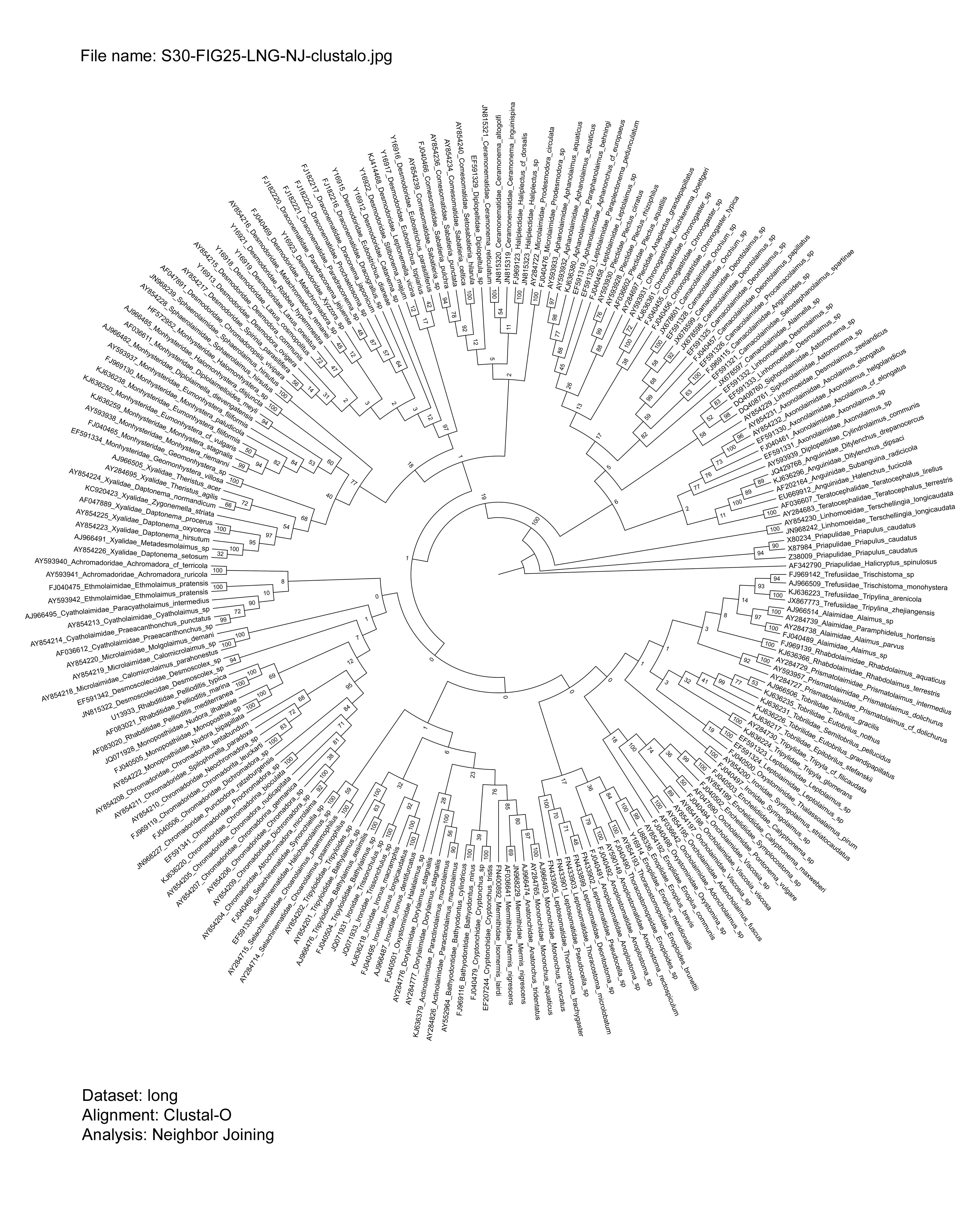

Supplement: Supplementary material 30 — Neighbor joining tree inferred using Clustal-O alignment of the "long" dataset [file biodiversity_data_journal-4-e10021-s030.jpg]

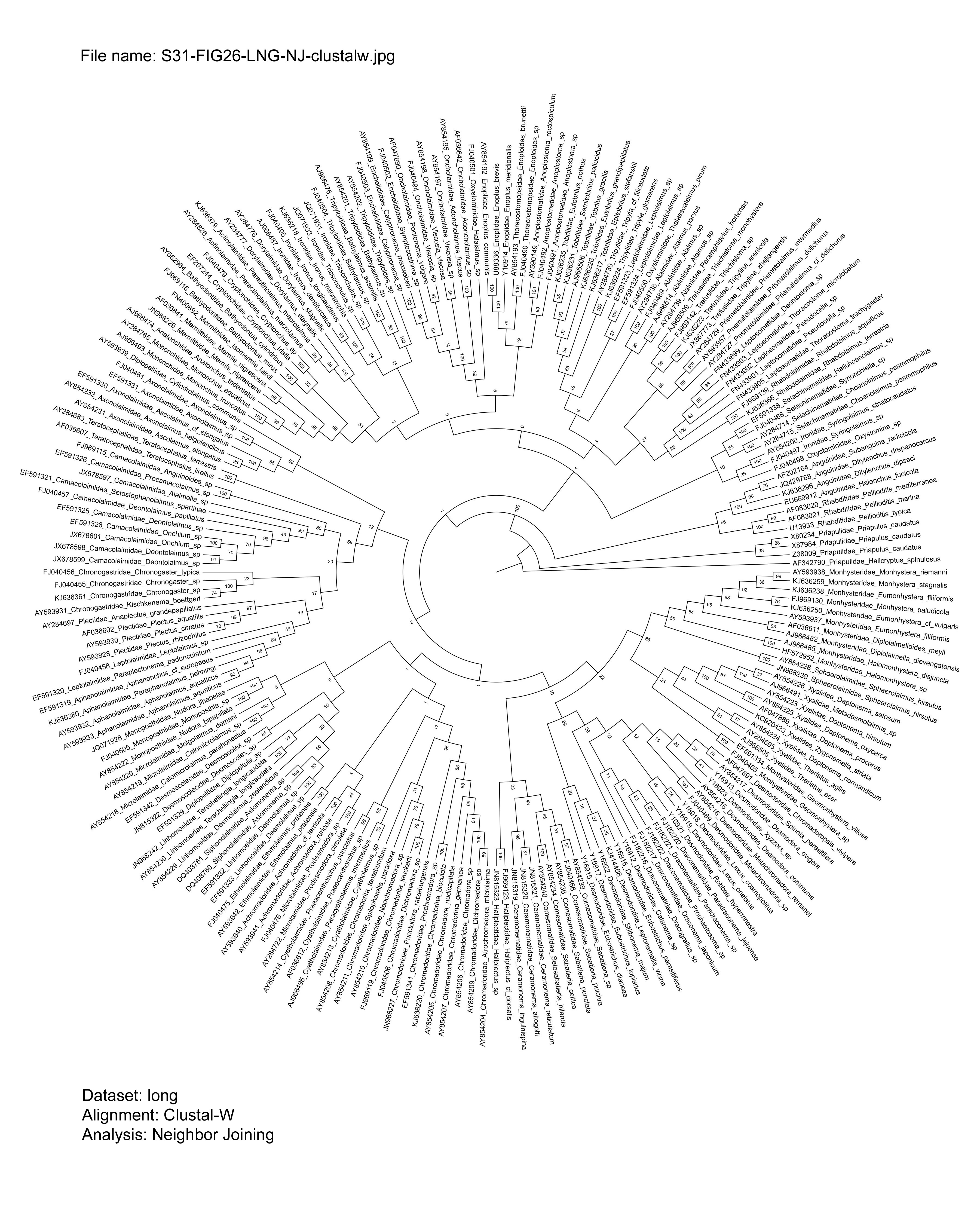

Supplement: Supplementary material 31 — Neighbor joining tree inferred using Clustal-W alignment of the "long" dataset [file biodiversity_data_journal-4-e10021-s031.jpg]

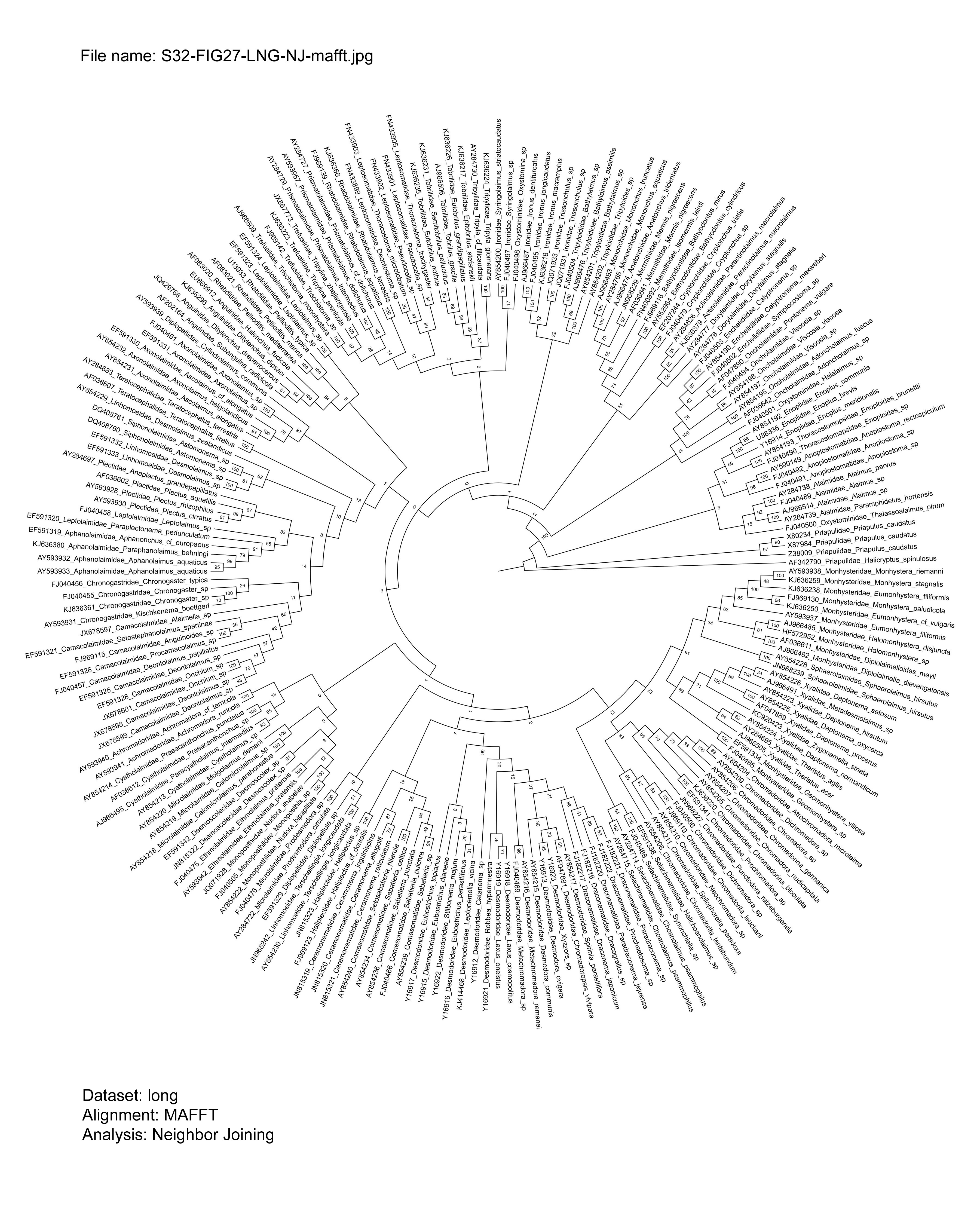

Supplement: Supplementary material 32 — Neighbor joining tree inferred using MAFFT alignment of the "long" dataset [file biodiversity_data_journal-4-e10021-s032.jpg]

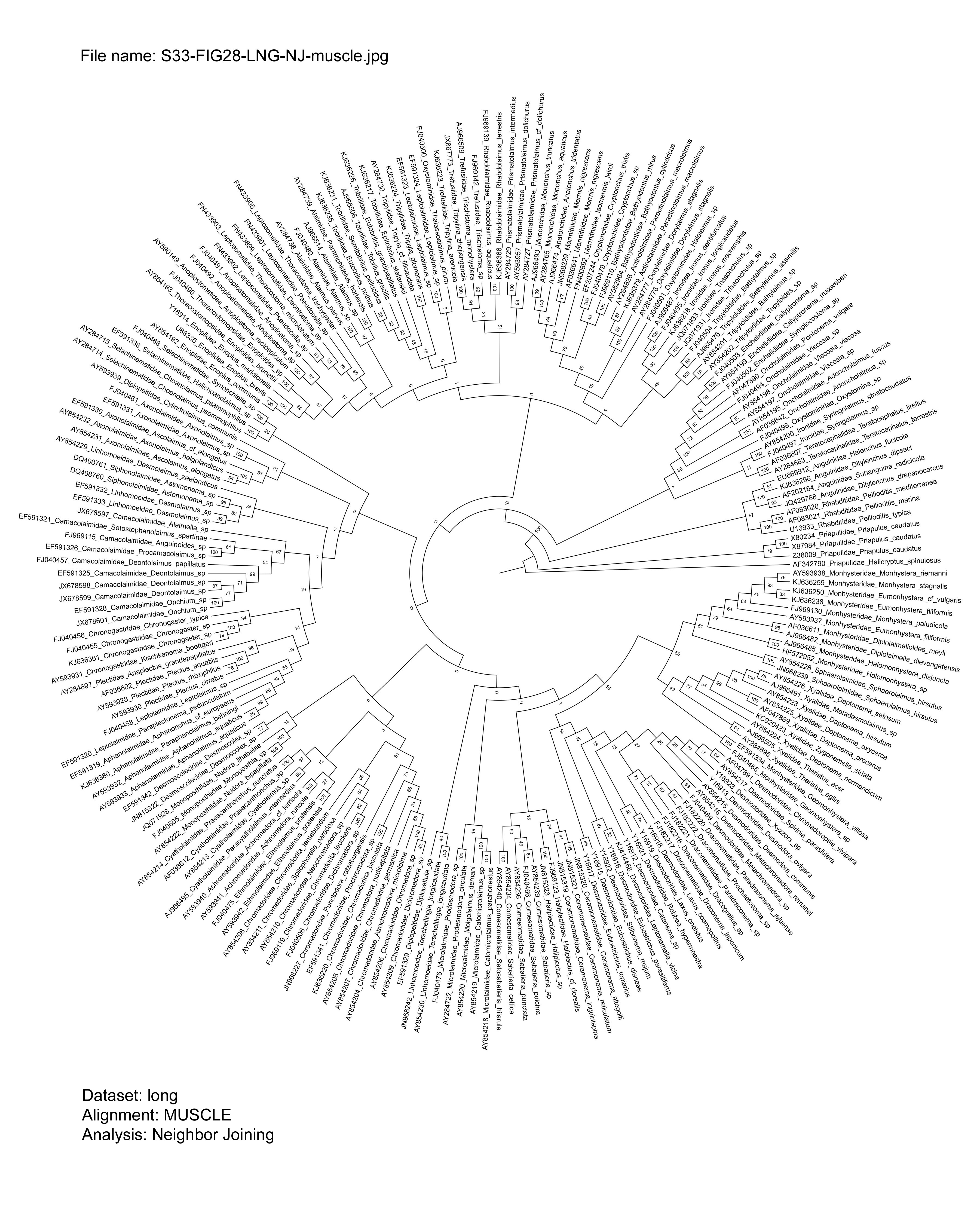

Supplement: Supplementary material 33 — Neighbor joining tree inferred using MUSCLE alignment of the "long" dataset [file biodiversity_data_journal-4-e10021-s033.jpg]

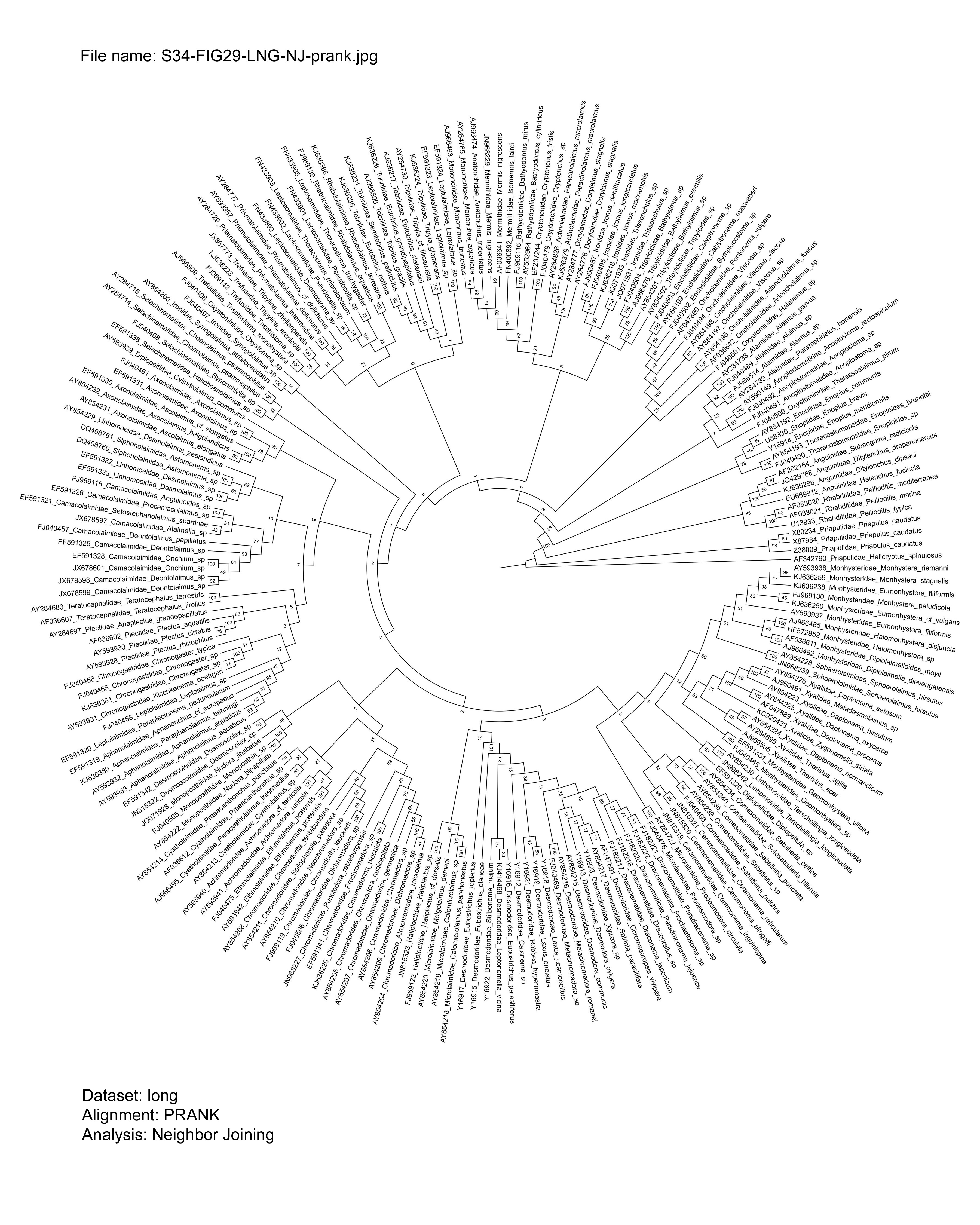

Supplement: Supplementary material 34 — Neighbor joining tree inferred using PRANK alignment of the "long" dataset [file biodiversity_data_journal-4-e10021-s034.jpg]

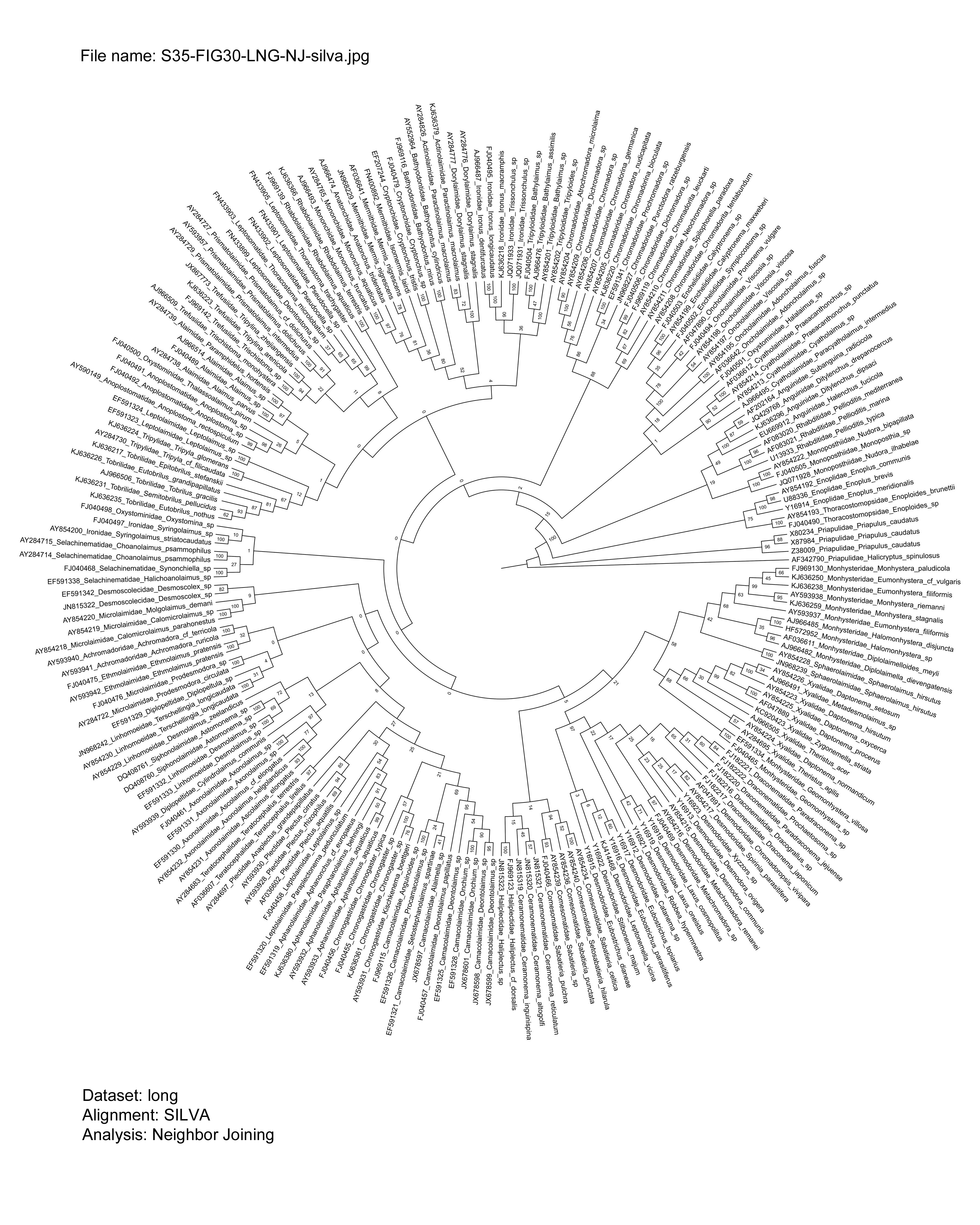

Supplement: Supplementary material 35 — Neighbor joining tree inferred using SILVA-based alignment of the "long" dataset [file biodiversity_data_journal-4-e10021-s035.jpg]

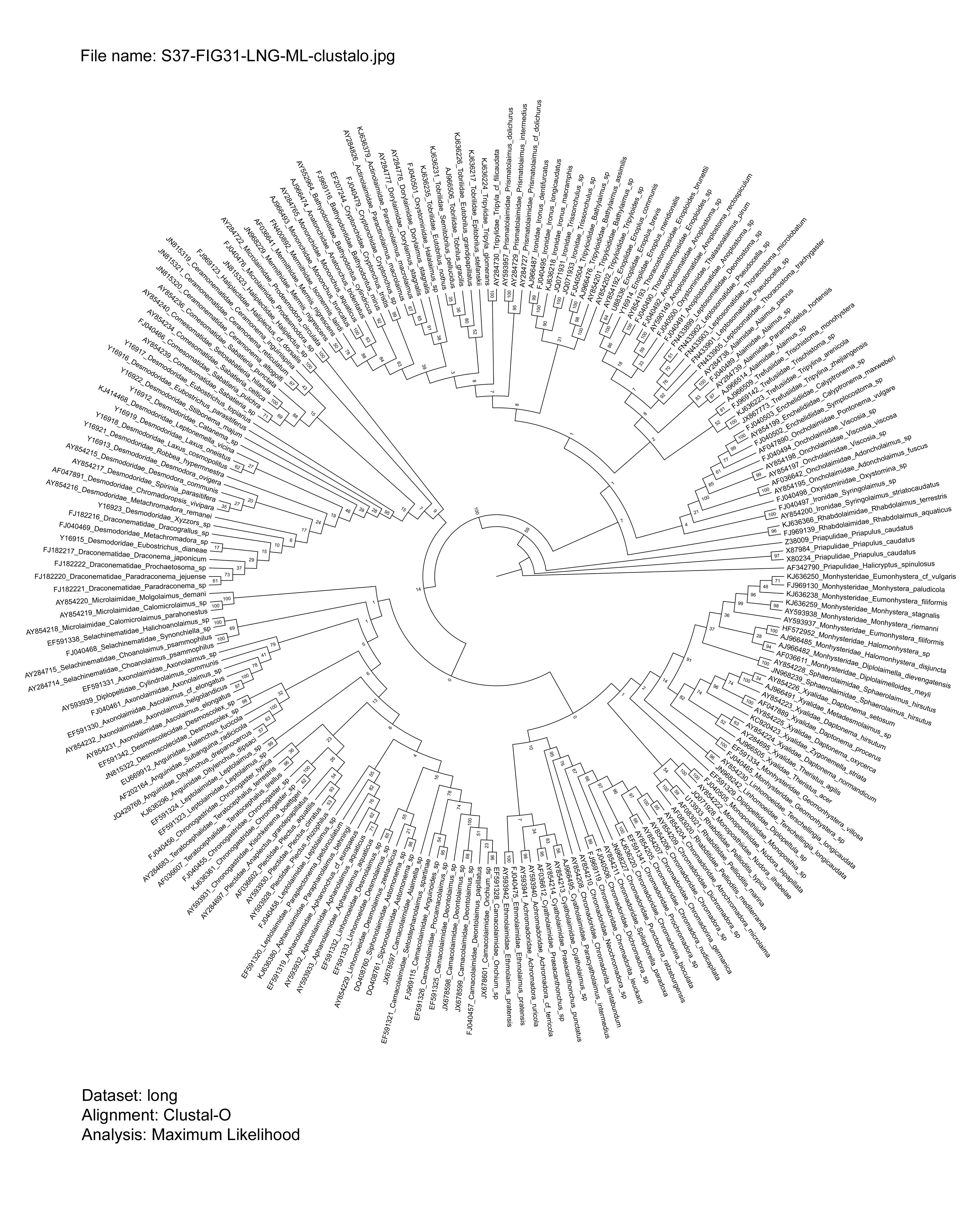

Supplement: Supplementary material 37 — Maximum likelihood tree inferred using Clustal-O alignment of the "long" dataset [file biodiversity_data_journal-4-e10021-s037.jpg]

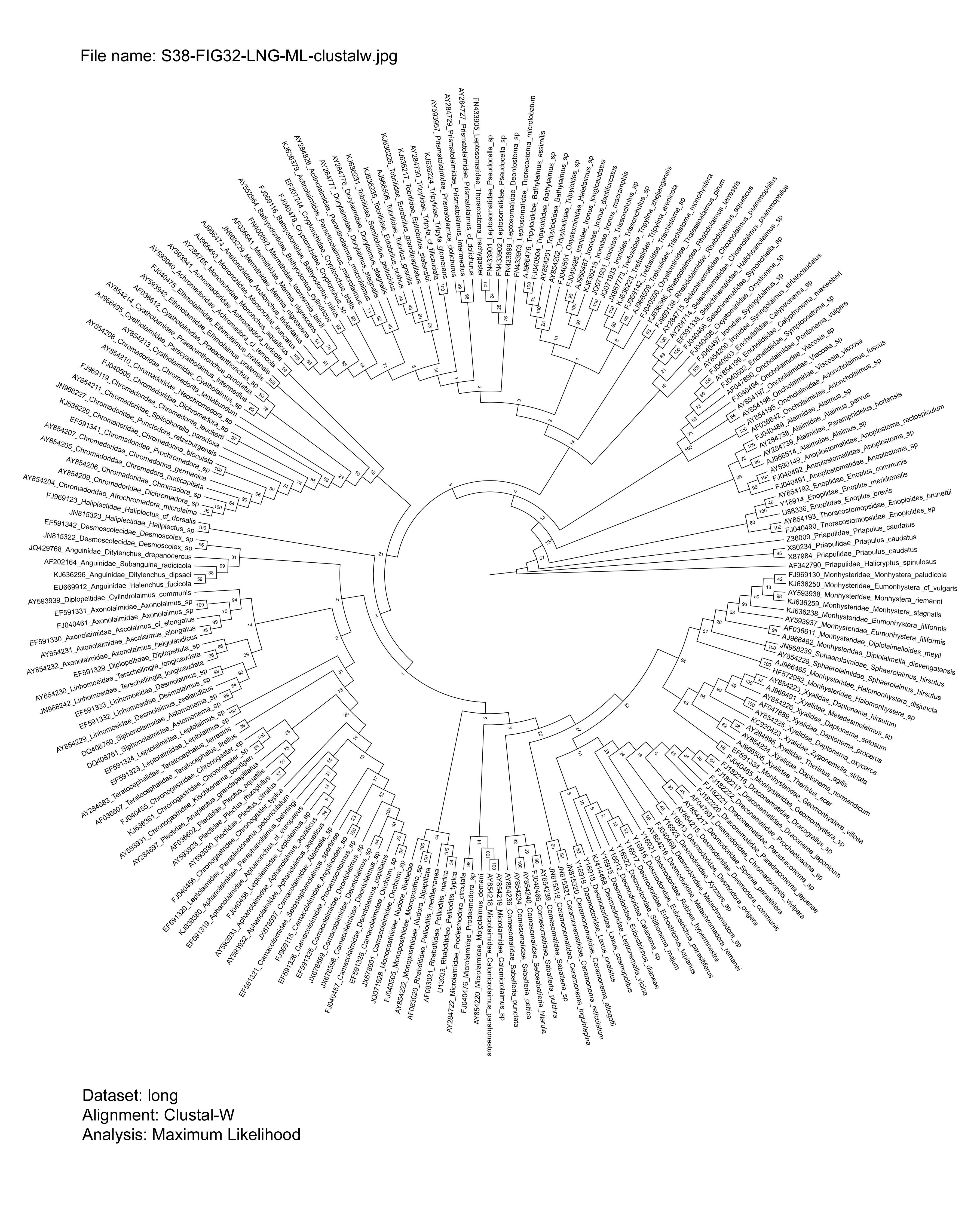

Supplement: Supplementary material 38 — Maximum likelihood tree inferred using Clustal-W alignment of the "long" dataset [file biodiversity_data_journal-4-e10021-s038.jpg]

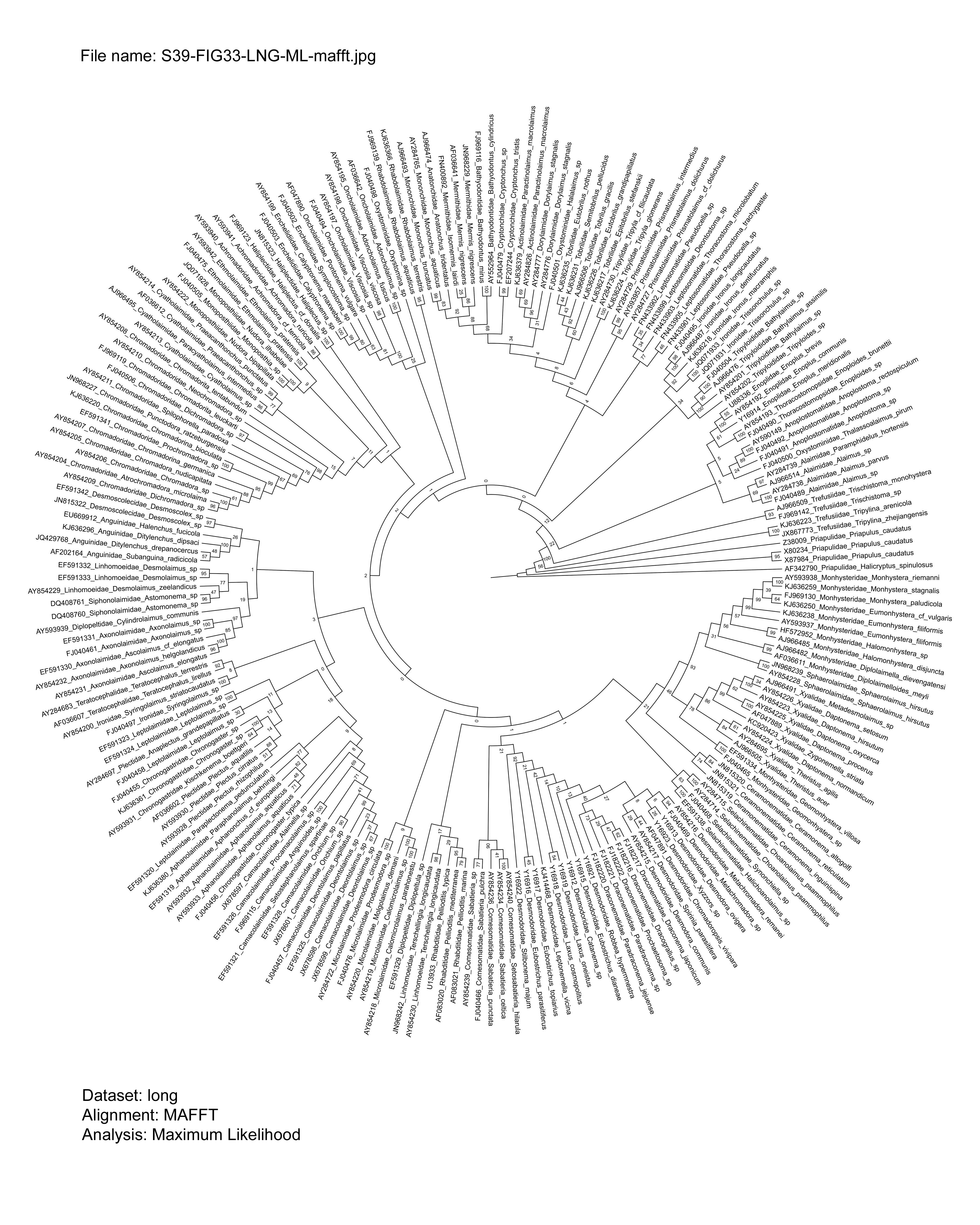

Supplement: Supplementary material 39 — Maximum likelihood tree inferred using MAFFT alignment of the "long" dataset [file biodiversity_data_journal-4-e10021-s039.jpg]

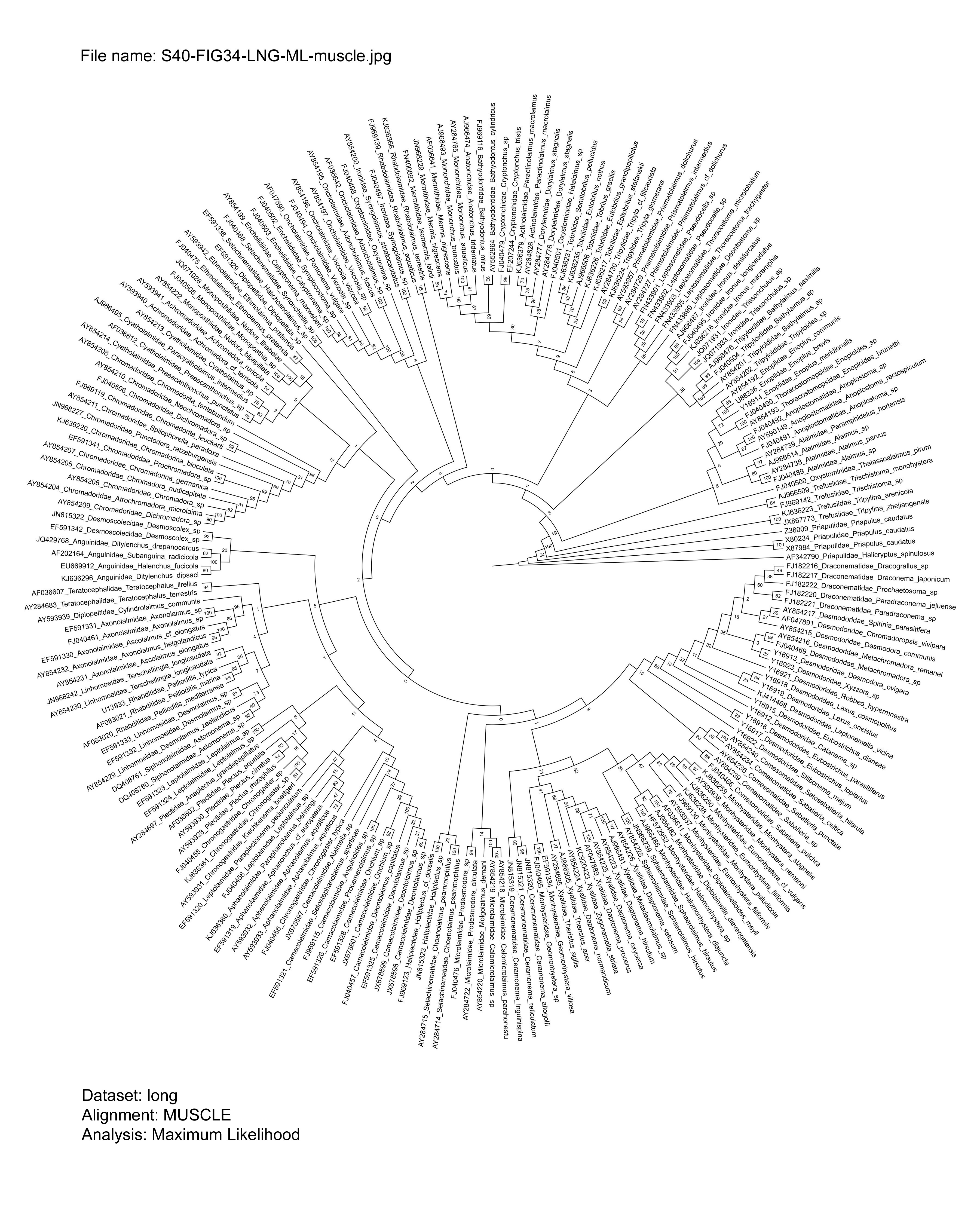

Supplement: Supplementary material 40 — Maximum likelihood tree inferred using MUSCLE alignment of the "long" dataset [file biodiversity_data_journal-4-e10021-s040.jpg]

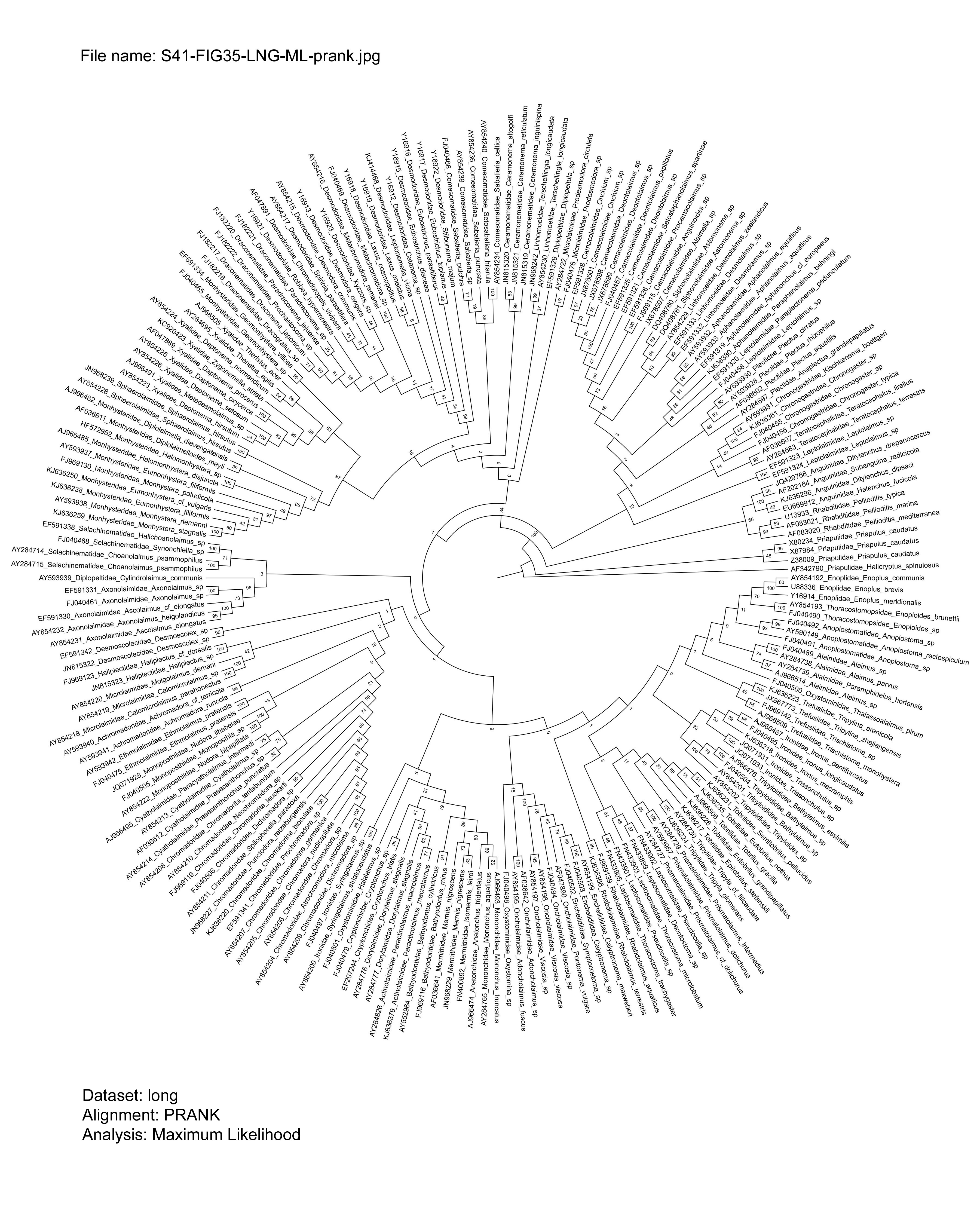

Supplement: Supplementary material 41 — Maximum likelihood tree inferred using PRANK alignment of the "long" dataset [file biodiversity_data_journal-4-e10021-s041.jpg]

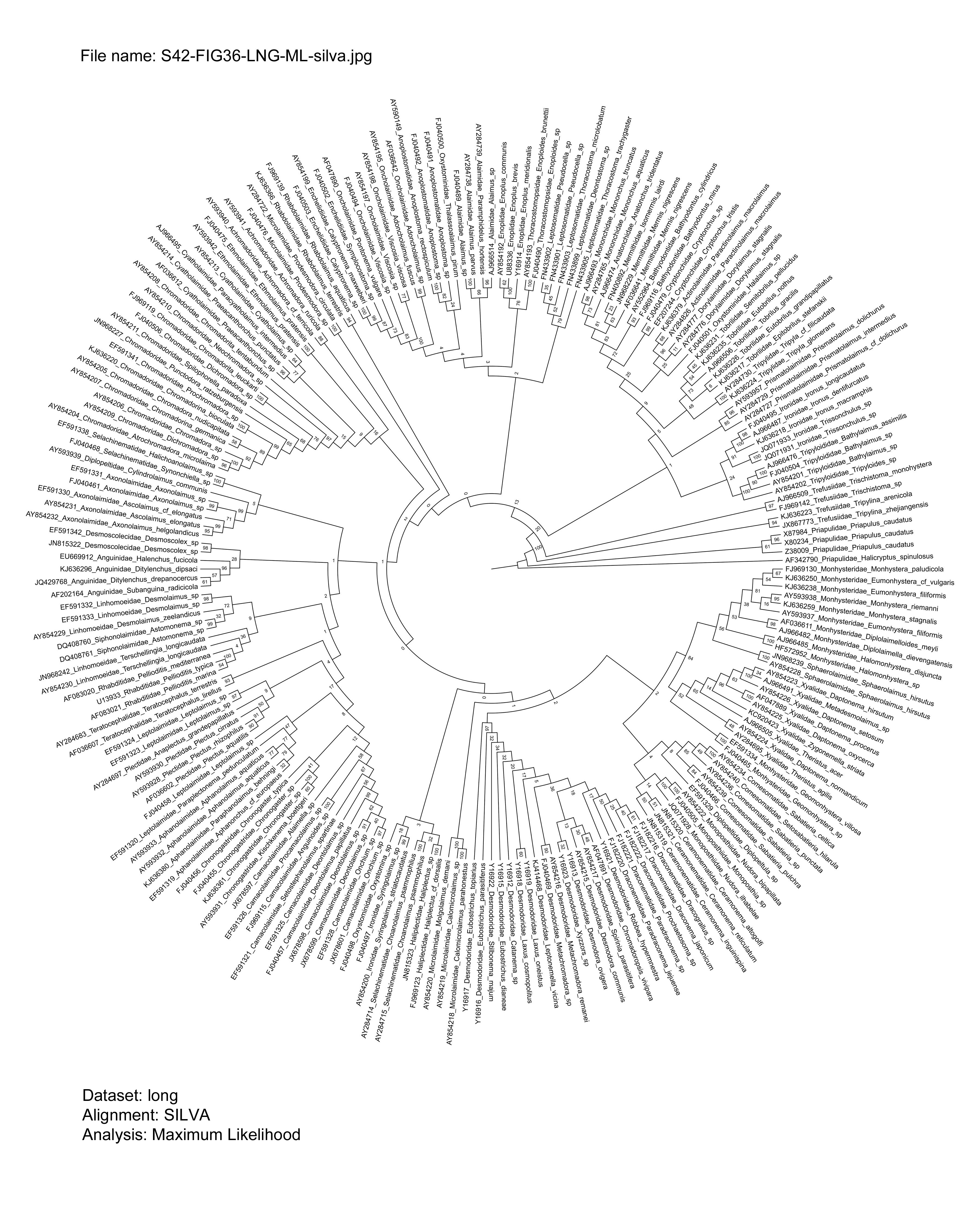

Supplement: Supplementary material 42 — Maximum likelihood tree inferred using SILVA-based alignment of the "long" dataset [file biodiversity_data_journal-4-e10021-s042.jpg]
