# Supplementary material for: Metabarcoding of marine nematodes – evaluation of reference datasets used in tree-based taxonomy assignment approach
Source: Biodivers Data J. 2016 Sep 21;(4):e10021. doi: 10.3897/BDJ.4.e10021 (PMC5136706; doi:10.3897/BDJ.4.e10021)
Supplement: Supplementary material 8 — Table S2. Resolution and bootstrap support (for monophyletic clades) of nematode families based on Neighbor joining analyses of different multiple sequence alignments of complete dataset (POL - polyphyletic, PAR - paraphyletic) [file biodiversity_data_journal-4-e10021-s008.pdf]

File name: S08-TABS2-ALL-NJ.pdf

Table S2. Resolution and bootstrap support (for monophyletic clades) of nematode families based on Neighbor joining analyses of different multiple sequence alignments of complete dataset (POL - polyphyletic, PAR - paraphyletic). Resolved clades are highlighted in grey.

Taxon (family or *superfamily)	number of species	Clustal-O	Clustal-W	MAFFT	MUSCLE	PRANK	SILVA
Anguinidae	4	100	100	100	100	100	99
Rhabditidae	3	100	100	99	100	100	100
Teratocephalidae	2	100	100	100	100	100	97
Plectidae	4	99	95	98	93	85	88
Chronogastridae	5	POL	POL	POL	POL	POL	POL
Aphanolaimidae	4	60	95	79	73	73	86
Leptolaimidae	4	POL	POL	POL	POL	POL	POL
Camacolaimidae	10	POL	POL	POL	POL	POL	POL
Axonolaimidae	8	POL	POL	POL	POL	POL	POL
Diplopletidae	2	POL	POL	POL	POL	POL	POL
Comesomatidae	6	95	94	93	86	86	94
Monhysteridae	12	PAR	PAR	PAR	PAR	PAR	PAR
Xyalidae	10	37	POL	62	33	41	35
Sphaerolaimidae	2	100	100	100	100	100	100
Linhomoeidae	7	POL	POL	POL	POL	POL	POL
Siphonolaimidae	2	92	100	100	99	100	100
Ceramonematidae	4	74	94	78	59	80	43
Desmoscolecidae	2	85	92	90	85	98	83
Draconematidae	5	49	43	35	66	58	48
Desmodoridae	21	PAR	PAR	PAR	PAR	PAR	PAR
Microlaimidae	5	POL	POL	POL	POL	POL	POL
Monoposthiidae	4	POL	POL	POL	POL	POL	POL
Selachinematidae	6	POL	POL	POL	POL	POL	POL
Ethmolaimidae	2	100	100	100	100	100	100
Achromadoridae	2	100	100	100	100	100	100
Cyatholaimidae	7	POL	POL	POL	POL	POL	POL
Chromadoridae	14	56	87	91	96	89	75
Haliplectidae	2	100	100	100	100	100	100
Dorylaimoidea*	4	100	100	100	100	100	100
Mononchoidea*	3	96	100	100	99	99	96
Bathyodontidae	2	100	100	100	100	100	100
Cryptonchidae	2	100	100	100	100	100	100
Mermithidae	3	PAR	PAR	PAR	PAR	PAR	PAR

Taxon (family or *superfamily)	number of species	Clustal-O	Clustal-W	MAFFT	MUSCLE	PRANK	SILVA
Prismatolaimidae	3	97	98	98	98	97	90
Tripylidae	3	100	100	100	100	100	100
Tobrilidae	5	POL	POL	45	39	65	31
Oncholaimidae	10	POL	POL	POL	POL	POL	POL
Enchelidiidae	7	POL	POL	POL	POL	POL	POL
Enoplidae	3	PAR	PAR	PAR	PAR	PAR	PAR
Thoracostomopsidae	13	PAR	PAR	PAR	PAR	PAR	PAR
Phanodermatidae	4	POL	27	PAR	PAR	PAR	POL
Anticomidae	4	POL	POL	POL	POL	POL	POL
Leptosomatidae	7	100	100	100	98	100	100
Trefusiidae	8	POL	POL	POL	POL	POL	POL
Tripyloididae	6	100	100	100	100	100	100
Anoplostomatidae	5	POL	POL	POL	POL	POL	POL
Oxystominidae	15	POL	POL	POL	POL	POL	POL
Alaimidae	4	85	95	97	94	91	98
Ironidae	8	POL	POL	POL	POL	POL	POL
Rhabdolaimidae	2	100	100	100	100	100	100
