# Supplementary material for: Metabarcoding of marine nematodes – evaluation of reference datasets used in tree-based taxonomy assignment approach
Source: Biodivers Data J. 2016 Sep 21;(4):e10021. doi: 10.3897/BDJ.4.e10021 (PMC5136706; doi:10.3897/BDJ.4.e10021)
Supplement: Supplementary material 15 — Table S3. Resolution and bootstrap support (for monophyletic clades) of nematode families based on Maximum likelihood analyses of different multiple sequence alignments of complete dataset (POL - polyphyletic, PAR - paraphyletic) [file biodiversity_data_journal-4-e10021-s015.pdf]

File name: S15-TABS3-ALL-ML.pdf

Table S3. Resolution and bootstrap support (for monophyletic clades) of nematode families based on Maximum likelihood analyses of different multiple sequence alignments of complete dataset (POL - polyphyletic, PAR - paraphyletic). Resolved clades are highlighted in grey.

Taxon (family or *superfamily)	number of species	Clustal-O	Clustal-W	MAFFT	MUSCLE	PRANK	SILVA
Anguinidae	4	99	98	99	100	100	96
Rhabditidae	3	100	99	84	92	98	100
Teratocephalidae	2	97	98	95	95	97	88
Plectidae	4	50	70	70	52	PAR	46
Chronogastridae	5	POL	POL	POL	POL	POL	POL
Aphanolaimidae	4	64	76	76	PAR	79	81
Leptolaimidae	4	POL	POL	POL	POL	POL	POL
Camacolaimidae	10	52	PAR	42	43	PAR	48
Axonolaimidae	8	POL	POL	POL	POL	POL	POL
Diplopletidae	2	POL	POL	POL	POL	POL	POL
Comesomatidae	6	92	80	92	88	88	86
Monhysteridae	12	PAR	PAR	PAR	PAR	PAR	PAR
Xyalidae	10	71	61	86	61	78	57
Sphaerolaimidae	2	100	100	100	100	100	100
Linhomoeidae	7	POL	POL	POL	POL	POL	POL
Siphonolaimidae	2	88	99	98	96	96	99
Ceramonematidae	4	64	92	87	56	72	PAR
Desmoscolecidae	2	98	98	97	92	100	96
Draconematidae	5	POL	POL	POL	44	44	42
Desmodoridae	21	PAR	PAR	PAR	PAR	PAR	PAR
Microlaimidae	5	POL	POL	PAR	POL	PAR	POL
Monoposthiidae	4	POL	POL	POL	POL	POL	POL
Selachinematidae	6	POL	POL	POL	POL	POL	POL
Ethmolaimidae	2	100	100	100	100	100	100
Achromadoridae	2	93	92	89	92	92	90
Cyatholaimidae	7	POL	POL	POL	POL	POL	POL
Chromadoridae	14	62	PAR	PAR	70	89	65
Haliplectidae	2	100	100	100	100	100	99
Dorylaimoidea*	4	100	100	100	100	100	100
Mononchoidea*	3	44	90	80	76	89	71
Bathyodontidae	2	88	96	92	93	92	92
Cryptonchidae	2	99	98	99	99	99	99
Mermithidae	3	80	74	81	87	65	67

Taxon (family or *superfamily)	number of species	Clustal-O	Clustal-W	MAFFT	MUSCLE	PRANK	SILVA
Prismatolaimidae	3	97	96	95	97	98	86
Tripylidae	3	100	100	99	99	100	100
Tobrilidae	5	30	57	52	PAR	87	69
Oncholaimidae	10	POL	POL	POL	POL	POL	POL
Enchelidiidae	7	67	60	PAR	45	60	61
Enoplidae	3	71	65	60	68	72	42
Thoracostomopsidae	13	PAR	PAR	PAR	PAR	PAR	PAR
Phanodermatidae	4	PAR	48	PAR	PAR	PAR	PAR
Anticomidae	4	POL	POL	POL	POL	POL	POL
Leptosomatidae	7	60	82	66	46	88	78
Trefusiidae	8	POL	POL	POL	POL	POL	POL
Tripyloididae	6	100	100	100	100	100	100
Anoplostomatidae	5	POL	POL	POL	POL	POL	POL
Oxystominidae	15	PAR	PAR	PAR	PAR	PAR	PAR
Alaimidae	4	81	86	92	91	84	85
Ironidae	8	POL	POL	POL	POL	POL	POL
Rhabdolaimidae	2	94	100	96	98	93	93
