# Supplementary material for: Metabarcoding of marine nematodes – evaluation of reference datasets used in tree-based taxonomy assignment approach
Source: Biodivers Data J. 2016 Sep 21;(4):e10021. doi: 10.3897/BDJ.4.e10021 (PMC5136706; doi:10.3897/BDJ.4.e10021)
Supplement: Supplementary material 22 — Table S4. Resolution and bootstrap support (for monophyletic clades) of nematode families based on Neighbor joining analyses of different multiple sequence alignments of "filtered" dataset (POL - polyphyletic, PAR - paraphyletic) [file biodiversity_data_journal-4-e10021-s022.pdf]

File name: S22-TABS4-FILT-NJ.pdf

Table S4. Resolution and bootstrap support (for monophyletic clades) of nematode families based on Neighbor joining analyses of different multiple sequence alignments of "filtered" dataset (POL - polyphyletic, PAR - paraphyletic). Resolved clades are highlighted in grey.

Taxon (family or *superfamily)	number of species	Clustal-O	Clustal-W	MAFFT	MUSCLE	PRANK	SILVA
Anguinidae	4	100	100	100	100	100	99
Rhabditidae	3	100	100	100	100	100	100
Teratocephalidae	2	100	100	100	100	100	97
Plectidae	4	87	96	96	88	86	85
Chronogastridae	5	POL	POL	POL	POL	POL	POL
Aphanolaimidae	4	68	94	73	71	71	86
Leptolaimidae	4	POL	POL	POL	POL	POL	POL
Camacolaimidae	10	PAR	PAR	PAR	PAR	PAR	POL
Axonolaimidae	7	PAR	57	47	42	PAR	PAR
Diplopletidae	2	POL	POL	POL	POL	POL	POL
Comesomatidae	6	97	95	96	79	91	93
Monhysteridae	12	PAR	PAR	PAR	PAR	PAR	PAR
Xyalidae	10	50	PAR	65	89	23	37
Sphaerolaimidae	2	100	100	100	100	100	100
Linhomoeidae	7	POL	POL	POL	POL	POL	POL
Siphonolaimidae	2	96	100	100	100	100	100
Ceramonematidae	4	69	91	83	69	82	45
Desmoscolecidae	2	88	95	92	93	89	84
Draconematidae	5	44	46	38	70	66	45
Desmodoridae	21	PAR	PAR	PAR	PAR	PAR	PAR
Microlaimidae	5	POL	POL	POL	POL	POL	POL
Monoposthiidae	3	100	100	100	100	100	100
Selachinematidae	5	32	36	44	33	19	26
Ethmolaimidae	2	100	100	100	100	100	100
Achromadoridae	2	100	100	100	100	100	100
Cyatholaimidae	4	93	98	90	99	96	95
Chromadoridae	14	87	88	94	88	91	80
Haliplectidae	2	100	100	100	100	100	100
Dorylaimoidea*	4	100	100	100	100	100	100
Mononchoidea*	3	96	100	99	99	99	96
Bathyodontidae	2	100	100	100	100	100	100
Cryptonchidae	2	100	100	100	100	100	100
Mermithidae	3	PAR	PAR	PAR	PAR	PAR	PAR

Taxon (family or *superfamily)	number of species	Clustal-O	Clustal-W	MAFFT	MUSCLE	PRANK	SILVA
Prismatolaimidae	3	98	100	99	99	95	92
Tripylidae	3	100	100	100	100	100	100
Tobrilidae	5	48	64	38	23	67	72
Oncholaimidae	9	PAR	PAR	PAR	PAR	PAR	PAR
Enchelidiidae	7	PAR	PAR	PAR	PAR	PAR	PAR
Enoplidae	3	100	100	100	100	100	100
Thoracostomopsidae	13	93	95	97	94	97	96
Phanodermatidae	4	28	31	PAR	POL	PAR	POL
Anticomidae	3	100	100	100	100	100	100
Leptosomatidae	7	100	100	100	99	100	99
Trefusiidae	8	POL	POL	POL	POL	POL	POL
Tripyloididae	6	100	100	100	100	100	100
Anoplostomatidae	5	POL	POL	POL	POL	POL	POL
Oxystominidae	15	POL	POL	POL	PAR	POL	POL
Alaimidae	4	97	99	97	96	94	98
Ironidae	8	POL	POL	POL	POL	POL	POL
Rhabdolaimidae	2	100	100	100	100	100	100
