# Supplementary material for: Metabarcoding of marine nematodes – evaluation of reference datasets used in tree-based taxonomy assignment approach
Source: Biodivers Data J. 2016 Sep 21;(4):e10021. doi: 10.3897/BDJ.4.e10021 (PMC5136706; doi:10.3897/BDJ.4.e10021)
Supplement: Supplementary material 29 — Table S5. Resolution and bootstrap support (for monophyletic clades) of nematode families based on Maximum likelihood analyses of different multiple sequence alignments of "filtered" dataset (POL - polyphyletic, PAR - paraphyletic) [file biodiversity_data_journal-4-e10021-s029.pdf]

File name: S29-TABS5-FILT-ML.pdf

Table S5. Resolution and bootstrap support (for monophyletic clades) of nematode families based on Maximum likelihood analyses of different multiple sequence alignments of "filtered" dataset (POL - polyphyletic, PAR - paraphyletic). Resolved clades are highlighted in grey.

Taxon (family or *superfamily)	number of species	Clustal-O	Clustal-W	MAFFT	MUSCLE	PRANK	SILVA
Anguinidae	4	98	99	99	100	100	96
Rhabditidae	3	100	100	75	87	93	100
Teratocephalidae	2	96	99	96	96	97	92
Plectidae	4	41	73	56	45	PAR	48
Chronogastridae	5	POL	POL	POL	POL	POL	POL
Aphanolaimidae	4	75	80	69	78	82	82
Leptolaimidae	4	POL	POL	POL	POL	POL	POL
Camacolaimidae	10	74	71	40	37	42	46
Axonolaimidae	7	47	65	PAR	43	30	38
Diplopletidae	2	POL	POL	POL	POL	POL	POL
Comesomatidae	6	78	95	92	87	89	86
Monhysteridae	12	PAR	PAR	PAR	PAR	PAR	PAR
Xyalidae	10	74	62	87	60	72	56
Sphaerolaimidae	2	100	100	100	100	100	100
Linhomoeidae	7	POL	POL	POL	POL	POL	POL
Siphonolaimidae	2	94	100	98	96	99	99
Ceramonematidae	4	62	96	87	74	82	PAR
Desmoscolecidae	2	98	99	96	93	97	98
Draconematidae	5	POL	POL	36	39	46	42
Desmodoridae	21	PAR	PAR	PAR	PAR	PAR	PAR
Microlaimidae	5	POL	POL	POL	POL	POL	POL
Monoposthiidae	3	100	100	100	100	100	100
Selachinematidae	5	24	70	67	47	49	POL
Ethmolaimidae	2	100	100	100	100	100	100
Achromadoridae	2	97	91	82	97	98	90
Cyatholaimidae	4	88	98	94	91	96	95
Chromadoridae	14	94	82	95	96	95	94
Haliplectidae	2	100	100	100	100	100	100
Dorylaimoidea*	4	99	100	100	100	100	99
Mononchoidea*	3	66	86	80	76	80	69
Bathyodontidae	2	90	91	93	94	81	93
Cryptonchidae	2	99	99	100	98	99	99
Mermithidae	3	80	78	82	86	76	68

Taxon (family or *superfamily)	number of species	Clustal-O	Clustal-W	MAFFT	MUSCLE	PRANK	SILVA
Prismatolaimidae	3	95	98	94	98	97	87
Tripylidae	3	100	100	98	99	99	100
Tobrilidae	5	61	57	POL	POL	87	66
Oncholaimidae	9	PAR	PAR	PAR	PAR	PAR	PAR
Enchelidiidae	7	65	62	55	PAR	56	64
Enoplidae	3	100	100	100	100	100	100
Thoracostomopsidae	13	93	97	83	90	97	91
Phanodermatidae	4	PAR	53	PAR	39	PAR	PAR
Anticomidae	3	100	100	100	100	100	100
Leptosomatidae	7	58	85	61	57	89	79
Trefusiidae	8	POL	POL	POL	POL	10	POL
Tripyloididae	6	100	100	100	100	100	100
Anoplostomatidae	5	POL	POL	POL	POL	POL	POL
Oxystominidae	15	PAR	PAR	PAR	PAR	PAR	PAR
Alaimidae	4	91	88	91	89	81	86
Ironidae	8	POL	POL	POL	POL	POL	POL
Rhabdolaimidae	2	96	100	95	96	96	94
