# Supplementary material for: Metabarcoding of marine nematodes – evaluation of reference datasets used in tree-based taxonomy assignment approach
Source: Biodivers Data J. 2016 Sep 21;(4):e10021. doi: 10.3897/BDJ.4.e10021 (PMC5136706; doi:10.3897/BDJ.4.e10021)
Supplement: Supplementary material 36 — Table S6. Resolution and bootstrap support (for monophyletic clades) of nematode families based on Neighbor joining analyses of different multiple sequence alignments of "long" dataset (POL - polyphyletic, PAR - paraphyletic) [file biodiversity_data_journal-4-e10021-s036.pdf]

File name: S36-TABS6-LNG-NJ.pdf

Table S6. Resolution and bootstrap support (for monophyletic clades) of nematode families based on Neighbor joining analyses of different multiple sequence alignments of "long" dataset (POL - polyphyletic, PAR - paraphyletic). Resolved clades are highlighted in grey.

Taxon (family or *superfamily)	number of species	Clustal-O	Clustal-W	MAFFT	MUSCLE	PRANK	SILVA
Anguinidae	4	100	100	100	100	100	100
Rhabditidae	3	100	100	100	100	100	100
Teratocephalidae	2	100	100	100	100	100	97
Plectidae	4	88	97	87	88	83	85
Chronogastridae	4	38	23	26	34	41	57
Aphanolaimidae	4	77	96	79	86	81	91
Leptolaimidae	4	POL	POL	POL	POL	POL	POL
Camacolaimidae	10	82	80	65	67	77	69
Axonolaimidae	5	76	85	79	53	78	77
Diplopletidae	2	POL	POL	POL	POL	POL	POL
Comesomatidae	5	92	96	94	90	93	94
Monhysteridae	12	PAR	PAR	PAR	PAR	PAR	PAR
Xyalidae	9	54	44	71	77	71	66
Sphaerolaimidae	2	100	100	100	100	100	100
Linhomoeidae	5	POL	POL	POL	POL	POL	POL
Siphonolaimidae	2	98	100	100	96	100	100
Ceramonematidae	3	54	96	87	91	98	45
Desmoscolecidae	2	94	81	91	77	90	82
Draconematidae	5	64	71	86	71	89	65
Desmodoridae	16	PAR	PAR	PAR	PAR	PAR	PAR
Microlaimidae	5	POL	POL	POL	19	POL	POL
Monoposthiidae	3	100	100	100	100	100	100
Selachinematidae	4	59	65	64	26	52	27
Ethmolaimidae	2	100	100	100	100	100	100
Achromadoridae	2	100	100	100	100	100	100
Cyatholaimidae	4	90	98	95	97	90	90
Chromadoridae	13	95	96	93	81	99	88
Haliplectidae	2	100	100	100	100	100	100
Dorylaimoidea*	4	100	100	100	100	100	100
Mononchoidea*	3	97	99	100	100	99	97
Bathyodontidae	2	100	100	100	100	100	100
Cryptonchidae	2	100	100	100	100	100	100
Mermithidae	3	PAR	PAR	PAR	PAR	PAR	PAR

Taxon (family or *superfamily)	number of species	Clustal-O	Clustal-W	MAFFT	MUSCLE	PRANK	SILVA
Prismatolaimidae	3	92	98	95	98	95	91
Tripylidae	2	100	100	100	100	100	100
Tobrilidae	5	41	54	59	45	51	81
Oncholaimidae	6	PAR	PAR	PAR	PAR	PAR	PAR
Enchelidiidae	3	99	98	97	98	99	96
Enoplidae	3	100	100	100	100	100	100
Thoracostomopsidae	2	100	100	100	100	100	100
Leptosomatidae	5	100	100	99	99	100	99
Trefusiidae	4	93	99	87	91	79	94
Tripyloididae	4	100	100	100	100	100	100
Anoplostomatidae	3	79	99	98	97	99	98
Oxystominidae	3	POL	POL	POL	POL	POL	POL
Alaimidae	4	97	96	92	97	92	97
Ironidae	7	POL	POL	POL	POL	POL	POL
Rhabdolaimidae	2	100	100	100	100	100	100
