# Supplementary material for: Metabarcoding of marine nematodes – evaluation of reference datasets used in tree-based taxonomy assignment approach
Source: Biodivers Data J. 2016 Sep 21;(4):e10021. doi: 10.3897/BDJ.4.e10021 (PMC5136706; doi:10.3897/BDJ.4.e10021)
Supplement: Supplementary material 43 — Table S7. Resolution and bootstrap support (for monophyletic clades) of nematode families based on Maximum likelihood analyses of different multiple sequence alignments of "long" dataset (POL - polyphyletic, PAR - paraphyletic) [file biodiversity_data_journal-4-e10021-s043.pdf]

File name: S43-TABS7-LNG-ML.pdf

Table S7. Resolution and bootstrap support (for monophyletic clades) of nematode families based on Maximum likelihood analyses of different multiple sequence alignments of "long" dataset (POL - polyphyletic, PAR - paraphyletic). Resolved clades are highlighted in grey.

Taxon (family or *superfamily)	number of species	Clustal-O	Clustal-W	MAFFT	MUSCLE	PRANK	SILVA
Anguinidae	4	100	99	100	100	100	96
Rhabditidae	3	100	100	95	85	99	100
Teratocephalidae	2	96	99	92	94	99	93
Plectidae	4	54	75	PAR	PAR	45	50
Chronogastridae	4	POL	POL	POL	POL	49	41
Aphanolaimidae	4	76	PAR	82	80	81	79
Leptolaimidae	4	POL	POL	POL	POL	POL	POL
Camacolaimidae	10	78	77	69	78	73	58
Axonolaimidae	5	PAR	75	85	66	73	71
Diplopletidae	2	POL	POL	POL	POL	POL	POL
Comesomatidae	5	88	92	90	83	86	85
Monhysteridae	12	PAR	PAR	PAR	PAR	PAR	PAR
Xyalidae	9	74	65	86	69	88	65
Sphaerolaimidae	2	100	100	100	100	100	100
Linhomoeidae	5	POL	PAR	POL	POL	POL	POL
Siphonolaimidae	2	98	99	96	95	99	99
Ceramonematidae	3	43	95	74	89	99	50
Desmoscolecidae	2	98	96	97	92	95	98
Draconematidae	5	PAR	65	73	60	81	50
Desmodoridae	16	PAR	PAR	PAR	PAR	PAR	PAR
Microlaimidae	5	POL	14	9	14	POL	POL
Monoposthiidae	3	100	100	100	100	100	100
Selachinematidae	4	69	69	63	POL	71	100
Ethmolaimidae	2	100	100	100	99	100	100
Achromadoridae	2	98	93	86	92	98	88
Cyatholaimidae	4	83	78	77	83	75	77
Chromadoridae	13	95	98	98	96	99	97
Haliplectidae	2	100	100	100	100	100	100
Dorylaimoidea*	4	91	95	96	98	99	96
Mononchoidea*	3	63	88	83	91	89	73
Bathyodontidae	2	92	92	100	92	91	93
Cryptonchidae	2	99	99	99	98	99	99
Mermithidae	3	78	78	86	79	73	63

Taxon (family or *superfamily)	number of species	Clustal-O	Clustal-W	MAFFT	MUSCLE	PRANK	SILVA
Prismatolaimidae	3	95	96	95	94	95	85
Tripylidae	2	100	100	100	99	100	100
Tobrilidae	5	52	58	60	53	81	73
Oncholaimidae	6	PAR	PAR	PAR	PAR	PAR	PAR
Enchelidiidae	3	99	99	99	99	99	99
Enoplidae	3	100	100	100	100	100	100
Thoracostomopsidae	2	100	100	100	100	100	100
Leptosomatidae	5	92	76	77	69	84	79
Trefusiidae	4	52	80	PAR	PAR	40	PAR
Tripyloididae	4	100	100	100	100	100	100
Anoplostomatidae	3	PAR	95	89	87	93	82
Oxystominidae	3	POL	POL	POL	POL	POL	POL
Alaimidae	4	83	78	69	80	74	66
Ironidae	7	POL	POL	POL	POL	POL	POL
Rhabdolaimidae	2	96	93	95	95	96	94
